# Novel Inhibitors to MmpL3 Transporter of *Mycobacterium tuberculosis* by Structure-Based High-Throughput Virtual Screening and Molecular Dynamics Simulations

**DOI:** 10.1021/acsomega.3c08401

**Published:** 2024-03-12

**Authors:** Hetanshi Choksi, Justin Carbone, Nicholas J. Paradis, Lucas Bennett, Candice Bui-Linh, Chun Wu

**Affiliations:** Department of Molecular & Cellular Biosciences, College of Science and Mathematics, Rowan University, Glassboro, New Jersey 08028, United States

## Abstract

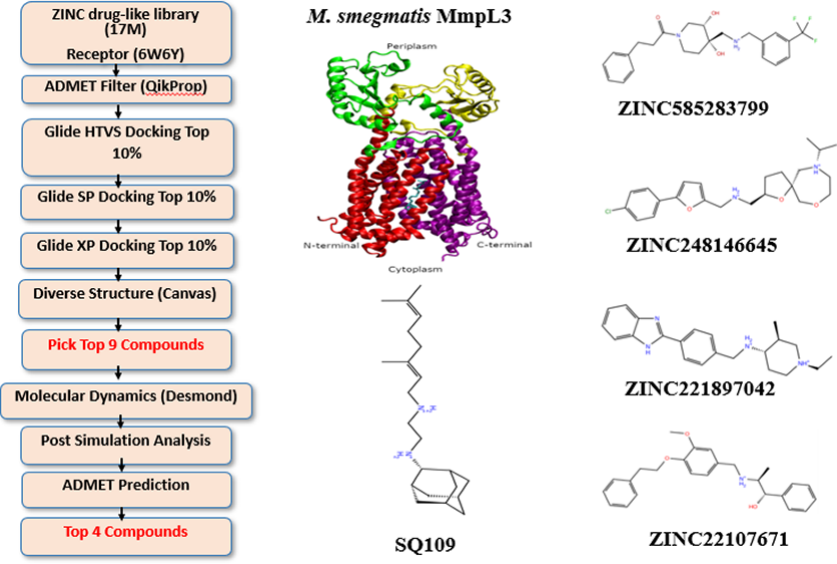

Tuberculosis (TB)-causing bacterium *Mycobacterium tuberculosis* (Mtb) utilizes mycolic acids for building the mycobacterial cell wall, which is critical in providing defense against external factors and resisting antibiotic action. MmpL3 is a secondary resistance nodulation division transporter that facilitates the coupled transport of mycolic acid precursor into the periplasm using the proton motive force, thus making it an attractive drug target for TB infection. In 2019, X-ray crystal structures of MmpL3 from *M. smegmatis* were solved with a promising inhibitor SQ109, which showed promise against drug-resistant TB in Phase II clinical trials. Still, there is a pressing need to discover more effective MmpL3 inhibitors to counteract rising antibiotic resistance. In this study, structure-based high-throughput virtual screening combined with molecular dynamics (MD) simulations identified potential novel MmpL3 inhibitors. Approximately 17 million compounds from the ZINC15 database were screened against the SQ109 binding site on the MmpL3 protein using drug property filters and glide XP docking scores. From this, the top nine compounds and the MmpL3–SQ109 crystal complex structure each underwent 2 × 200 ns MD simulations to probe the inhibitor binding energetics to MmpL3. Four of the nine compounds exhibited stable binding properties and favorable drug properties, suggesting these four compounds could be potential novel inhibitors of MmpL3 for *M. tuberculosis*.

## 1 Introduction

Tuberculosis (TB), caused by the bacterial agent *Mycobacterium tuberculosis* (*Mtb*), is the second leading infectious disease killer after COVID-19, which caused 1.5 million deaths and 10 million infections worldwide.^1^ Treatment for TB has become increasingly difficult with the continued emergence of multidrug-resistant, extreme drug-resistant, and totally drug-resistant *Mtb* strains^2^ In fact, ∼20–30% of reported cases are drug-resistant, leading to longer treatment courses (18–20 months). The current treatment for TB is a rigorous 2-month administration of the chemotherapeutic agents isoniazid (INH), rifampicin (Rif), ethambutol (EMB), and pyrazinamide (Figure S1) followed by a 4-month course of INH and Rif alone.^3^ Side effects from this treatment can be severe and result in poor overall patient compliance, contributing to the increase of drug resistance. These issues demand the identification of alternative and efficacious anti-TB drug treatments to combat drug-resistant TB, reduce treatment duration, and improve patient compliance.

Mycobacterial membrane protein large 3 (MmpL3) is an essential protein for the survival of *MtB* as trehalose monomycolate (TMM) transport leads to the synthesis of mycolic acids that create the waxy outer membrane coating unique in *Mycobacterium,* making the membrane impermeable to many external environmental factors and antibacterials.^4−6^ MmpL3 is the key proton motive force (PMF)-dependent antiporter that couples inbound proton transport into the cytoplasm and outbound TMM transport across the membrane and into the periplasm for building the cell wall.^7,8^ MmpL3 is also unique, as it is the only resistance nodulation division (RND) transporter to function in a monomeric state, relying heavily on efficient proton transport. The MmpL3 gene is conserved across all available MmpL genome sequences, indicating that MmpL3 is essential for *Mtb* viability and that mutations to MmpL3 could prove lethal to *Mtb*. Gene knockout experiments revealed that inactivation of the MmpL3 gene led to a complete loss of viability, confirming that MmpL3 is vital for *Mtb* functionality.^9^ To this, EMB and INH, two MmpL3 small molecule inhibitors resulted in a decrease of TMM transport,^9^ suggesting that MmpL3 inhibitors are a promising therapeutic strategy against TB.

The experimental analog of EMB, SQ109 (Figure S2), is currently under Phase 2–3a clinical trials and has shown to be a promising new treatment for TB.^1,10^ SQ109 had decreased the incorporation of mycolic acids into the mycobacterial cell wall more efficiently than EMB and INH. Additionally, SQ109 accumulates in the pulmonary system, which is the primary site of *Mtb* infection.^11^ Currently, no FDA-approved drugs exist that inhibit MmpL3, even though SQ109 analogs^12^ molecules and various small molecule inhibitors besides SQ109 are currently being investigated, including indole-2-carboxamides (NITD-349), piperidinols (PIPD1), and benzimidazoles (C215)^13^ (Figure S3). The minimal inhibitory concentrations of SQ109 (0.78 μM), NITD-349 (0.023 μM), PIPD1 (1.28 μM), and C215 (16.0 μM) reveal that NITD-349 is the only drug that demonstrates significantly higher efficacy than SQ109.^14,15^

**Figure 1:**
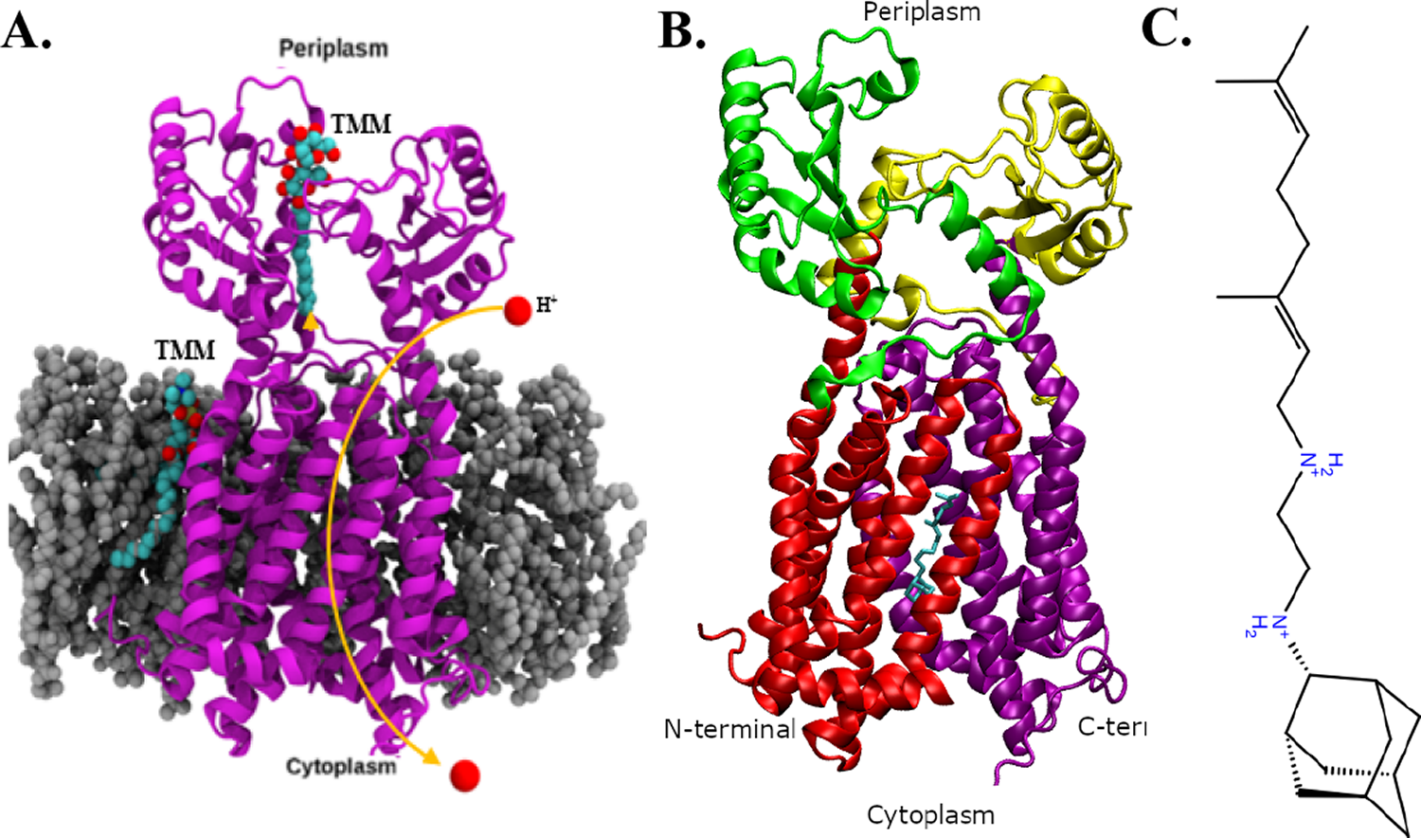
(A) Cartoon representation of *Mycobacteria smegmatis* MmpL3 (purple ribbon) embedded in a phospholipid bilayer (*PDB ID:*6AJF). (B) Ribbon representation with the binding site represented as the surface. (C) Chemical structure of SQ109.

Although limited structural data exist for *M. tuberculosis* MmpL3 transporters, recently Zhang et al.^16^ solved X-ray crystal structures of *M. smegmatis* MmpL3 in the unbound state (apo-form) and bound state (holo-form) with four known drug inhibitors, including SQ109 (PDB ID: 6AJG) (Figure 1A). It is important to note the high conservation observed between *M. tuberculosis* and *M. smegmatis* gene sequences,^17^ which share the conserved MmpL3 gene (Figure S4) (UniProt: P9WJV5 and I7G2R2, respectively).^18^ Thus, *M. smegmatis* MmpL3 is sufficient and reliable for discovering inhibitors for the *M. tuberculosis* MmpL3 transporter. Previously, rigorous analysis of these Apo and Holo structures suggest that SQ109 inhibits MmpL3 allosterically by occupying the proton channel embedded within the transmembrane domain and locking MmpL3 in an open state, allowing hindered proton flow but the reclosing and creation of the proton gradient and TMM transport are blocked.^19^ Using these conclusions on these structures, molecular dynamics (MD) simulation with binding free energy calculations were used to probe the structure–activity relationship for SQ109 analogs.^20^ In addition, virtual screening has been used in two studies to identify potential MmpL3 inhibitors using the crystal structure 6AJG (Table 1).

**Table 1:** Comparison between This Study and Previous Virtual Screening Studies

authors	protein PDB ID	ligand database	method	final hit compounds
Bhakhar et al.	6AJG	Asinex BioDesign Library (175,851)	E-pharmacophore based virtual screening (175,851)Glide Docking (4163)ADMET Analysis, Lipinski Filter (25)10 ns MD simulations (3 hits)	3
Chaitra et al.	6AJG	Literature and Database (1300)	structured-based pharmacophore screening (1300)glide docking (65)MM-GBSA and IFD docking (25)ADMET analysis (10)100 ns MD simulations (2 hits)	2
present study	6AJG	ZINC15 Database (17M)	canvas clustering (∼17M)200 ns MD simulations (9 and SQ109)MM-GBSA and SwissADMET analysis (4 hits)	4

Previously, Bhakhar et al. utilized an energy-based pharmacophore virtual screening method to screen 175,851 ligands generated from the Asinex BioDesign Library. From this, three potential MmpL3 inhibitors were identified based on their XP docking score in which the detailed 3D interactions and stability of the ligand–receptor complex were analyzed using Biovia Discovery Studio 2020 and 10 ns MD simulations, respectively.^21^ This did not validate the binding affinities via free energy calculations. Also, Chaitra et al. employed a structure-based pharmacophore virtual screening method to screen 1300 ligands gathered from both literature and databases. From here, two potential inhibitors of MmpL3 were identified based on the predicted oral rat LD50 value and from 100 ns MD simulations and Molecular Mechanics Poisson–Boltzmann Surface Area (MM-PBSA) calculations.^22^ Similarly, this study uses the SQ109 bound crystal structure (PDB ID: 6AJG) and glide docking to screen numerous compounds binding to MmpL3. Although in this study, a much more extensive virtual screening workflow (VSW) utilized the ZINC library of ∼17 million compounds along with clustering to group compounds based on their pharmacophore structure including longer MD simulations (2 × 200 ns) and ADMET analysis to evaluate drug-induced protein dynamics, drug safety, drug-likeness profiles, and MM-GBSA analysis to characterize drug–receptor binding energies.

This study utilizes structure-based high-throughput virtual screening (HTVS) followed by MD simulations and MM-GBSA analysis to identify several potential novel inhibitors of MmpL3 from a vast compound library. HTVS is a powerful tool in computer-aided drug discovery^23^ which screens millions of drug-like compounds to a target region to evaluate their binding pose; a few compounds exhibiting better binding poses and docking scores than a reference compound (i.e., SQ109) can be used for further analysis. MD simulations utilize Newton’s laws of motion to provide high-resolution ligand and protein structure dynamics at the atomic level over time. MM-GBSA binding free-energy calculations provide more accurate ligand binding energetics and affinity for a target structure. HTVS of ∼17 million compounds from the ZINC15 drug-like library were docked to the SQ109 binding site of MmpL3 from *M. smegmatis* (PDB ID: 6AJG). From there, nine compounds exhibited higher docking scores compared to SQ109 (Figure S6) and were thus subjected to 2 × 200 ns MD simulations. Four of the nine compounds showed significantly improved binding free energy scores and favorable ADMET properties following an extensive analysis of similar structure clustering and differences in ligand binding poses, structure, and dynamics.

## 2 Methods

**Figure 2:**
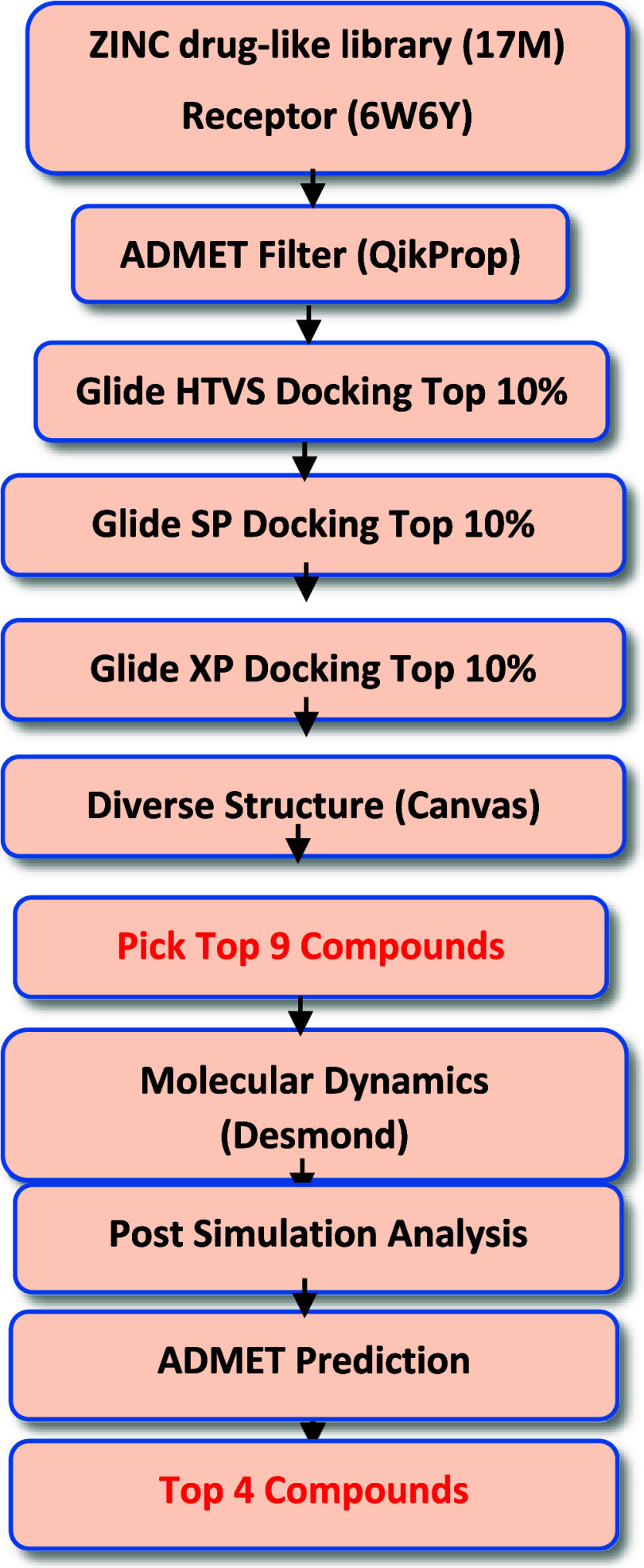
VSW to identify lead inhibitors to the *M. smegmatis* MmpL3 transporter from the ZINC15 drug-like library.

**Figure 3:**
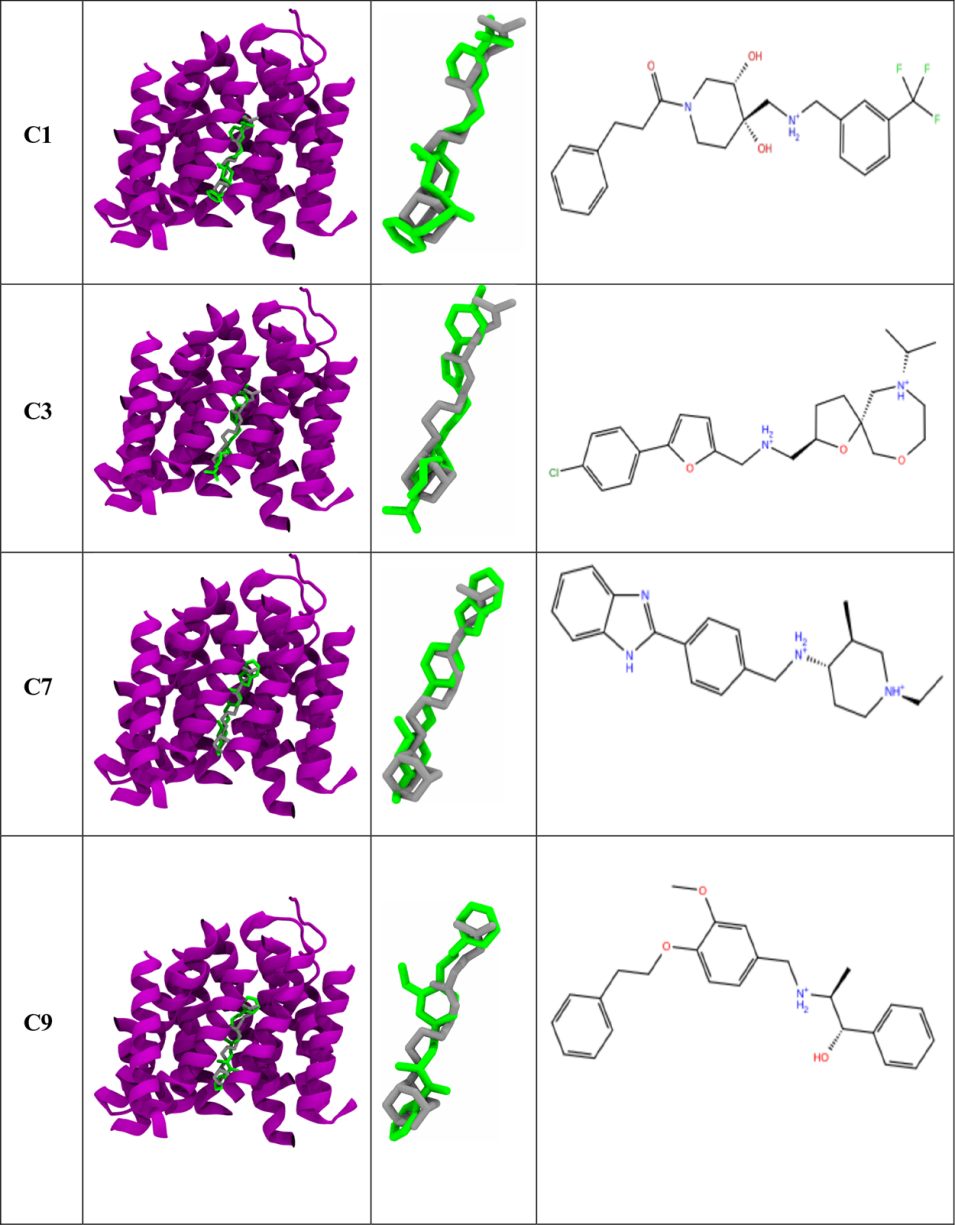
Comparison between the crystal complex of the top four ligands (green) and the crystal ligand (gray). The last column represents the ligand chemical structure. For clarity purposes, only the transmembrane region of the MmpL3 protein (purple) is displayed.

A VSW was developed to identify novel inhibitors to the MmpL3 transporter of *M. tuberculosis* from the ZINC15 compound library, with ∼17 million entries generated in Figure 2. This VSW consisted of 10 essential steps, including the prediction of drug properties, molecular docking, and MD simulations. First, we imported the prepared protein structure from the Protein Data Bank as well as the ZINC drug-like library. Second, compounds were filtered through HTVS and glide extra-precision (XP) docking for accuracy. Following this, ligands that had a lower docking score than the reference compound SQ109 were removed from the list of possible novel inhibitors. The top nine compounds were then chosen by maximizing the number of structure scaffolds. Later steps include MD simulations followed by post-MD simulation analysis, including MM-GBSA binding free energy calculations. Prediction of ADMET properties (absorption, distribution, metabolism, excretion, and toxicity) was used to check the human bioavailability of potential drug candidates. Four compounds with significantly better MM-GBSA binding free energies compared to the reference compound SQ109 were chosen and introduced in the main text (Figure 3).

### 2.1 Preparation of Protein and Ligand Library

The crystal structure of the *M. smegmatis* bacterial MmpL3 protein complex with SQ109 (PDB ID: 6AJG) was imported from the Protein Data Bank. The bacteriophage T4 lysozyme (residues 749 to 929) was removed as it was only necessary for fusing to the C-terminal during protein crystallization to prevent protein degradation.^16^ Homology modeling was also performed by inputting the complete *M. tuberculosis* MmpL3 protein sequence (UniProt: P9WJV5)^18^ using Prime of Schrodinger Suites 2018 to repair any breaks or gaps and to ensure correct sequence order (Figure S4). Bond order correction was done for the SQ109 crystal ligand and empirical p*K*_a_ prediction was calculated at pH 7 from Epik to generate correct ionization states.^24^ The lowest charge state for the crystal ligand was chosen for minimization to relax the atoms for a best-fit structure. The merged protein–ligand complex was then prepared using the Protein Preparation Wizard in Maestro to assign correct bond orders, adding missing hydrogen atoms, creating disulfide bonds, and deleting waters beyond 5 Å from hetero groups. Optimization of the charge state was completed using PROPKA at pH 7. Restrained minimization was then performed to relax the protein using an OPLS3e force field.^25^

### 2.2 Filtering and Docking

A receptor grid file was generated around the original crystal ligand pose using the fully prepared receptor complex. The grid file was generated using a van der Waals scaling factor of 1 and a partial cutoff of 0.25. The prepared crystal ligand was then docked using the generated grid file with no constraints using an OPLS3e force field. Docking scores were calculated using the XP scoring function^25^ to further analyze if the docked ligand pose was improved compared to the original crystal ligand pose (Figure S6). To identify potential lead compounds targeting the MmpL3 receptor, the ZINC15 drug-like library containing ∼17 million compounds was screened via HTVS. The compounds with the top nine glide XP docking scores were chosen for further analysis, along with the crystal ligand (Table 2). The binding poses of the lead compounds were compared to the crystal ligand pose to ensure appropriate parameters (Figure S5). These compounds were considered as viable starting poses and thus acceptable for MD simulations.

**Table 2:** Detailed Information Regarding Various Properties of the Top Nine Compounds and the Crystal Structure from the Glide XP Docking and the ∼200 ns MD Simulations

#	ZINC ID	docking score^c^ (kcal/mol)	VDW^d^ (kcal/mol)	ELE^e^ (kcal/mol)	lipophilic^f^ (kcal/mol)	MM-GBSA^b^ (kcal/mol)	receptor RMSD^b^ (Å)	ligand RMSD^b^ (Å)
ref	*SQ109*	–13.8	–42.9 ± 4.1	–16.3 ± 10.0	–28.1 ± 1.9	–87.3 ± 6.0	2.5 ± 0.1	2.5 ± 0.2
**C1**^a^	**ZINC585283799**	**–15.0**	–52.5 ± 9.1	–11.5 ± 12	–33.0 ± 5.9	–96.9 ± 22	**3.6 ± 0.1**	**4.7 ± 0.3**
C2	ZINC12533192	–14.3	–43.5 ± 9.0	–14.5 ± 4.2	–26.7 ± 5.1	–84.9 ± 16	2.7 ± 0.1	1.9 ± 0.2
**C3**^a^	**ZINC248146645**	**–14.2**	–58.6 ± 11	–0.1 ± 3.9	–36.8 ± 6.7	–95.4 ± 18	**2.3 ± 0.1**	**1.8 ± 0.2**
C4	ZINC585283127	–14.2	–52.5 ± 9.3	–6.8 ± 5.8	–31.6 ± 5.4	–91.0 ± 16	3.0 ± 0.1	1.3 ± 0.3
C5	ZINC14741919	–14.2	–40.5 ± 8.8	–27.4 ± 6.3	–20.8 ± 3.9	–88.8 ± 17	2.3 ± 0.2	2.3 ± 0.2
C6	ZINC19832139	–14.2	–45.9 ± 9.0	–7.06 ± 9.0	–31.2 ± 5.7	–84.2 ± 18	2.7 ± 0.2	2.1 ± 0.6
**C7**^a^	**ZINC221897042**	**–14.0**	–52.6 ± 10	–14.4 ± 5.9	–28.1 ± 5.4	–95.2 ± 19	**1.9 ± 0.1**	**1.6 ± 0.1**
C8	ZINC18223081	–14.0	–58.8 ± 10	5.1 ± 4.4	–31.4 ± 5.3	–85.1 ± 15	3.3 ± 0.1	3.8 ± 0.3
**C9**^a^	**ZINC22107671**	**–13.9**	–60.5 ± 12	–5.7 ± 5.4	–41.9 ± 8.0	–108.2 ± 21	**2.1 ± 0.1**	**1.8 ± 0.2**

a Top four compounds are represented in bold font.

b Based on the snapshots from the last 20 ns simulation.

c Glide XP docking score.

d ΔVDW: change of van der Waals energy (VDW + π–π stacking + self-contact correction) in gas phase upon complex formation.

e Δ*G*BELE: change of electrostatic interactions (GB/generalized born electrostatic solvation energy + ELE/Coulomb energy + hydrogen-bonding) upon complex formation.

f Δ*E*: MM-GBSA binding energy (complex–receptor–ligand).

### 2.3 Ligand Similarity Clustering

Ligand similarity clustering was done using the Canvas program. First, digital fingerprints of 3D ligand structures were generated using a three-point pharmacophore.^26^ Next, hierarchical clustering with default parameters was performed to group similar compounds into different clusters using their digital fingerprints. A cluster ID was then assigned to each compound.^27,28^

### 2.4 MD Simulation

The prepared protein structure was submitted to the OPM 2.0 server^29^ to place the protein in the correct membrane orientation. Each protein–ligand complex of the lead compounds and the docked crystal ligand were prepared separately for MD systems where each complex was surrounded by a POPC (300 K) lipid membrane model.^30^ The system was solvated in an SPC^31^ water box with a buffer distance of 10 Å. A 0.15 M NaCl salt concentration was added, and additional Na+ counterions neutralized the system charge. The systems were built with the OPLS3e^32^ force field via the Desmond System Builder.

#### 2.4.1 MD Relaxation/Minimization

The relaxation and production runs were set up using the Desmond module. Each of the systems was relaxed using the default eight-step relaxation protocol for membrane proteins.^33−35^ First, minimization with restraints on solute-heavy atoms was done. Second, minimization was performed without restraints. Third, the systems were equilibrated using a simulation with a heat transition from 0 to 300 K, a water barrier, and gradual restraining. Fourth, the simulation under the isothermal–isobaric–ensemble (NPT) ensemble (constant pressure and temperature), which included a constant number of particles, pressure of 1 bar, and temperature of 300 K state with a water barrier and restraints on heavy atoms. Fifth, simulations under NPT conditions with additional equilibrations of both lipids and solvents were done. Sixth, simulations under NPT conditions were performed with heavy atoms annealing from 2 to 10 kcal/mol. Seventh, simulations under NPT conditions with Cα atoms were retrained at 2 kcal/mol. Eight, simulations under NPT conditions were done with no restraints for 1.2 ns.

#### 2.4.2 MD Production Run

Twenty separate production runs (two production runs for each system) were performed for each protein–ligand complex under the NPT ensemble by using the default protocol for 200 ns. Using M-SHAKE,^36^ bonds with hydrogen atoms were constrained, allowing a 2.0 fs time-step within the simulations. Long-range electrostatic interactions were analyzed using the k-space Gaussian plot Ewald method,^37^ while using a charge grid spacing of ∼1.0 Å and a direct sum tolerance of 10^–9^. Short-range nonbonding interactions had a cutoff of 10 Å, and long-range van der Waals interactions were based on an approximate uniform density. An r-RESPA integrator^38^ was used to reduce the computation time and calculate nonbonding forces. Short-range forces were updated every 2 fs, and long-range forces were updated every 6 fs. The trajectories were saved every 50 fs for analysis. A pressure of 1 bar was controlled by the Martyna–Tobias–Klein chain coupling scheme^39^ (coupling constant = 2 ps), and the temperature of 300 K was controlled by the Nosé–Hoover^39^ chain coupling scheme (coupling constant = 1 ps).

### 2.5 Convergence of Simulations

**Figure 4:**
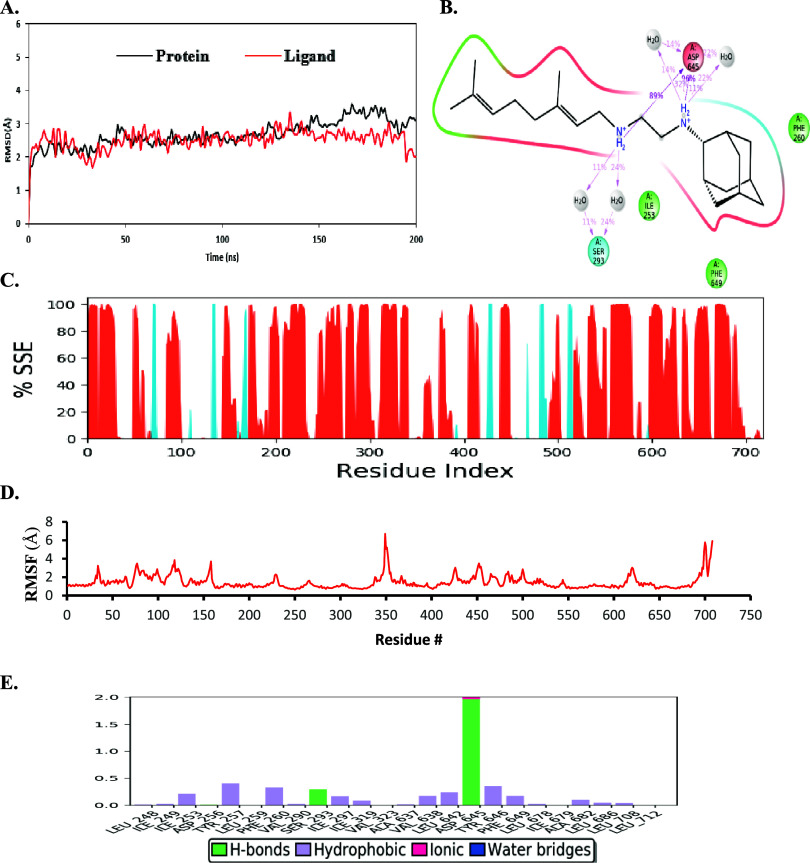
Detailed representation and various properties of simulation interaction diagrams after MD simulations of the crystal structure (*PDB ID:*6AJG). (A) RMSD plot from the MD simulation of ∼200 ns. (B) 2D ligand–protein interaction diagram from the MD trajectory. The residues displayed interacted with the ligand for at least 10% of the simulation time. (C) Protein secondary structure elements (SSE). Orange represents alpha helices, blue represents beta strands, and the white places represent random coil. (D) RMSF graph of protein of the crystal structure docked complex. (E) Protein–ligand contacts during MD simulations. Interaction fraction greater than 1 is because of multiple contacts on one residue.

**Figure 5:**
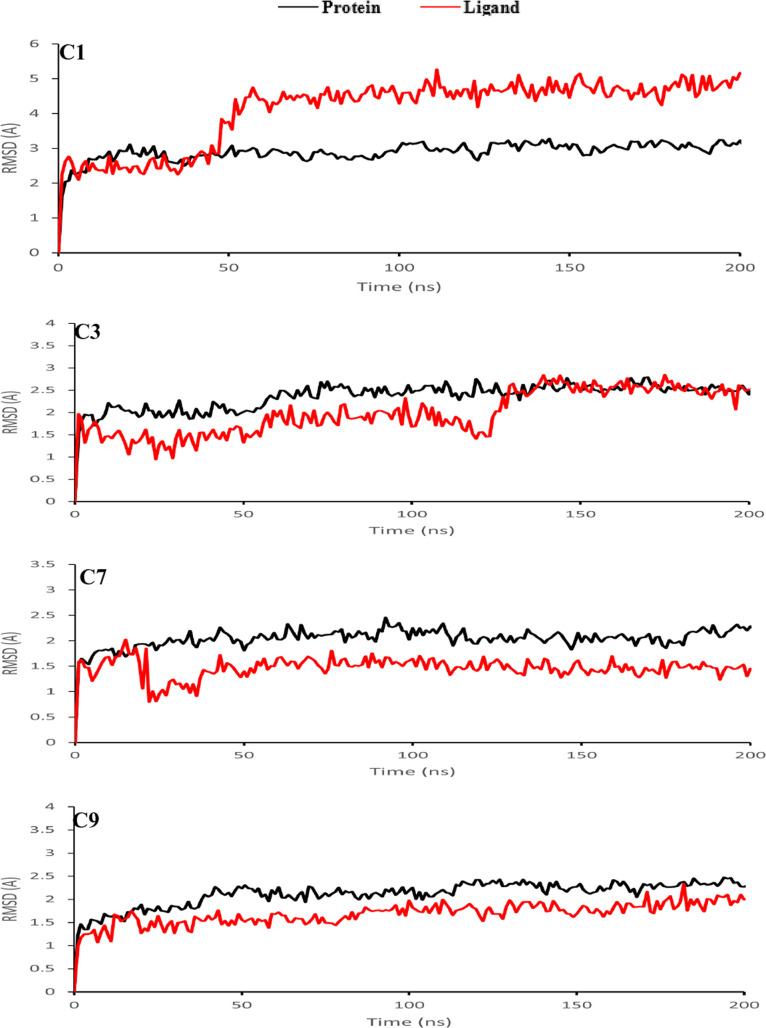
Protein–ligand RMSD of the top four zinc compound ligands during the ∼200 ns MD simulation.

Convergence of the MD simulations was ensured by analyzing the protein Cα and ligand root mean square deviation (RMSD) plots for each trajectory. For each complex, steady-state equilibrium was reached when the plots became relatively flat and stable (Figures 4A and 5), suggesting the simulation time of 200 ns was sufficient to reliably investigate the protein–ligand interactions for the systems.

### 2.6 Postsimulation Analysis

#### 2.6.1 SID Analysis

Using Maestro, the Desmond simulation interaction diagram (SID) tool analyzed the RMSD, root mean square fluctuation (RMSF), SSEs, and 2D-protein–ligand interactions and contacts for the protein receptor and ligand for each complex (Figures 5, 7–9, S12).

#### 2.6.2 Trajectory Clustering Analysis

The Desmond trajectory clustering tool^33−35^ was used to group similar complex MmpL3 receptor structures from the last 20 ns of each MD simulation. The protein backbone RMSD matrix was used as a metric reference for structural similarities, where the clustering method was set to hierarchical clustering with average linkage. A merging distance cutoff was set at 2.5 and the most abundant clusters were used for further analysis. A centroid structure was chosen for each of the most populated clusters (>2% of the total population and is shown in Figure S9). Clustering is an important step to identify the most abundant conformations, which reduces postsimulation analysis complexity.

#### 2.6.3 Binding Energy Calculations and Decompositions

Molecular mechanism-generalized born surface area (MM-GBSA)^40,41^ binding energies were calculated using the complex structures in the last 20 ns of the MD simulations for each system. The MM-GBSA calculation used the OPLS3e force field and the default Prime procedure,^32^ a VSGB 2.0 implicit solvation model.^42^ A slab-shaped region with a low dielectric constant ∼2 was used to simulate the dielectric of the hydrophobic membrane and the other region was assigned with the solvent (water) dielectric constant of ∼80.^25^ This process first minimizes the receptor and ligand geometries independently followed by minimization of the protein–ligand complex. The following equation was used to calculate the binding free energy:
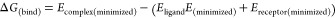


The Coulombic, hydrogen bonds, GB solvation, van der Waals, pi–pi stacking, self-contact, and lipophilic energy terms were merged into three major components where *E*_electrostatic_*, E*_vdW_, and *E*_lipophilic_(*E*_electrostatic_ = *E*_Coulombic_ + *E*_H-bond_ + *E*_GB-solvation_; *E*_vdW_ = *E*_vdW_ + *E*_pi–pi stacking_ + *E*_self-contact_). Moreover, the entropy computation for a system with a membrane of POPC lipids is very complex, so the entropy contribution was ignored in this study. While this may lead to a possible overestimation of the true binding affinity but with the use of a reference ligand through a crystal ligand structure PDB: 6AJG, we may assume that if the entropic properties of the ligand to similar receptors are comparable, the entropy may be canceled out in calculating the binding free energy. Therefore, the MM-GBSA binding free energy difference can be used as a close estimation of relative binding affinity. The top four compounds that exhibited more favorable binding free energies than the reference compound SQ109 were chosen for analysis and discussion from hereon.

##### 2.6.3.1 Free Energy Landscape

RMSD of Ca atoms of two conformational domains of MMPL3, transmembrane domain (TMD) residues 1–34, 170–420, 548–748, and Porter Domain (PD) residues 35–169 and 421–547, were used as Cartesian coordinates to monitor the conformational coupling of TMD and PD (Figure S13). These RMSD values were used to calculate Boltzmannian free energy and defined as lowest energy states (shaded darker) to higher energy states (shaded lighter) (Figure S14).

### 2.7 ADMET Prediction

Prediction of ADMET properties for the top nine best compounds were imported to the SwissADME Web server to predict their physiochemical parameters, ADMET parameter, and pharmacokinetic properties.^43^ The SMILE code for each compound was inserted into the Web server to receive their ADMET properties.

## 3 Results

The top nine compounds from Glide XP docking along with the SQ109 crystal ligand as a reference were subjected to MD simulations. Comparison of the docked ligand poses before and after MD simulation, protein and ligand RMSD calculations, protein–ligand interaction diagrams, SSE plots, and MM-GBSA analysis was done for SQ109 and the top nine compounds. From MM-GBSA analysis, compounds C1 (−96.9 ± 22 kcal/mol), C3 (−95.4 ± 18 kcal/mol), C7 (−95.2 ± 18 kcal/mol), and C9 (−108.2 ± 21 kcal/mol) exhibited the most significantly improved binding free energies compared to SQ109 (−87.3 ± 6.0 kcal/mol) (Table 2). To simplify the discussion, our focus will be directed to compounds C1, C3, C7, and C9 in the main text. Information on the other five compounds (C2, C4–C6, and C8) can be found within the supporting document.

### 3.1 Protein and Ligand RMSD Values of MmpL3, SQ109, and the Top Four Compounds Were Stable from MD Simulations

To determine if each simulation system reached equilibrium, protein receptor RMSD and ligand RMSD were calculated for each protein complex structure (Figures 4, 5 and S10). Receptor and ligand RMSD values averaged over the last 20 ns of each MD simulation have been tabulated (Table 2). For the MmpL3-SQ109 crystal complex, both the protein and ligand RMSDs had converged near the end of the simulation time, indicating that the protein–ligand complex is stable (Figure 4a). Indeed, ligand RMSD values were consistent with protein RMSD values, indicating that SQ109 was likely stable in the receptor binding pocket. Protein and ligand RMSD values of MmpL3 complexed with the top four compounds (C1, C3, C7, and C9) (Figure 5) and all nine compounds (Figure S10) were calculated for both trajectories. Indeed, the protein and ligand RMSD for MmpL3, C1, C3, C7, and C9 complexes showed convergence within the first 50 ns of their simulations and were noticeably smaller than those of the MmpL3-SQ109 complex. For protein RMSD, the averaged values for MmpL3 complexed with compounds C1 (3.6 ± 0.1 Å), C3 (2.3 ± 0.1 Å), C7 (1.9 ± 0.1 Å), and C9 (2.1 ± 0.1 Å) were generally lower than that for MmpL3 complexed with SQ109 (2.5 ± 0.1 Å). For ligand RMSD, the values for compounds C1 (4.7 ± 0.3 Å), C3 (1.8 ± 0.2 Å), C7 (1.6 ± 0.1 Å), and C9 (1.8 ± 0.2 Å) were all generally lower than that of SQ109 (2.5 ± 0.2 Å) (Table 2). Both C1 and C3 displayed higher ligand RMSD values comparable to SQ109 over the simulation between both trajectories, which could be associated with the opening of the central channel and large fluctuations within TM domain of MMPL3 (Figures 5 and S9C,D). Interestingly, C7 and C9 exhibited a significant overlap between receptor and ligand RMSDs over both trajectories, which may likely be due to the interactions with Phe gating residues located toward the cytoplasmic side of the central channel and compounds C7 and C9 (Figures 5 and S9E–G). These results indicate that ligands C1 and C3 may contribute to similar binding properties and characteristics to SQ109 while C7 and C9 may perform possible stabilization within the TM domain of MMPL3. While each trajectory differed by only small RMSD changes, each system remained stable throughout its entire trajectory times.

### 3.2 MD Simulation Shows Improvement in the Binding Pose of the Top Four Ligands

**Figure 6:**
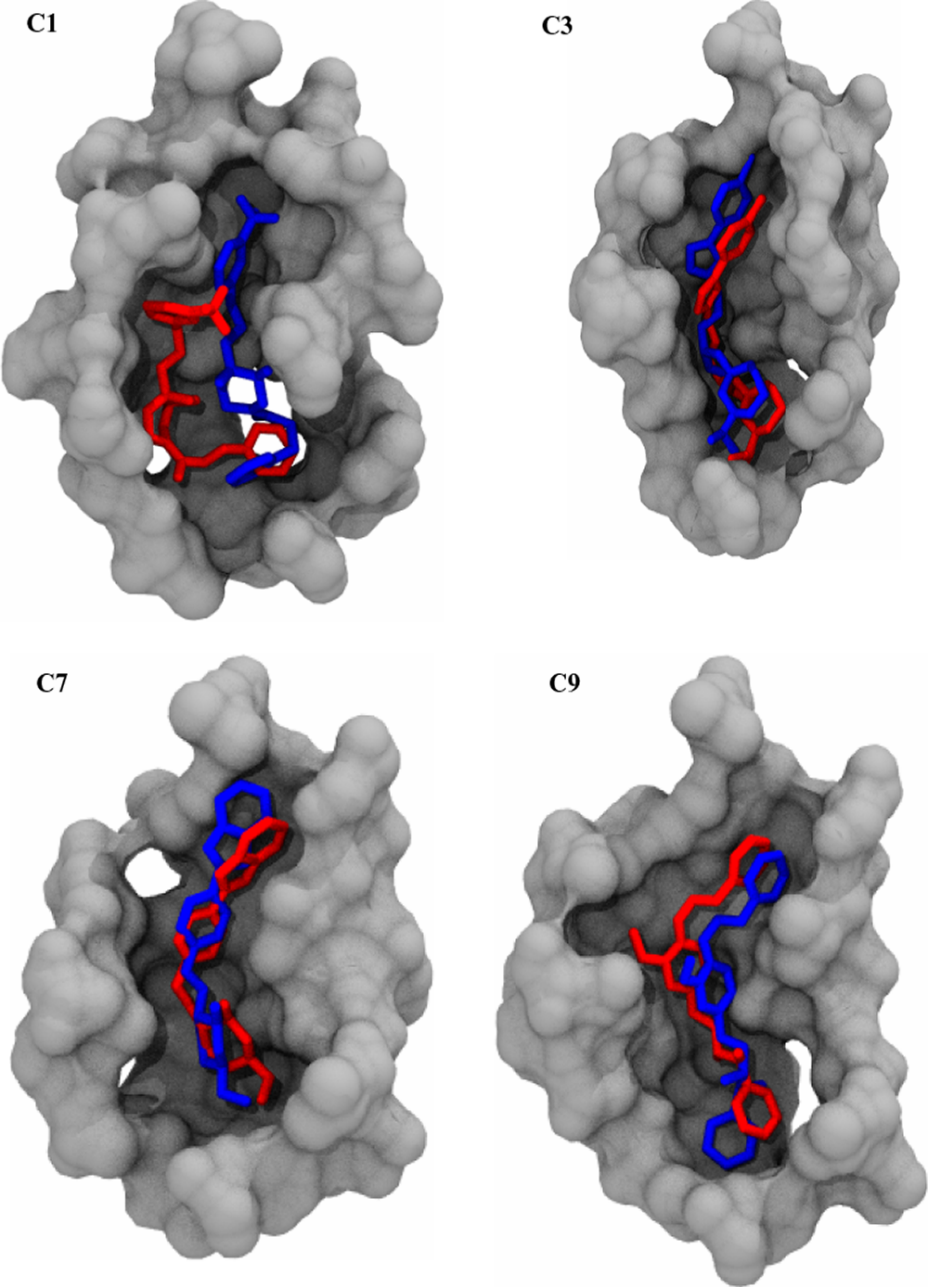
Comparison of ligand XP docking binding poses before (blue) and after (red) MD simulations for the top four lead compounds.

The receptor-binding domain trajectories of the top four ligands were analyzed by comparing their ligand XP docking binding pose before and after MD simulations (Figure 6). Comparison of the docked and MD poses of the top nine compounds were placed in the supporting document (Figure S5). During the simulation, the docked ligand conformation may significantly change to find a more energetically favorable binding pose to optimize interactions with the receptor. The docked and MD poses for SQ109 were very consistent with each other (Figure S2), indicating it was already in an energetically favorable pose. In contrast, compound C1 exhibited the most change in its pose, whereas compounds C3, C7, and C9 exhibited relatively minor changes (Figure 6). This makes sense, as compound C1 exhibited the highest average ligand RMSD value compared to compounds C3, C7, and C9. Overall, these observations indicate that the improved MD poses for the four compounds may be more energetically stable than their Glide XP docked poses.

### 3.3 Critical SQ109–D256–D645 Interactions in MmpL3 Were Conserved in the Top Four Compound Interaction Diagrams

The 2D ligand interaction diagrams from MD simulations can reveal significant interactions occurring within protein–ligand complex (Figure 4B). The protein–ligand interaction histogram provided further insight into the protein–ligand contacts throughout the MD simulation (Figure 4E). Three protein–ligand interactions were observed: hydrogen bonding, hydrophobic interactions, and ionic interactions. SQ109 formed a polar interaction (S293), three hydrophobic interactions (I253, F629, and F260) and a negatively charged electrostatic interaction (D645) with MmpL3. The SQ109–D645 H-bonding interaction was the most prominent, with a frequency interaction percentage value of 96% (Figure 4B). It is believed that SQ109 occupying the interactions between D256-Y646 and Y257-D645 is critical for its inhibitory impact on MmpL3. By fulfilling the ionic interaction to stabilize D256 and D645, the deportation of D256 and D645 causes reinteraction with their respective Y257 and Y646 residues, causing the channel to close. With these interactions held by SQ109 and our ligands, the channel is locked in an open state.^19,44^ Encouragingly, this electrostatic interaction was conserved in the top four compounds with additional unique interactions also observed.

**Figure 7:**
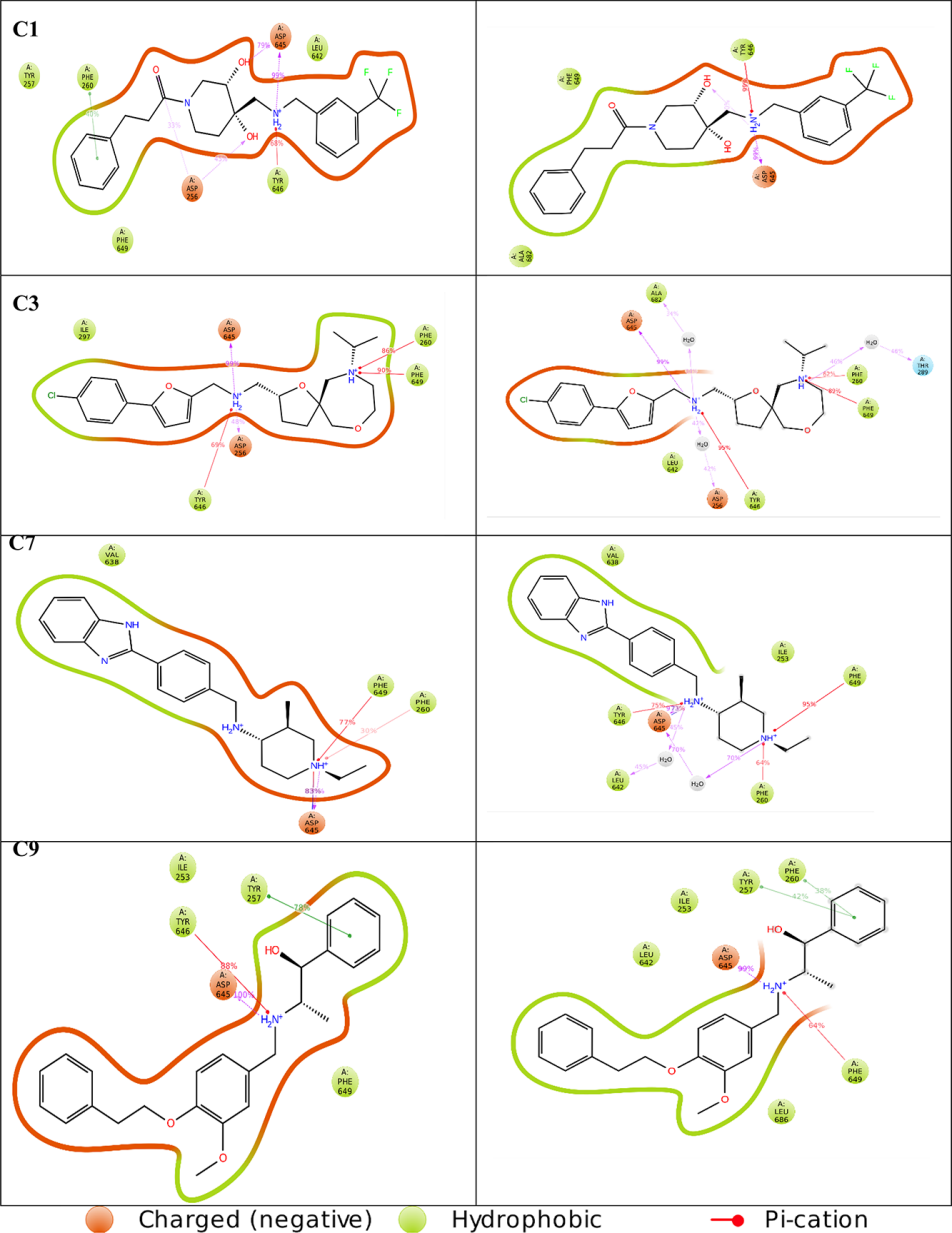
2D ligand interaction diagrams from the MD trajectory for the top four compounds. Residues displayed interactions with the ligand for at least 30% of the simulation.

**Figure 8:**
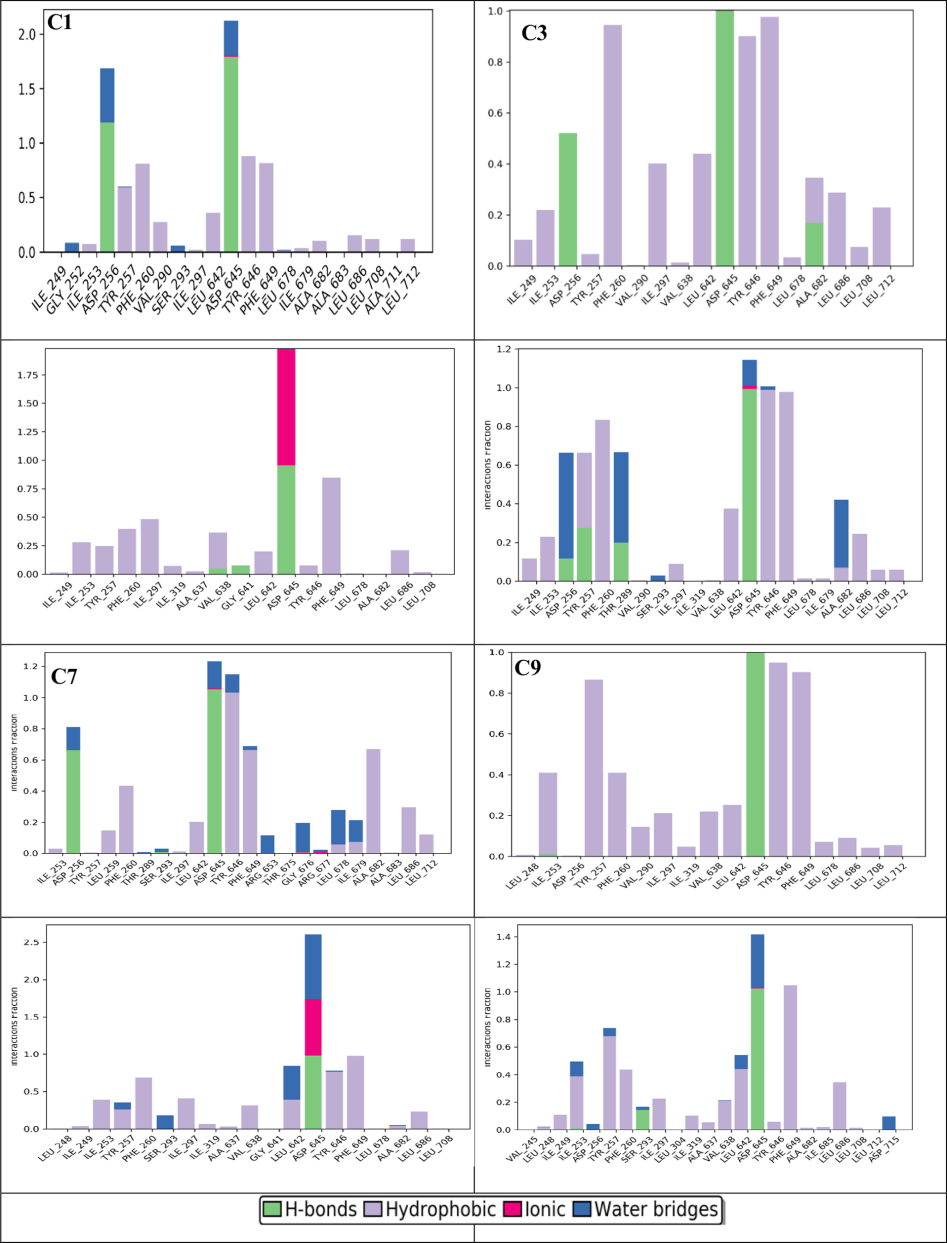
Protein–ligand contacts during MD simulations for the top four compounds. Interaction fraction greater than 1 is possible because of multiple contacts on one residue.

Ligand interaction analysis of compounds C1, C3, C7, and C9 showed slightly higher interaction fractions compared to SQ109, with compounds C3 and C9 having the most hydrophobic interactions overall. A protein–ligand interaction 2D diagram for the top four compounds (Figure 7) and their corresponding interaction histograms (Figure 8) have been provided. Histograms displaying the protein–ligand interactions for the top nine compounds can be found in the supporting document (Figure S11). Compounds C1, C3, C7, and C9 exhibited negatively charged electrostatic and hydrophobic interactions such as SQ109, as well as additional polar interactions with residue S293. Unique interactions were also present in the top four compounds. Compound C1 formed hydrogen bonding interactions with D256, pi–pi stacking interactions with F260, pi–cation interactions with Y646, and hydrophobic interactions with D257. Compound C3 displayed hydrogen bond interactions at D256 and A682 and pi–cation interactions with F260, F649, and Y646. Compound C7 exhibited pi–cation interactions between F260 and F649. Interestingly, a salt bridge formed between D645 and compound C7; salt bridges are among the strongest of all noncovalent interactions, indicating this may be the most important interaction exhibited by compound C7. Finally, compound C9 formed hydrogen bonding with D645 and I253, pi–cation interactions with Y646, and pi–pi stacking with Y257. There is a direct correlation between the key residues mentioned and the significant differences in the MM-GBSA binding energies among SQ109 and the four leading compounds. In fact, ΔLIPO shows that out of the top four compounds, C3 and C9 form stronger hydrophobic interaction with ΔLIPO values of −36.8 ± 6.7 and −41.9 ± 8.0 kcal/mol, respectively (Table 2).

Overall, four protein–ligand interactions were observed: hydrogen bonding, hydrophobic interaction, ionic bonding, and water-bridge formation. To visualize the frequencies at which these specific interactions occur, it is useful to refer to the 2D protein–ligand interaction histogram plots, provided by the SID analysis (Figure 8). Water bridges are a unique interaction, which is seen only in compound C1, while hydrogen bonds and hydrophobic forces dominate the interactions between MmpL3 and SQ109, C3, C7, and C9. Additionally, compound C7 exhibited a unique ionic interaction at D645. Overall, each compound displayed interactions believed to be crucial in MmpL3′s transport mechanism.

### 3.4 MmpL3 and the Top Four Compounds Exhibited Lower Protein and Ligand Fluctuations Than with SQ109

Protein SSE plots, clustering analysis, and RMSF analysis were used to characterize protein and ligand structure fluctuation dynamics. SSE calculates the distribution of secondary structures (alpha helices, beta-strands, and random coils) by residue index throughout the protein structure (Figure 4C). MmpL3 alpha helical content of ∼48.36% and a beta-strand content of ∼4.49% was observed, which equated to a total SSE percentage of 52.85%. When complexed with compounds C1, C3, C7, and C9, MmpL3 SSE did not noticeably change, indicating no loss of protein secondary structural elements (Figure S12).

As expected, the most abundant conformation of the MmpL3-SQ109 MD crystal structure exhibited slight differences with the original crystal structure, notably within transmembrane helices that make up the proton channel of MmpL3, TM4, TM5, TM6, TM10, TM11, and TM12 (Figure S9A). The cytoplasmic view shows that these helices shifted slightly away from the transmembrane region in the most abundant cluster compared with the crystal structure. The ligand conformations were mostly consistent, with slight differences likely contributing to the helical shifts in channel lining TM helices (Figure S9B–G). SQ109 disrupts two Asp-Tyr pair interactions (D256-Y646 and D645-Y257) on TM4 and TM10 and shifts them away from each other by about 2–4 Å, respectively (Figure S9A). The tips of the phenyl rings in F260 and F649 shift downward by about 7 Å to make space for SQ109 binding. The general consistency in the MmpL3 protein and SQ109 ligand conformations indicated that the simulations accurately reproduced the original crystal structure conformation. Interestingly, the conformations of the top four compounds were quite similar to that of SQ109, suggesting similar protein–ligand interactions.

**Figure 9:**
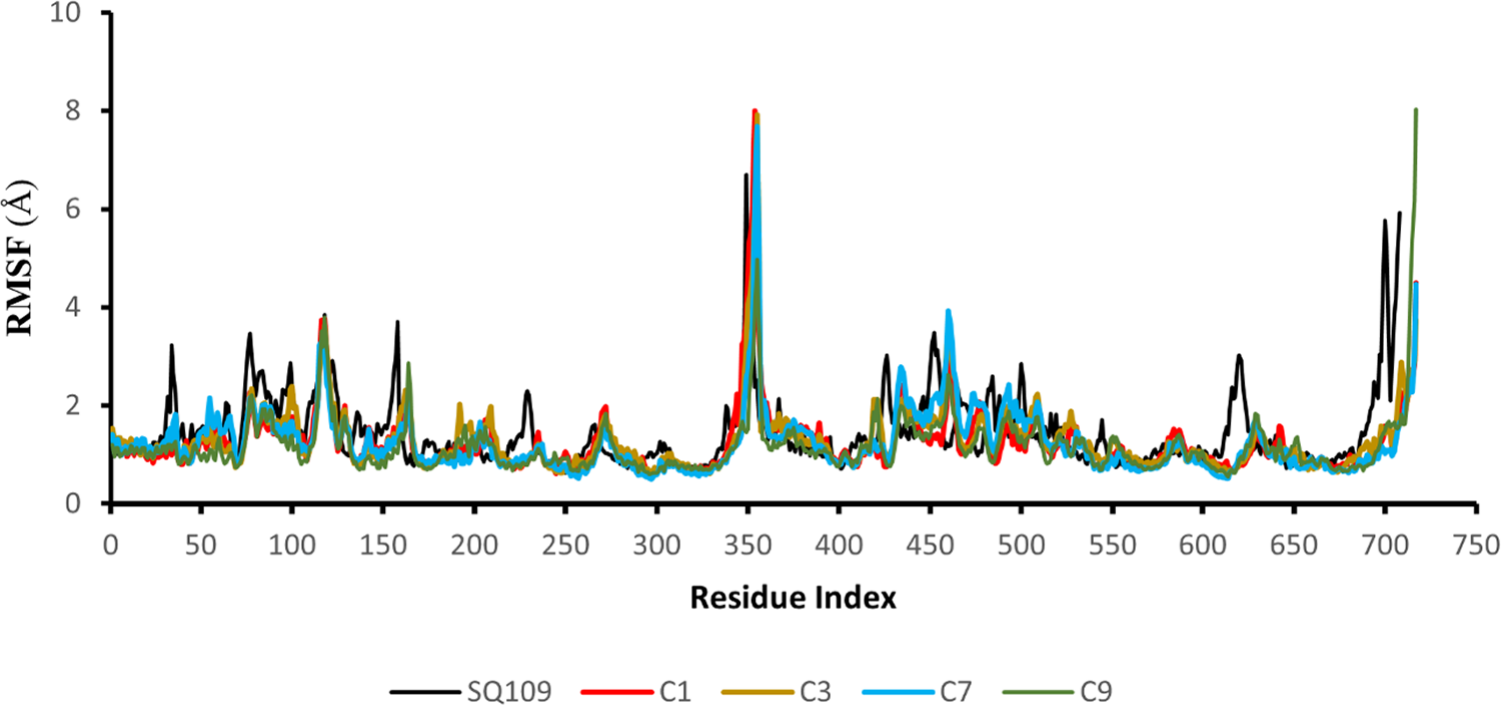
Protein Cα RMSF (Å) for the top four compounds, including the crystal structure during the MD simulation.

The receptor RMSF plot of MmpL3 with SQ109 and the top four compounds was generated (Figure 9). As residues 345–388 were not present in the crystal structure, their protein-Cα RMSF values are not available and show a large peak in RMSD. The general trend expected from these plots was observed; rigid components of the receptor (i.e., TM helices) exhibited lower RMSF values (lower flexibility), while the N- and C-termini displayed higher RMSF values (higher flexibility). For the crystal structure and the four compounds, protein RMSF was generally low with some spikes observed (residue indices: ∼120, ∼360, ∼∼380, and ∼490) (Figure 9). Receptor RMSF values of the four leading compounds were generally lower across each residue compared to the crystal ligand SQ109 in which compound C9 exhibited the lowest fluctuations. Namely, residues 400–600 exhibited increased RMSF by SQ109 (2–6 Å), whereas compound C9 had minimal fluctuations (<2 Å) throughout these residues. These decreased RMSF values indicate increased protein complex stability with compounds C1, C3, C7, and C9 compared with SQ109, as supported by MM-GBSA binding free-energy calculations (Table 2).

**Figure 10:**
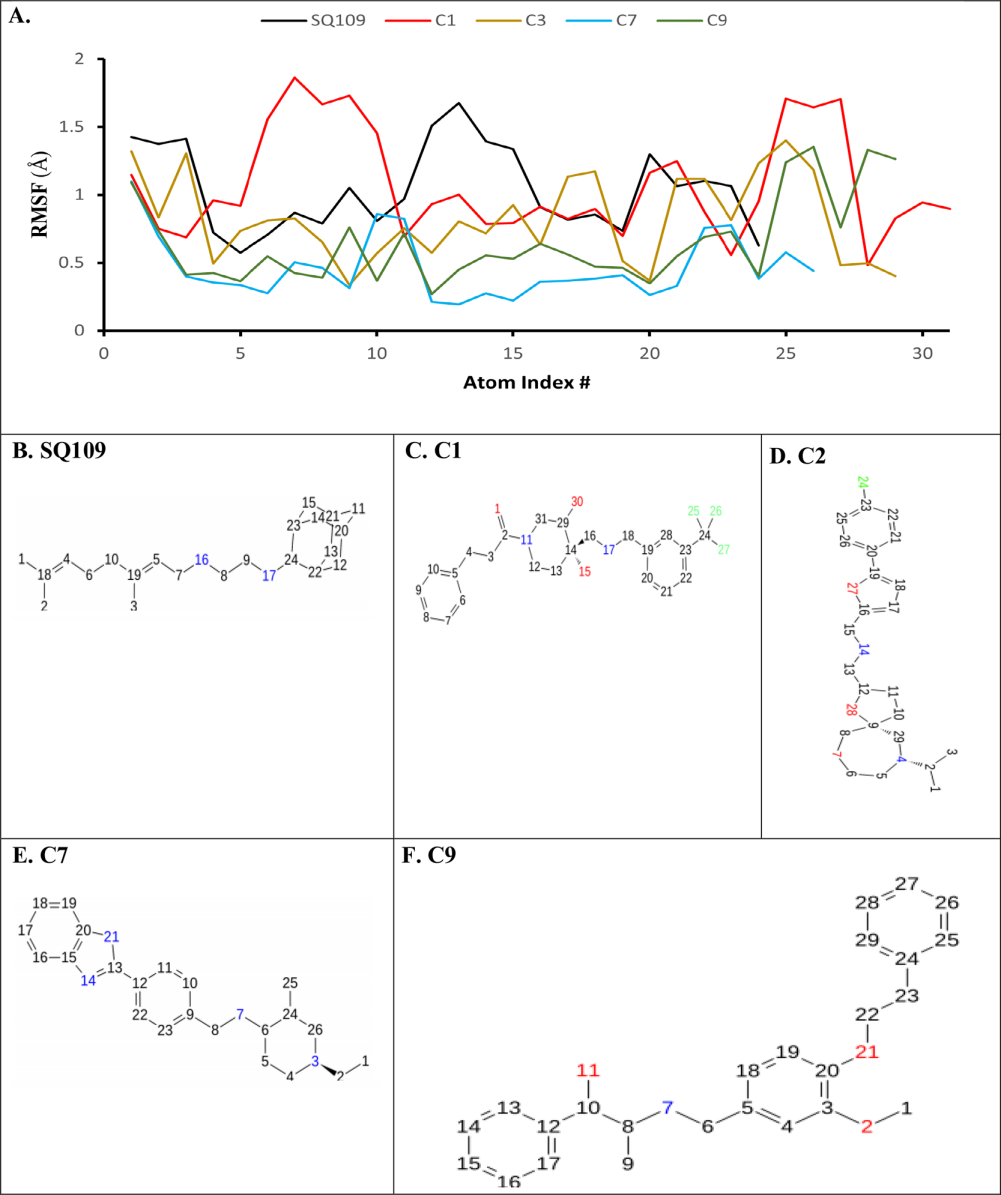
Ligand root mean square fluctuations (L-RMSF) of SQ109 and the four leading compounds.

The ligand RMSF plot examines the flexibility of the ligand within the binding pocket (Figure 10). The four leading compounds expectedly displayed different conformation patterns compared to SQ109 due to their novel scaffolding; thus, their ligand RMSF values should also differ. Indeed, ligand RMSF values were generally lower across each atom index compared with SQ109. Additionally, the average ligand RMSF values of compounds C1 (1.31 Å), C3 (0.93 Å), C7 (0.77 Å), and C9 (0.86 Å) were lower compared to SQ109 (∼1.53 Å). Compounds C7 and C9 exhibited the lowest ligand RMSF, whereas compound C1 exhibited the highest ligand RMSF. To SQ109, compound C1 exhibited relatively higher fluctuations (atoms 5–10 and 21–27) and lower fluctuations (atoms 11–20) (Figure 10A). Ligand RMSF values were comparatively lower in compounds C3, C7 and C9. The increased number of rotatable bonds within compound C1 (10 total) compared to compounds C3, C7, and C9 likely increases its molecular flexibility and its ligand RMSF (Figure S13). Overall, the lower ligand RMSF values of the top four compounds suggest they are potentially stronger inhibitors of MmpL3 than SQ109.

### 3.5 Nonpolar and Lipophilic MM-GBSA Terms Enhance Binding Affinities of the Top Four Compounds

As previously stated, MM-GBSA analysis aided in selection of compounds C1, C3, C7, and C9 for further analysis, which showed significantly improved binding free energies than SQ109 to MmpL3 (Table 2). We noted that the van der Waals (VDW, weak nonpolar interactions) and lipophilic (LIPO) terms were generally lower for compounds C1, C3, C7, and C9 ranging from (VDW: −52.5 ± 9.1 to 6.5 ± 12 kcal/mol; LIPO: −28.1 ± 5.4 to 41.9 ± 8.0 kcal/mol) compared to those of SQ109 (VDW: −42.9 ± 4.1 kcal/mol; LIPO: −28.1 ± 1.9 kcal/mol). In contrast, the electrostatic term (ELE, electrostatic, Coulombic, and hydrogen-bonding interactions) did not show an obvious pattern. The increased hydrophobic and nonpolar interactions are likely explained by the increased presence of aromatic groups (primarily benzene) in all four compounds. The fluctuating electrostatic hydrogen-bonding interactions are likely explained by the differences in polarity of each compound. For instance, the higher ELE of C1 (−11.5 ± 8.1 kcal/mol) is likely due to the hydroxyl (OH) and fluorine (F) groups, as well as the charged secondary amine (NH_2_^+^). Compounds C3, C7, and C9 only share the (NH_2_^+^) group with C1; however, they have markedly fewer hydrogen-donor groups, which might explain their lower ELE terms.

#### 3.5.1 Free Energy Landscape Shows Conformational Coupling Characteristics

Free energy landscape for each system was conducted to identify conformational coupling seen in MMPL3′s two structural domains (TMD) and (PD) (Figure S14). The lowest energy states defined by the coupling of these domains have been tested through this method in our previous study, showing the inverse correlation of TMD and PD seen through RMSD increases in one and decrease in the other. In this study, the combined trajectories (400 ns) show similar characteristics to SQ109 causing primarily high RMSD changes in PD where the TMD shows very low RMSD changes in the lowest energy states throughout the trajectory. SQ109 shows peaks lowest energy states in PD from 1.8 to 2.9 Å and 0.7–1.2 Å in TMD. Similarly, each trajectory of our top for compounds C1, C3, C7, and C9 showed comparable RMSD to that of the TMD to SQ109 with all ranges being less than 2 Å. We have shown previously that small RMSD changes within the TMD can cause large changes in PD^19^. C3 showed the most similar states to SQ109 with the lowest free energy states between 1.6 and 2.2 Å in PD and 0.8 to 1.6 Å in TMD. Notably, C7 presented the largest state changes in PD 2.2–3.4 Å and the lowest RMSD in TMD 0.4–0.8 and 1.0–1.2 Å likely due to inherent stability and binding to core Asp-Tyr residues. C1 and C9 were very similar lowest energy states, 1.4–2.0 Å in PD and 0.7–1.4 Å in TMD. These four systems present lower RMSD values associated with their free energy states, causing potentially better inhibition than SQ109.

### 3.6 Predicted ADMET Properties Exhibited Good Human Oral Bioavailability for the Four Leading Compounds

To assess compound pharmacokinetic properties and bioavailability, ADMET properties were predicted for SQ109, compounds C1, C3, C7, and C9, and other known MmpL3 inhibitors (Table 3). ADMET properties for the top nine compounds were placed in the Supporting Information (Table S1). Compounds C1, C3, C7, and C9 and known MmpL3 inhibitors (SQ109, NITD-349, PIPD1, and C215) exhibited high gastrointestinal (GI) absorption. However, the top four compounds were predicted to inhibit at least one cytochrome P450 enzyme (CYP), including CYP1A2, CYP2C19, CYP2D6, and CYP3A4. Out of the CYPs, CYP3A4 possesses the highest activity in the small intestine and liver and metabolizes 50% of administered drugs.^45^ CYP inhibitors may increase the plasma concentration of other coadministered drugs if they are normally metabolized by CYP3A4; this would lead to increased circulation time and might cause undesirable side effects. Compounds C3, C7, and C9 are predicted to inhibit CYP3A4, thus caution is advisible for patients taking other medications. Another important ADMET property is blood–brain barrier (BBB) permeability, where the BBB functions to protect the brain from exposure to neurotoxic molecules. The top four compounds, SQ109 and the other known MmpL3 inhibitors, were predicted to permeate the BBB, potentially leading to harmful side effects. The top four compounds fulfilled Lipinski’s rules of drug-likeness. Lastly, the PAINS (pan-assay interference compounds) and Brenk structural alert system gave off zero alerts to all four compounds, indicating a low chance of false positives from occurring in the assays and stable chemical properties, respectively. Out of the four top compounds, compound C1 shared the most similar drug properties to those of crystal ligand SQ109. Additional predicted ADMET properties of the top nine compounds, along with SQ109 and other known MmpL3 inhibitors, can be found in Figure S15.

**Table 3:** Predicted Pharmacokinetics ADMET Properties for the Top Four Compounds, Crystal Structure Reference, and Other Known MmpL3 Inhibitors by the SwissADME Server

compound	GI absorption	BBB permeant	CYP1A2 inhibitor	CYP2C19 inhibitor	CYP2C9 inhibitor	CYP2D6 inhibitor	CYP3A4 inhibitor	Lipinski Rule	PAINS	Brenk
SQ109	high	yes	no	no	no	yes	no	1	0	1
NITD-349	high	yes	yes	no	no	yes	no	0	0	0
PIPD1	high	yes	yes	yes	no	yes	no	1	0	0
C215	high	yes	yes	yes	yes	yes	yes	0	1	0
C1(ZINC585283799)	high	yes	no	no	no	yes	no	0	0	0
C3(ZINC248146645)	high	yes	no	no	no	yes	yes	0	0	0
C7(ZINC221897042)	high	yes	yes	yes	no	yes	yes	0	0	0
C9(ZINC22107671)	high	yes	no	yes	no	yes	yes	0	0	0

## 4 Discussion

MmpL3 is the only known RND transporter to function in a monomeric state in transporting TMM substrate to the periplasm by a PMF-dependent mechanism, where it is synthesized and incorporated in the *Mtb* cell wall. This process is driven by the influx of protons (i.e., H^+^ or H_3_O^+^) from the periplasm through MmpL3’s transmembrane channel and into the cytoplasm generating the PMF. The detailed process of MmpL3-mediated TMM transport can be found in our previous paper.^19^ The promising inhibitor SQ109 hinders proton translocation and eliminates deprotonation capabilities for central D256 and D645 thus halting TMM translocation. From MD simulations of the SQ109–MmpL3 complex, conformational changes in the transmembrane domain narrowed the initial binding site of TMM (i.e., region between MmpL3 TM helices 7 and 8), likely making TMM translocation more difficult.^19^

Structural comparison between C1, C3, C7, and C9 to SQ109 and other MmpL3 inhibitors revealed both similar and novel scaffolding (Figures S7 and S9). Encouragingly, compounds that exerted biological potency against MmpL3 were structurally similar to C1, C3, C7, and C9 (Table 2), suggesting they may also be biologically active. Furthermore, a SciFinder search revealed no known activity studies reported on these compounds; therefore, these are novel for targeting Mmpl3.

As previously discussed, the two Asp-Tyr residue pairs (D256 and Y646, D645 and Y257) play a vital role in the movement of the PMF. Clustering of the most abundant structures of each complex displays movement of these residues, and further insight into this disruption can be interpreted from the MM-GBSA decomposition, which shows some significantly higher binding energies in the lead compounds to the receptor compared to SQ109 (Table 4). This is further supported by the display of tight binding shown in the protein and ligand RMSF. The ligand interaction diagrams indicate most of the interactions occurring between SQ109 and the receptor are conserved among the lead compounds with additional unique interactions, specifically the Asp-Tyr residue pairs and the key phenylalanine residues.

**Table 4:** Protein–Ligand Contacts during MD Simulations for the Top Four Compounds^a^

SQ109	C1(ZINC585283799)	C3(ZINC248146645)	C7(ZINC221897042)	C9(ZINC22107671)
L248^0.01^				L248^0.01^
I249^0.03^	I249^0.01^	I249^0.10^	I249^0.02^	
	G252^0.08^			
I253^0.22^	I253^0.08^	I253^0.22^	I253^0.27^	I253^0.41^
	D256^1.68^	D256^0.52^		D256^0.004^
Y257^0.24^	Y257^0.60^	Y257^0.05^	Y257^0.25^	Y257^0.87^
F260^0.24^	F260^0.81^	F260^0.95^	F260^0.40^	F260^0.41^
V290^0.01^	V290^0.28^	V290^0.01^		V290^0.15^
F292^0.08^				
S293^0.18^	S293^0.06^			
I296^0.02^				
I297^0.18^	I297^0.02^	I297^0.40^	I297^0.48^	I297^0.21^
I319^0.05^			I319^0.07^	I319^0.05^
A582^0.04^				
A637^0.01^			A637^0.03^	
V638^0.11^		V638^0.01^	V638^0.37^	V638^0.22^
			G641^0.08^	
L642^0.18^	L642^0.36^	L642^0.44^	L642^0.20^	L642^0.25^
D645^2.03^	D645^2.12^	D645^1.00^	D645^1.98^	D645^1.00^
Y646^0.23^	Y646^0.88^	Y646^0.90^	Y646^0.08^	Y646^0.95^
V648^0.01^				
F649^0.23^	F649^0.82^	F649^0.98^	F649^0.85^	F649^0.90^
L678^0.01^	L678^0.01^	L678^0.03^	L678^0.01^	L678^0.07^
I679^0.01^	I679^0.34^			
A682^0.10^	A682^0.10^	A682^0.35^	A682^0.01^	
	A683^0.01^			
L686^0.05^	L686^0.15^	L686^0.29^	L686^0.21^	L686^0.07^
L708^0.03^	L708^0.12^	L708^0.08^	L708^0.02^	L708^0.04^
	L711^0.01^			
	L712^0.12^	L712^0.23^		L712^0.05^

a Fraction of contacts are annotated by the superscript following each residue listed.

## 5 Conclusions

Ultimately, this study may assist in the further investigation and testing of novel inhibitors of the MmpL3 transporter of *Mtb*. We performed a thorough investigation of a total of 17 million ZINC15 compounds using an HTVS method which provided the most potential hits. MD simulations further validated the top hits. In this study, nine compounds were selected, from which four potential inhibitors of *Mtb* showed commendable docking scores ranging from −13.9 to −15.0 kcal/mol. The VSW provided the best top four hits overall, which exhibited good binding affinity toward the active site. Based on the XP glide docking, binding affinity, and ADMET properties, the top four ZINC15 compounds are potentially promising inhibitors of the MmpL3 transporter of *Mtb*. Overall, these findings may aid in the design of better inhibitors to the MmpL3 transporter for the treatment of emerging drug-resistant *Mtb* strains.

## Supporting Information Available

Predicted ADMET Properties of top nine compounds; structures of anti-TB agents; comparison between the docked and crystal binding pose of SQ109 to MmpL3; structures of known MmpL3 inhibitors; MmpL3 protein sequence comparing between *M. smegmatis* and *M. tuberculosis*; comparison of ligand XP docking binding pose before and after MD simulation of top 9 ligands; table represents Zinc ID, structure, SMILE code, docking score, and cluster IDs of the top nine zinc compounds and the reference compound; ligand comparison between C1 & PIPD1, C5 & C215, C7 & C215, and C7 & NITD-349; structure alignment of crystal ligand and most abundant structures of SQ109 and top 4 ligands; protein ligand RMSD of top nine compounds of two ∼200 ns MD simulation; protein–ligand contacts during MD simulations for the crystal ligand SQ109 and nine leading compounds; protein Secondary structure elements (SSE) for MmpL3 complexed with SQ109 and the top nine compounds; ligand torsion plot summarizing the conformational progression of the rotatable bonds of SQ109 and the top four ligands throughout the two MmpL3 simulation trajectories; free energy landscape (unit: kcal/mol) of the 2 trajectories (400 ns) of each of SQ109 and our 4 highlighted systems of MMPL3; pedicted ADMET properties for the top nine best compounds including the reference compound SQ109 and other known inhibitors of MmpL3 obtained from the SwissADME server (PDF)
