# Supplementary material for: Novel Inhibitors to MmpL3 Transporter of Mycobacterium tuberculosis by Structure-Based High-Throughput Virtual Screening and Molecular Dynamics Simulations
Source: ACS Omega. 2024 Mar 12;9(12):13782–96. doi: 10.1021/acsomega.3c08401 (PMC10976370; doi:10.1021/acsomega.3c08401)
Supplement: Supplementary file 1 — ao3c08401_si_001.pdf [file ao3c08401_si_001.pdf]

Novel Inhibitors to MmpL3 Transporter of *Mycobacterium tuberculosis* by Structure-based High Throughput Virtual Screening and Molecular Dynamics Simulations

Hetanshi Choksi[1], Justin Carbone[1], Nicholas J. Paradis, Lucas Bennett, Candice Bui-Linh and Chun Wu[*]

Department of Molecular & Cellular Biosciences, College of Science and Mathematics, Rowan University, Glassboro, New Jersey 08028, United States.

[1] these authors contribute equally

*To whom correspondence should be addressed: wuc@rowan.edu

Table S1. The predicted ADMET properties for the top nine best compounds including the reference compound from the SwissADME server.

Compound	GI absorption	BBB permeant	CYP1A2 inhibitor	CYP2C19 inhibitor	CYP2C9 inhibitor	CYP2D6 inhibitor	CYP3A4 inhibitor	Lipinski Rule	PAINS	Brenk
Crystal Structure (PDBID: 6AJG)	High	Yes	No	No	No	Yes	No	1	0	1
ZINC585283799	High	Yes	No	No	No	Yes	No	0	0	0
ZINC12533192	High	Yes	Yes	No	No	Yes	No	0	0	0
ZINC248146645	High	Yes	No	No	No	Yes	Yes	0	0	0
ZINC585283127	High	Yes	No	No	No	Yes	Yes	0	0	0
ZINC14741919	High	No	No	No	No	No	No	0	0	0
ZINC19832139	High	Yes	Yes	Yes	No	Yes	No	1	0	0
ZINC221897042	High	Yes	Yes	Yes	No	Yes	Yes	0	0	0
ZINC18223081	High	Yes	Yes	Yes	Yes	Yes	Yes	0	0	1
ZINC22107671	High	Yes	No	Yes	No	Yes	Yes	0	0	0

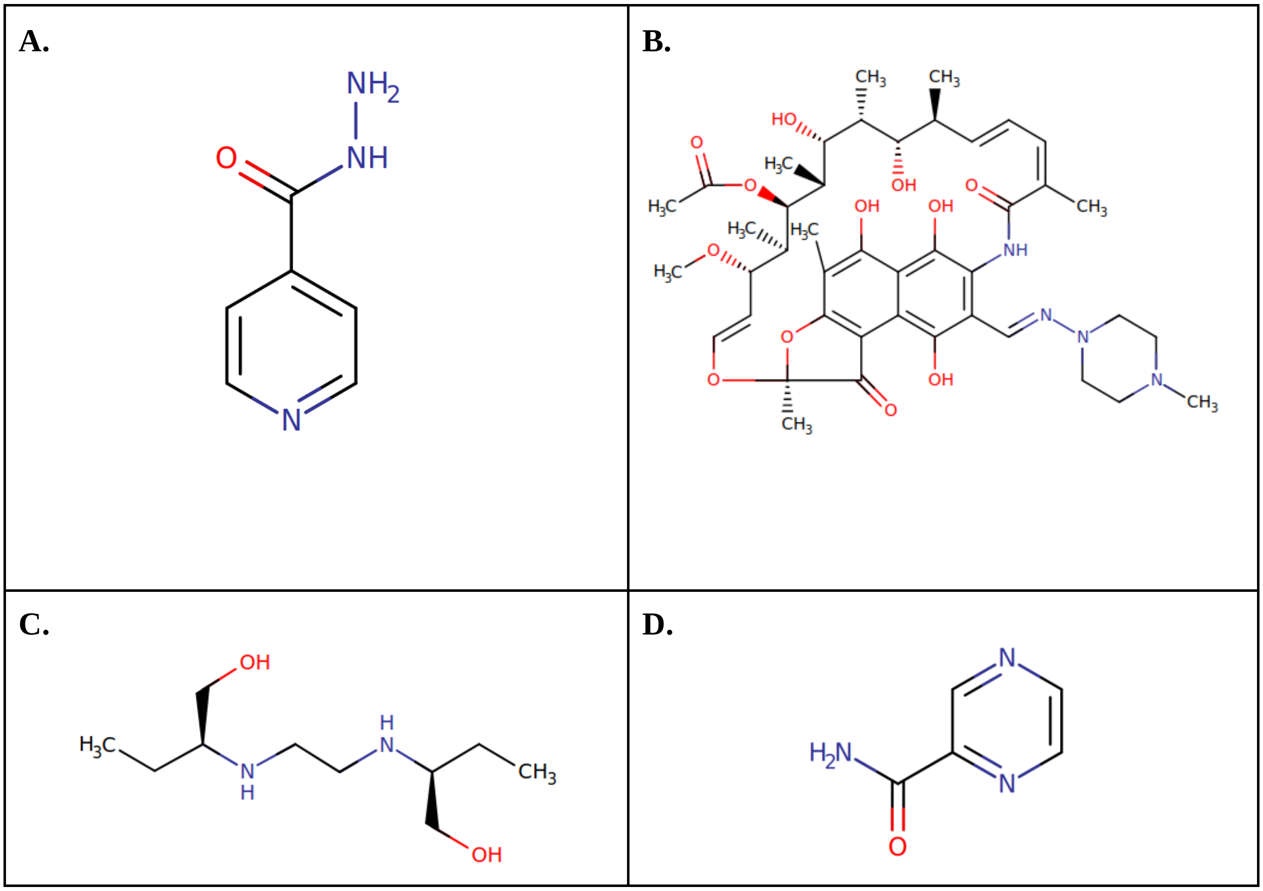

Figure S1. Structures of Anti-TB agents **(A)** isoniazid, **(B)** rifampicin, **(C)** ethambutol and **(D)** pyrazinamide.

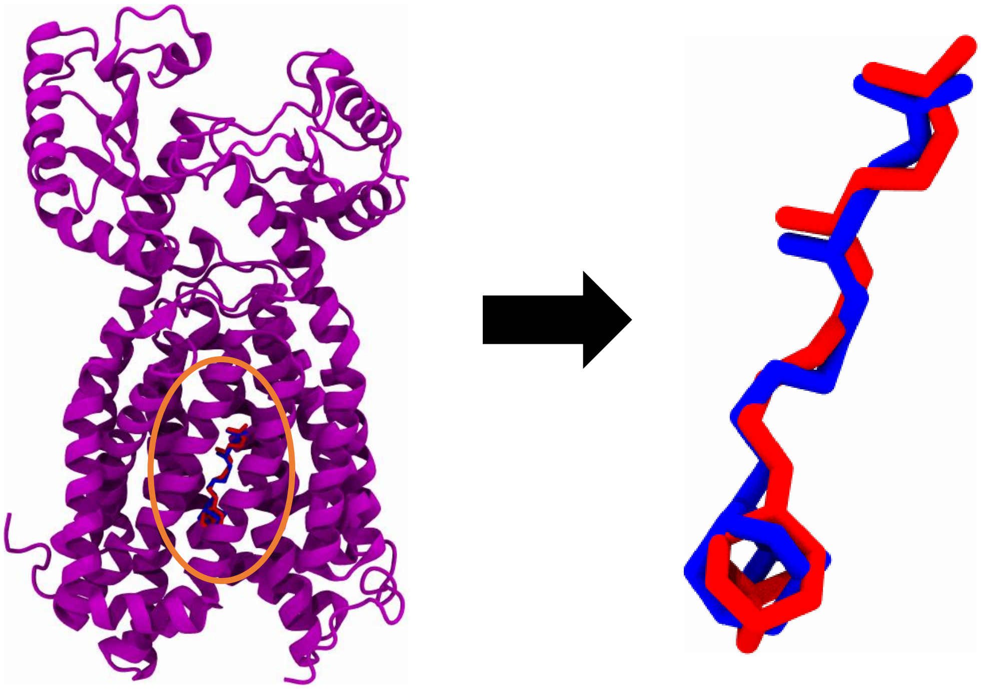

Figure S2. Comparison between the docked pose (red) and the crystal binding pose (blue) of SQ109 to MmpL3 (PDB ID: 6AJG).

Compound	Structure	MIC (μM)
**SQ109**	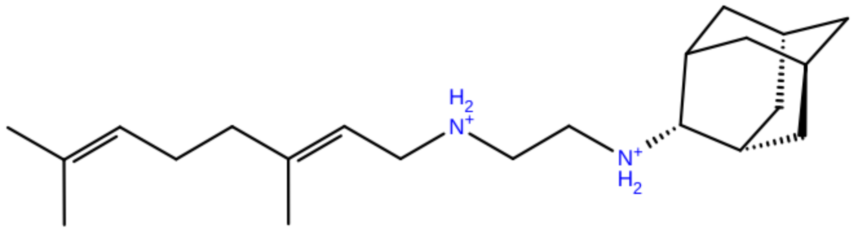	0.78
**NITD-349**	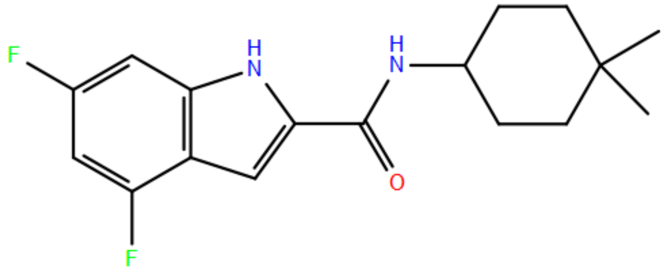	0.023
**PIPD1**	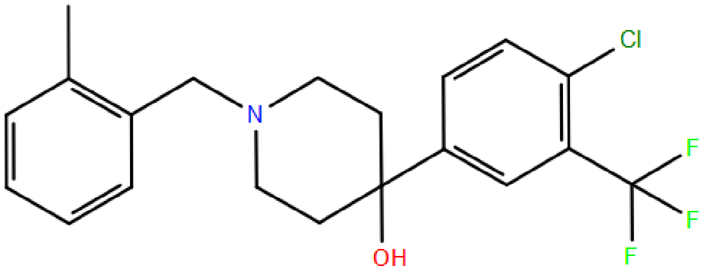	1.28
**C215**	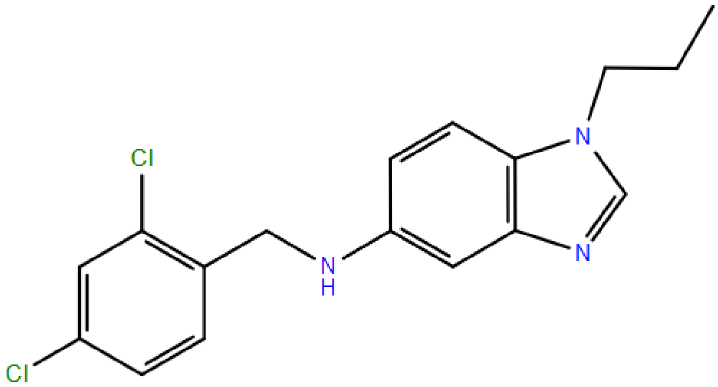	16.0

Figure S3. Structures of known MmpL3 inhibitors, SQ109, PIPD1, C215, and NITD-349 and their minimum inhibitory concentration (MIC) values.

*Degiacomi et al., (2020), Promiscuous Targets for Antitubercular Drug Discovery: The Paradigm of DprE1 and MmpL3, Applied Sciences-Basel, (10)2: 1-19.

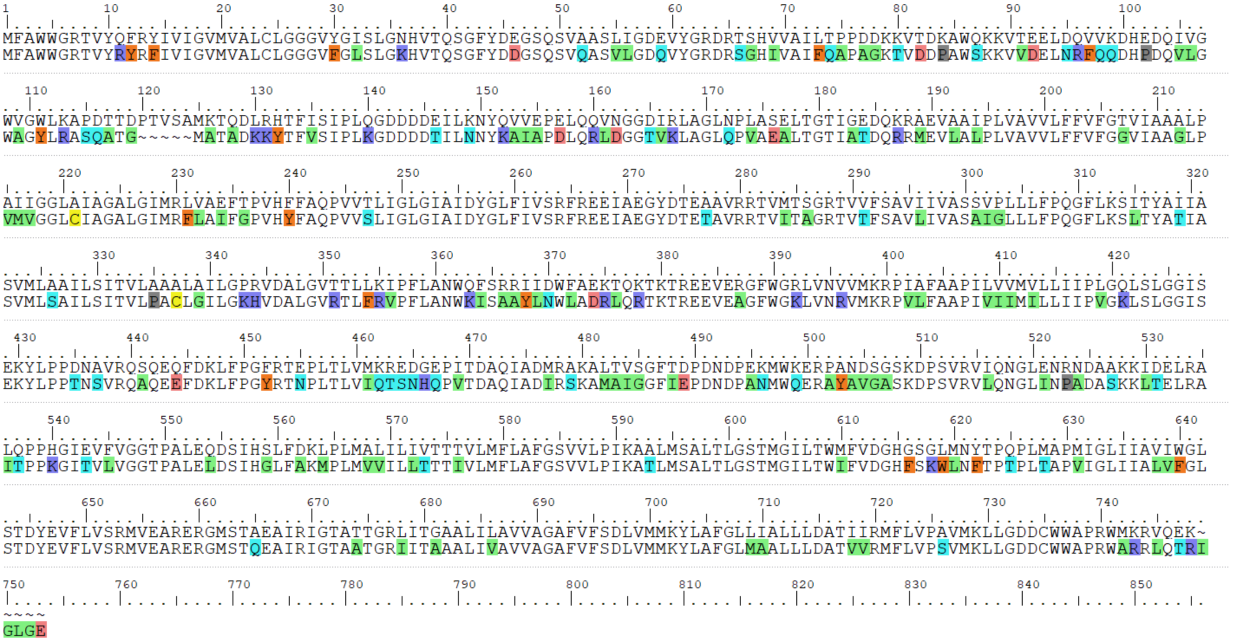

71.9% identity in 748 residues

Figure S4. MmpL3 protein sequence comparison between *M. smegmatis* (top) and *M. tuberculosis* (bottom).

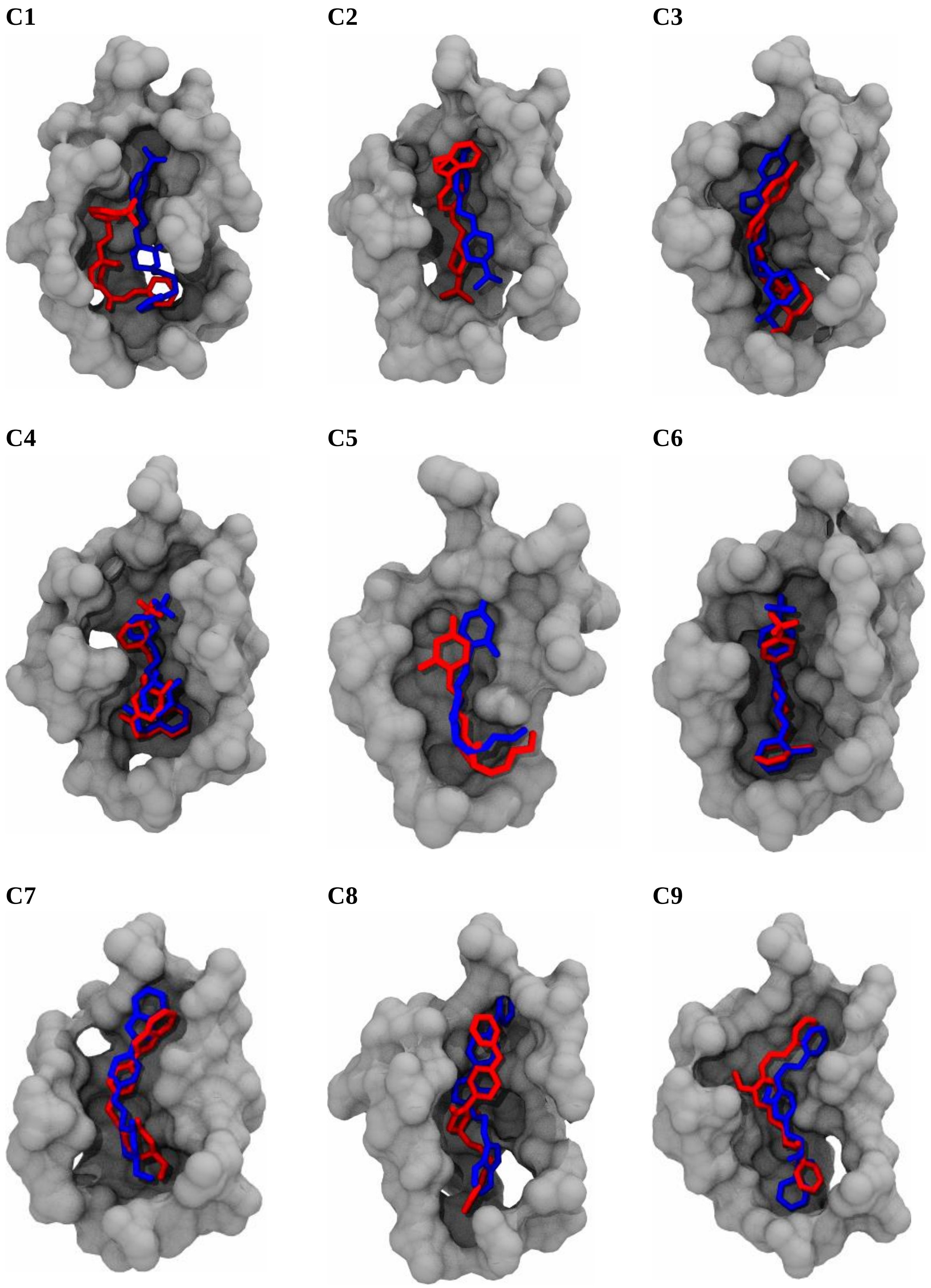
C1
C2
C3
C4
C5
C6
C7
C8
C9

Figure S5. Comparison of ligand XP docking binding pose before (blue) and after (red) MD simulations of the top nine ligands.

Compound	2D Structure/ SMILES	Docking score (kcal/mol)	# Cluster ID
Ref. Comp<br>SQ109	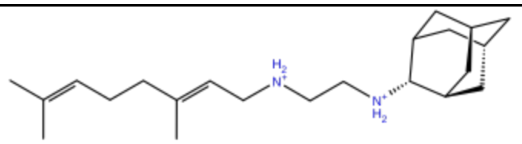<br>CC(=CCC/C(=C/CNCCNC1C2CC3CC(C2)CC1C3)/C)C	-13.757	8
C1<br>ZINC585283799	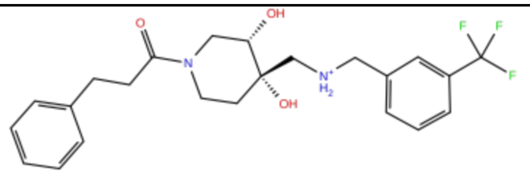<br>c1ccccc1CCC(=O)N(CC2)C[C@H](O)[C@@]2(O)C[NH2+]Cc(cc3C(F)(F)F)ccc3	-14.956	8
C2<br>ZINC12533192	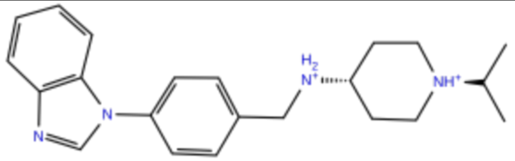<br>CC(C)[N@H+](CC1)CC[C@@H]1[NH2+]Cc2ccc(cc2)-n(cn3)c(c34)cccc4	-14.307	5
C3<br>ZINC248146645	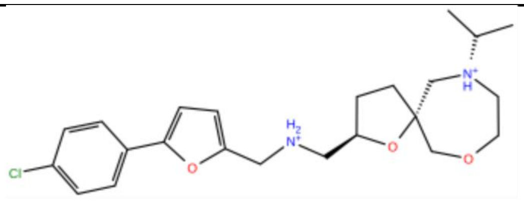<br>C1OCC[N@@H+](C(C)C)C[C@]12CC[C@@H](O2)C[NH2+]Cc3ccc(o3)-c4ccc(Cl)cc4	-14.213	8
C4<br>ZINC585283127	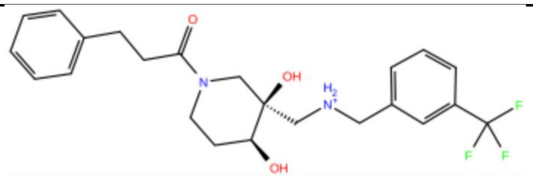<br>c1ccccc1CCC(=O)N(C2)CC[C@H](O)[C@@]2(O)C[NH2+]Cc(cc3C(F)(F)F)ccc3	-14.209	8

C5<br>ZINC14741919	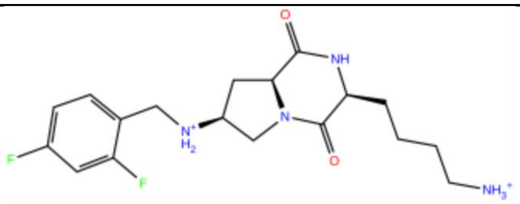<br>[NH3+]CCCC[C@H](NC1=O)C(=O)N([C@H]12)C[C@H](C2)[NH2+]Cc3c(F)cc(F)cc3	-14.201	8
C6<br>ZINC19832139	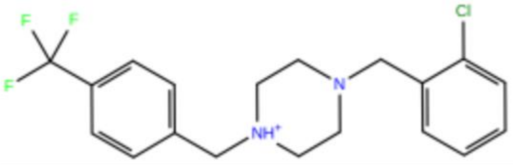<br>FC(F)(F)c1ccc(cc1)C[NH+](CC2)CCN2Cc3c(Cl)cccc3	-14.166	3
C7<br>ZINC221897042	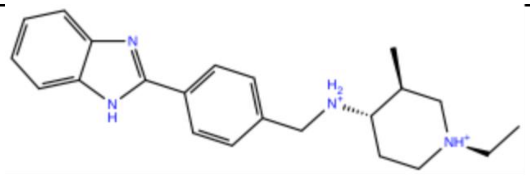<br>CC[N@H+](C1)CC[C@@H]([C@H]1C)[NH2+]Cc2ccc(cc2)-c(n3)[nH]c(c34)cccc4	-13.999	5
C8<br>ZINC18223081	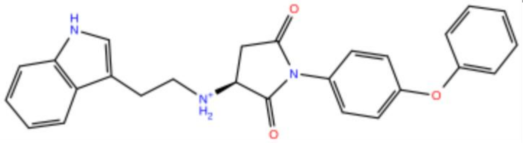<br>c1cccc(c12)[nH]cc2CC[NH2+][C@H](C3=O)CC(=O)N3c4ccc(cc4)Oc5ccccc5	-13.989	8
C9<br>ZINC22107671	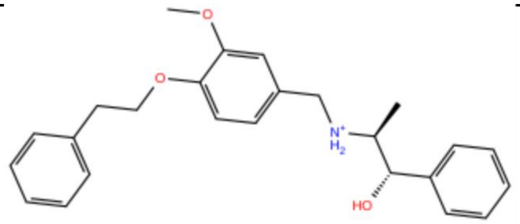<br>c1ccccc1[C@H](O)[C@H](C)[NH2+]Cc2cc(OC)c(cc2)OCCc3ccccc3	-13.896	8

Figure S6. The table represents Zinc ID, structure, SMILE code, docking score, and cluster IDs (ligand similarity clustering is based on Canvas) of the top nine zinc compounds and the reference compound.

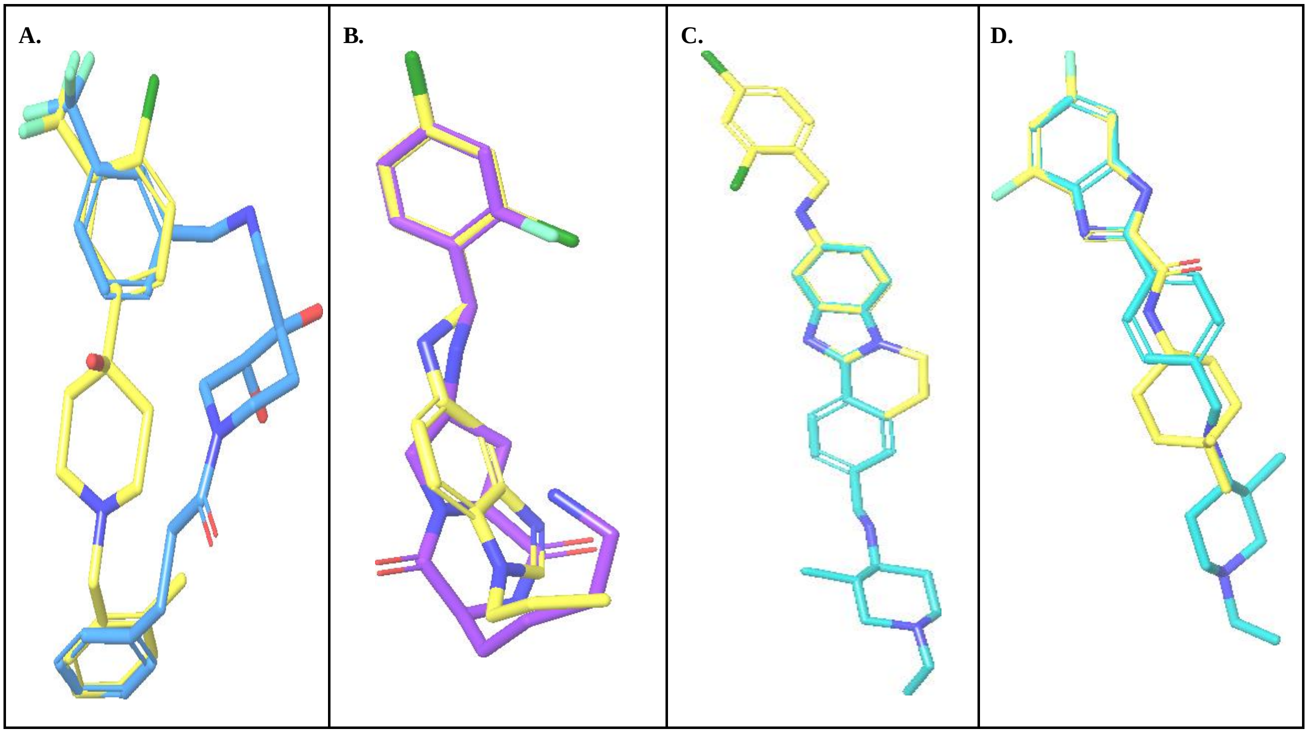

Figure S7. Ligand conformation **c**omparison between **(A)** C1 (blue) and PIPD1 (yellow), **(B)** C5 (purple) and C215 (yellow), **(C)** C7 (cyan) and C215, and **(D)** C7 and NITD-349 (yellow).

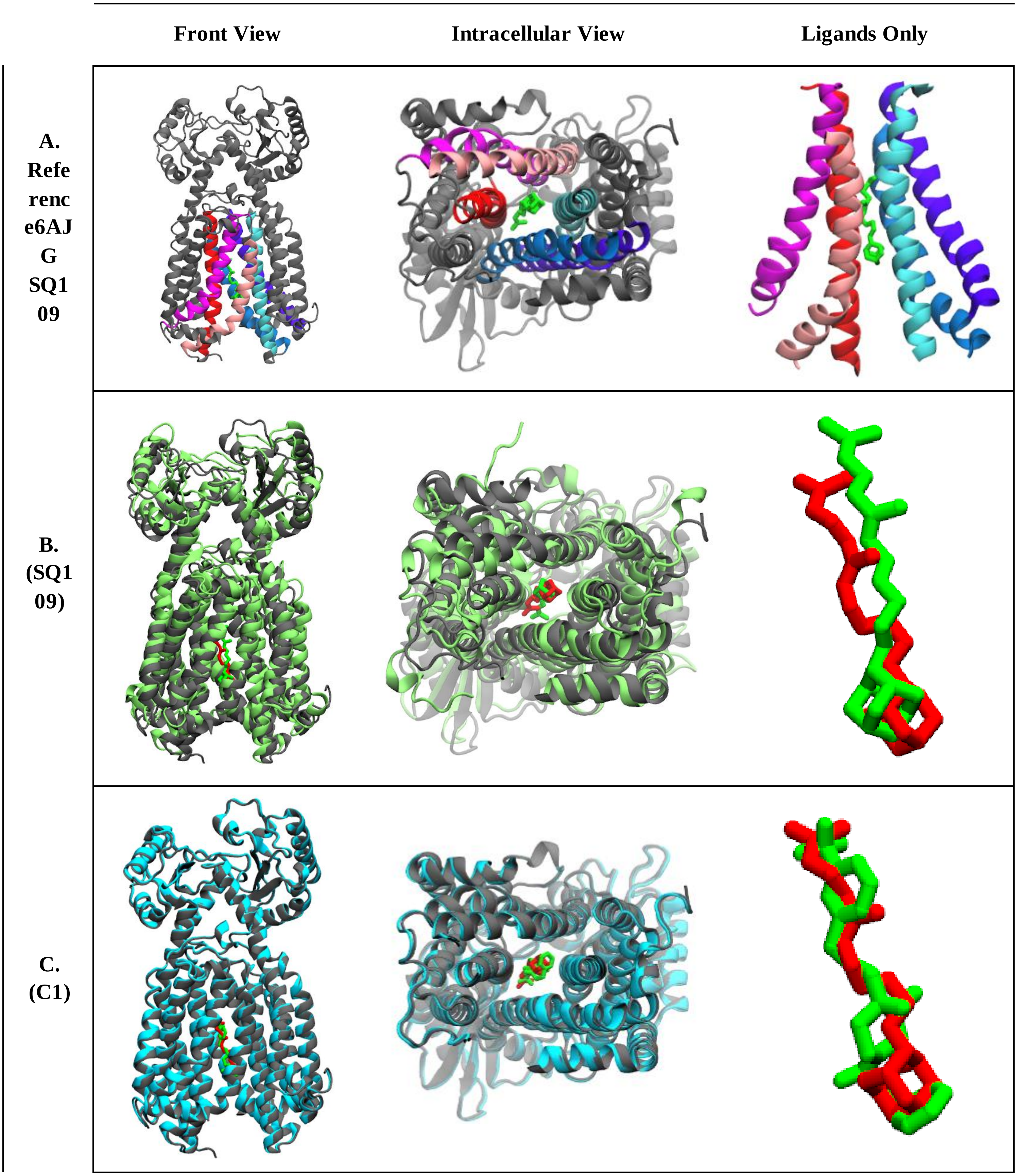
Front View
Intracellular View
Ligands Only
A.
Refe
renc
e6AJ
G
SQ1
09
B.
(SQ1
09)
C.
(C1)

D. (C3)

E. (C7)

G. (C9)

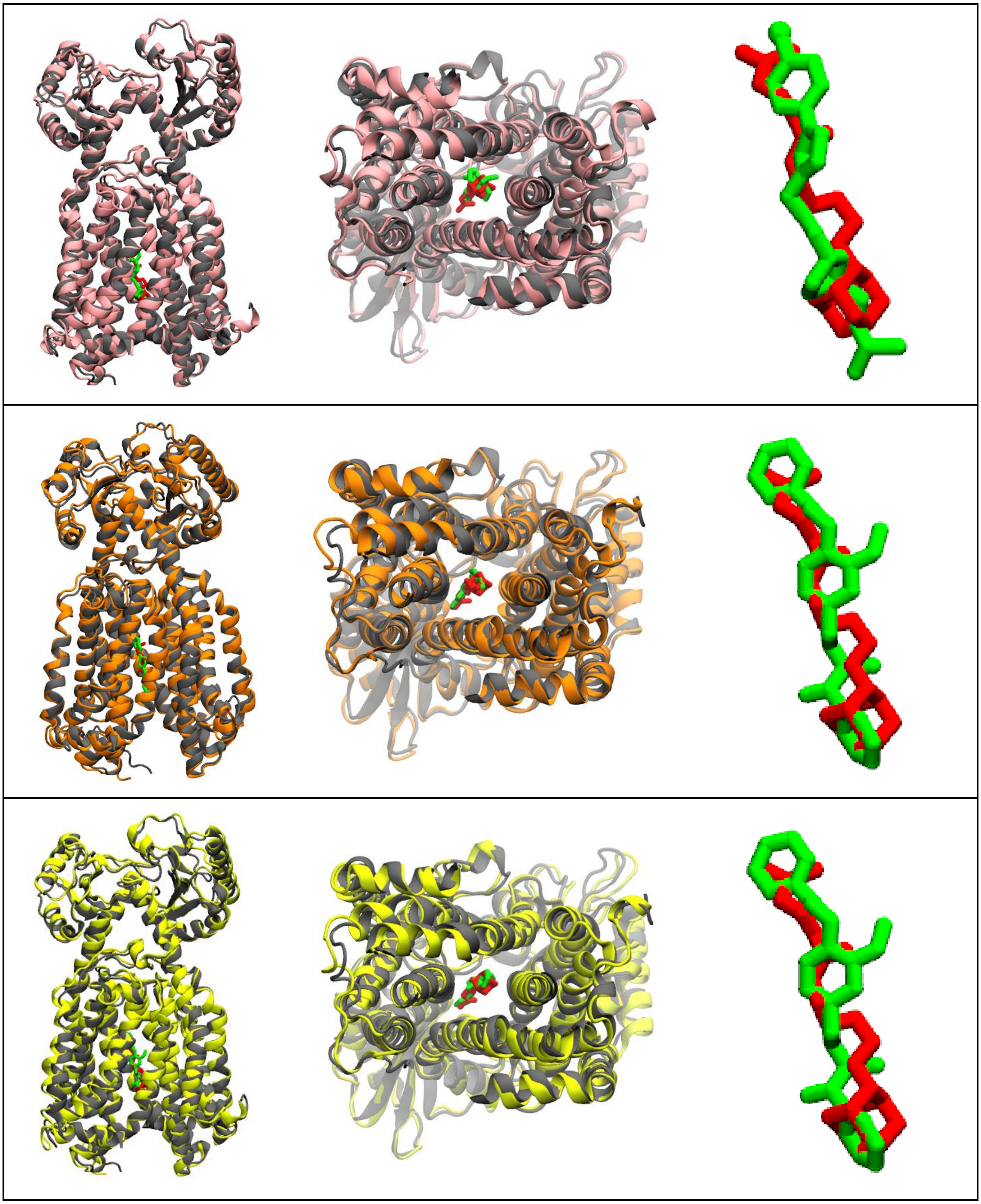

Figure S9. Structure alignment of Crystal Ligand and most abundant structures of SQ109 and top 4 ligands. **A**. 6AJG Crystal structure with channel lining transmembrane helices colored as protein (Grey) TM4 (Cyan) TM5 (Blue) TM6 (Violet) TM10 (Red) TM11 (Pink) TM12 (Magenta) and SQ109 (Green) **B-G**. Reference Crystal Structure (Grey) and Ligand SQ109 (Red) Shown against post MD structures for C1, C2, C3, and C9 as (Green). Most abundant Cluster of structure 6AJG (Lime) and SQ109 Ligand (Green). **C**. Most abundant Cluster structure of Ligand C1 (Cyan) and C1 Ligand (Green). **D**. Most abundant cluster structure and C3 Ligand (Pink) and C3 Ligand (Green). **E**. Most abundant cluster structure (Orange) and Ligand C7 (Green). **G**. Most abundant Cluster structure (Yellow) and Ligand C9 (Green).

C1

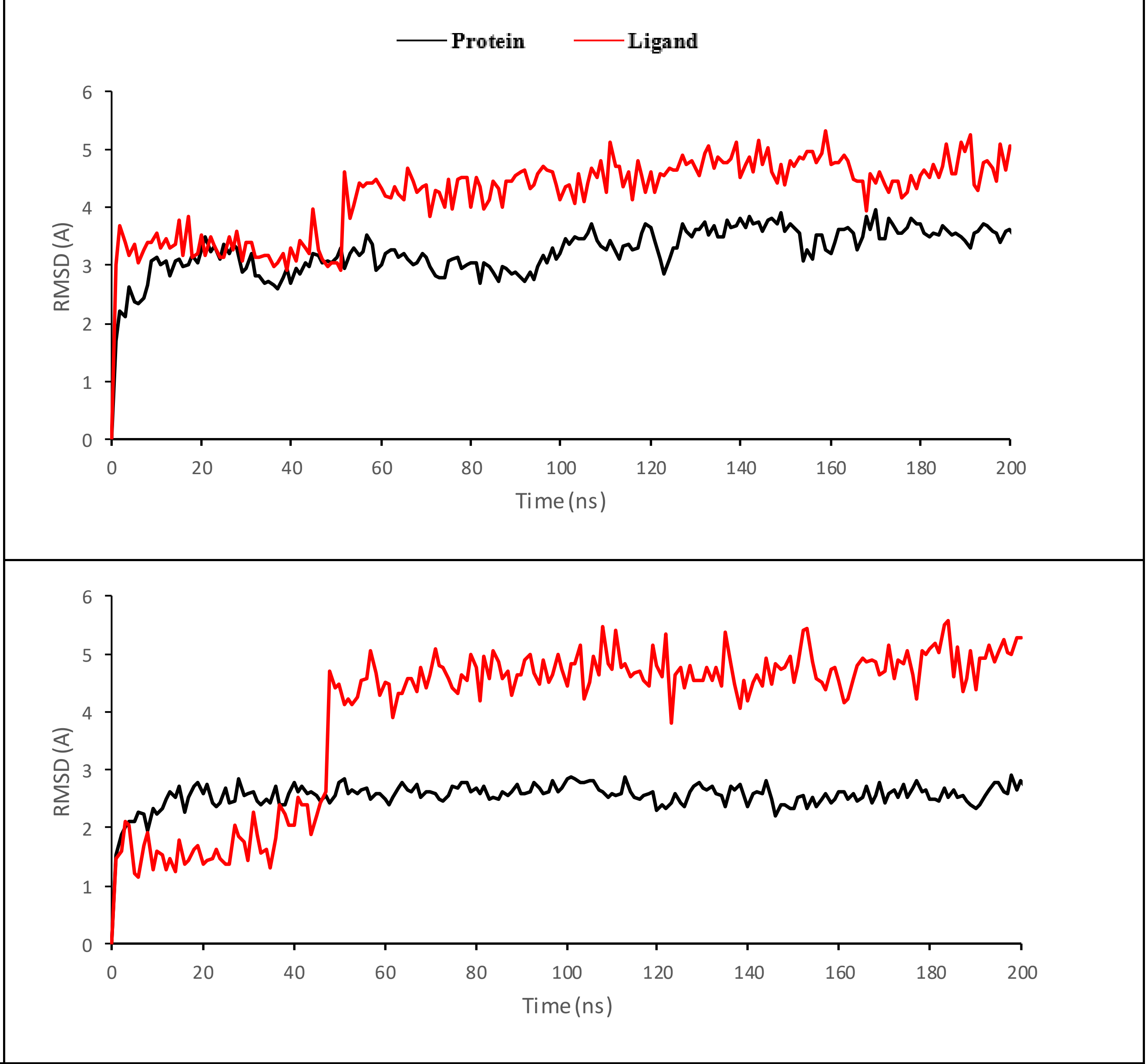

C
2

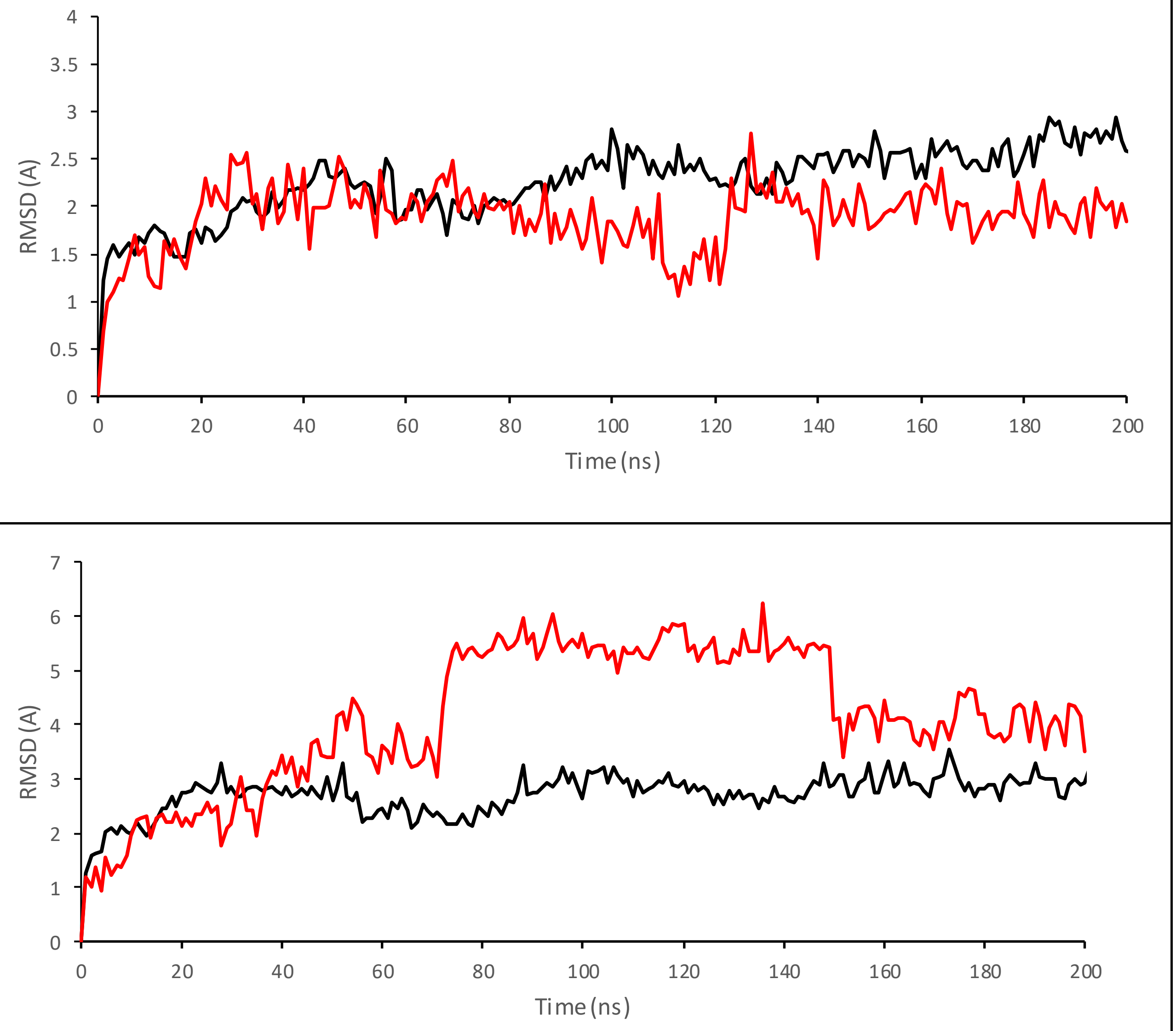

4
3.5
3
2.5
2
1.5
1
0.5
0
RMSD (A)
0
20
40
60
80
100
120
140
160
180
200
Time (ns)
7
6
5
4
3
2
1
0
RMSD (A)
Time (ns)

C
3

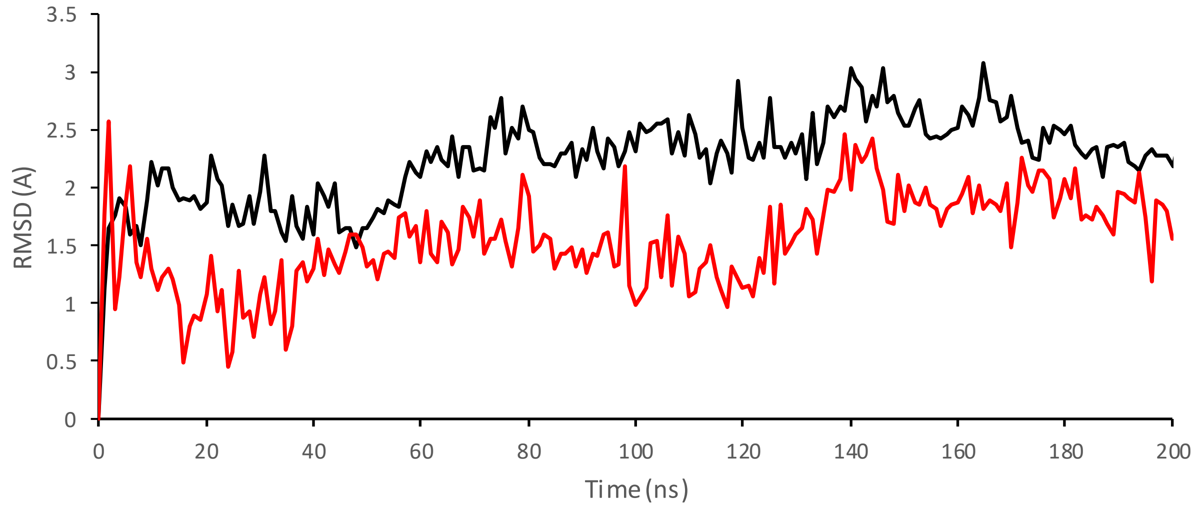

RMSD (A)
0
0.5
1
1.5
2
2.5
3
3.5
0
20
40
60
80
100
120
140
160
180
200
Time (ns)

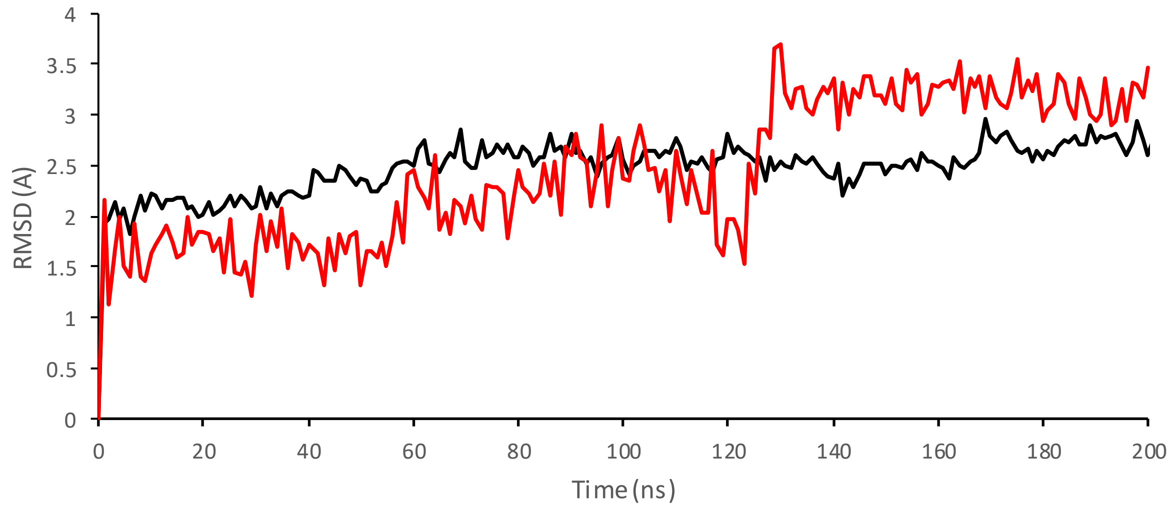

RMSD (A)
0
0.5
1
1.5
2
2.5
3
3.5
4
0
20
40
60
80
100
120
140
160
180
200
Time (ns)

C
4

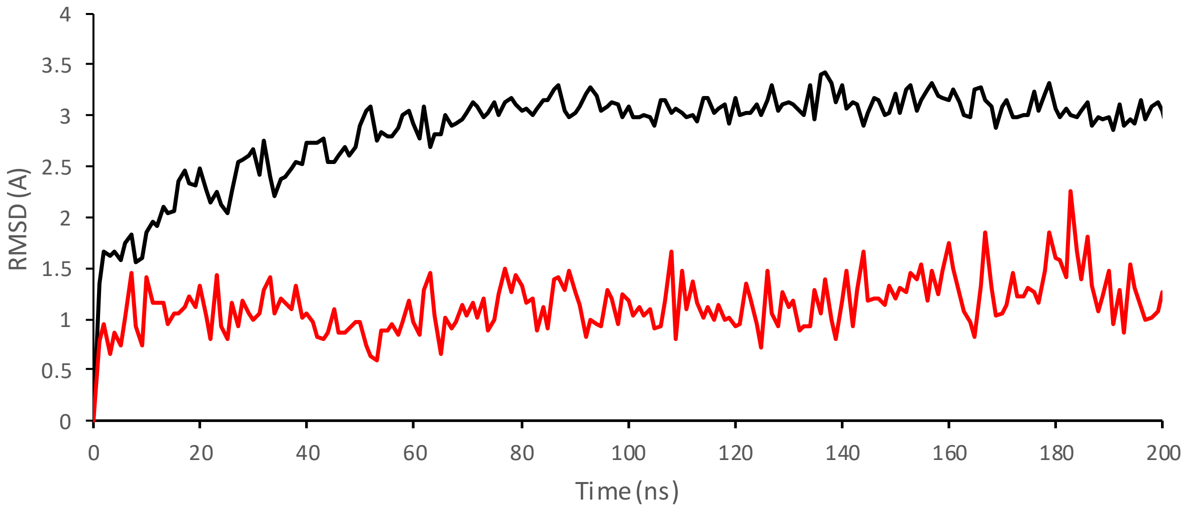
4
3.5
3
2.5
2
1.5
1
0.5
0
RMSD (A)
0 20 40 60 80 100 120 140 160 180 200
Time (ns)

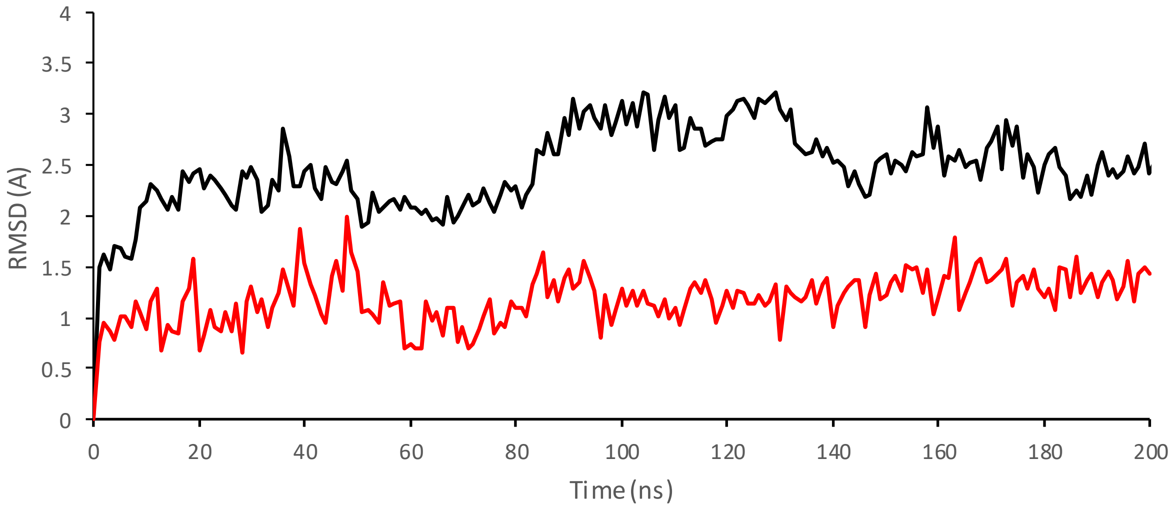
4
3.5
3
2.5
2
1.5
1
0.5
0
RMSD (A)
0 20 40 60 80 100 120 140 160 180 200
Time (ns)

C 5

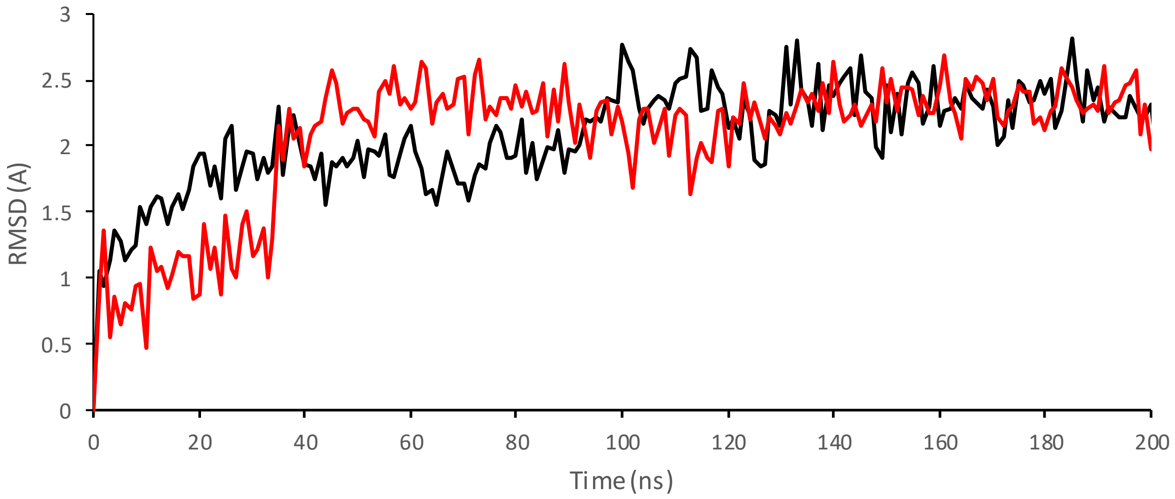
RMSD (A)
Time (ns)
0
0.5
1
1.5
2
2.5
3
0
20
40
60
80
100
120
140
160
180
200

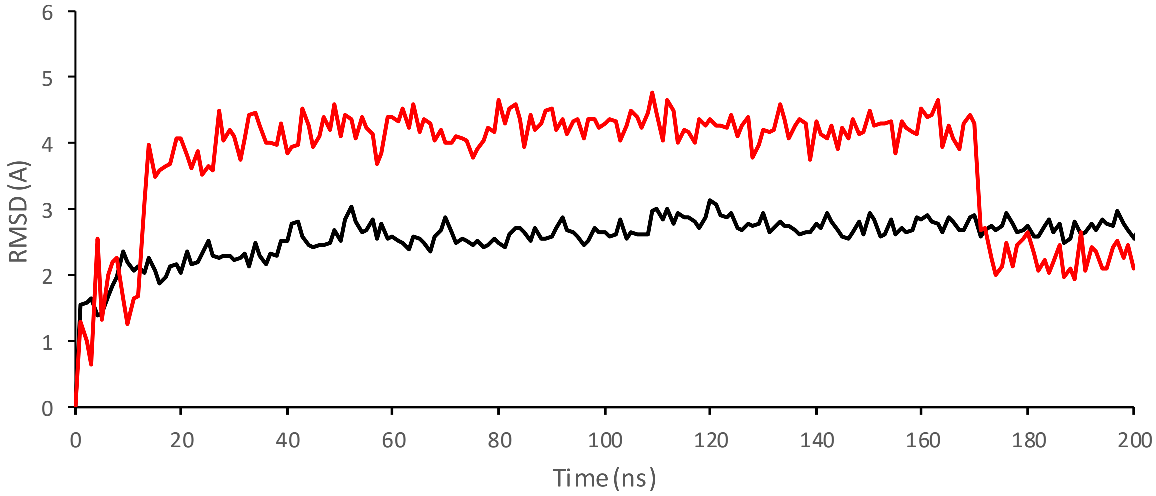
RMSD (A)
Time (ns)
0
1
2
3
4
5
6
0
20
40
60
80
100
120
140
160
180
200

C 6

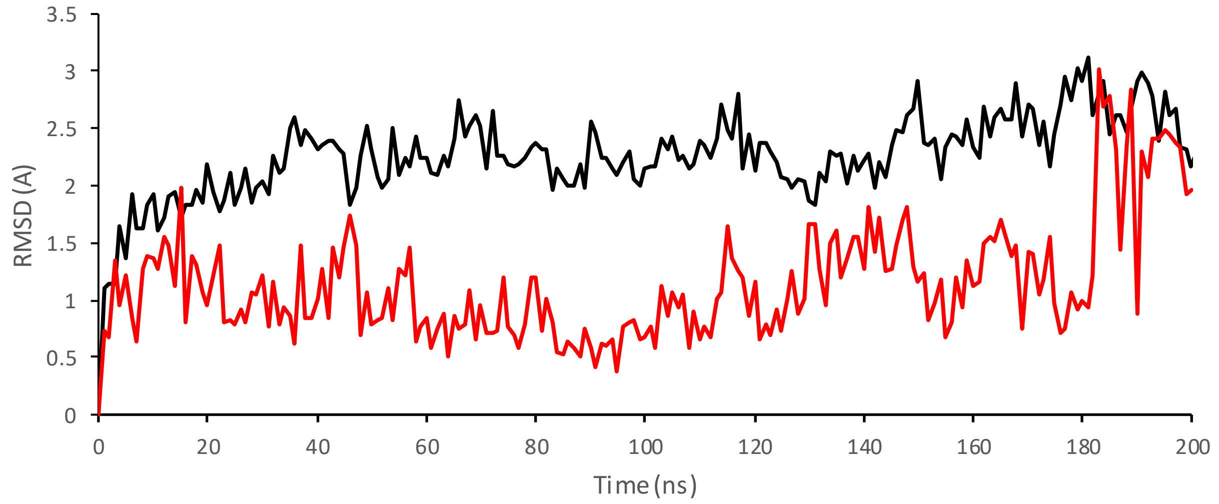
RMSD (A)
0
0.5
1
1.5
2
2.5
3
3.5
0
20
40
60
80
100
120
140
160
180
200
Time (ns)

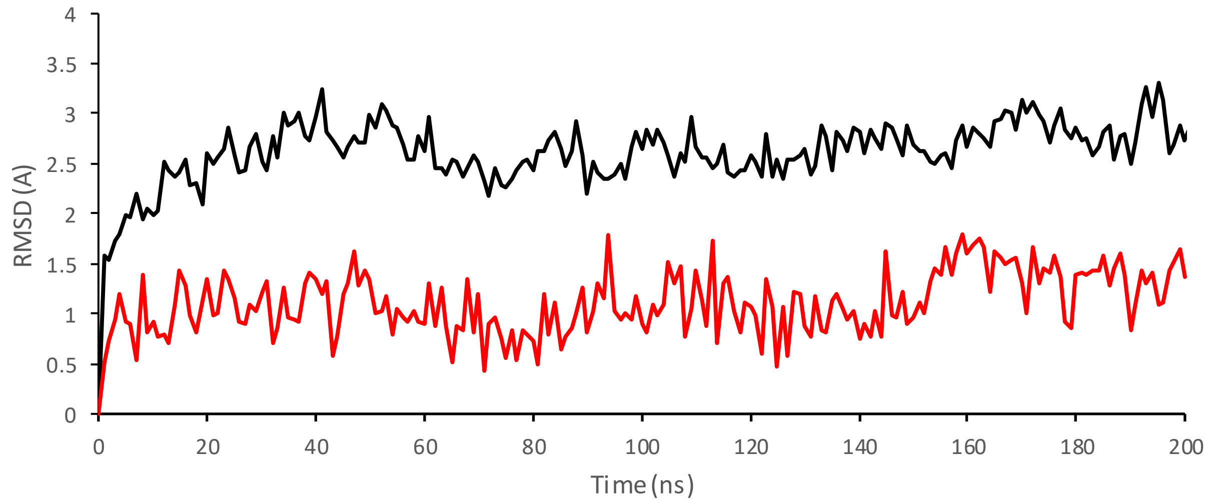
RMSD (A)
0
0.5
1
1.5
2
2.5
3
3.5
4
0
20
40
60
80
100
120
140
160
180
200
Time (ns)

C7

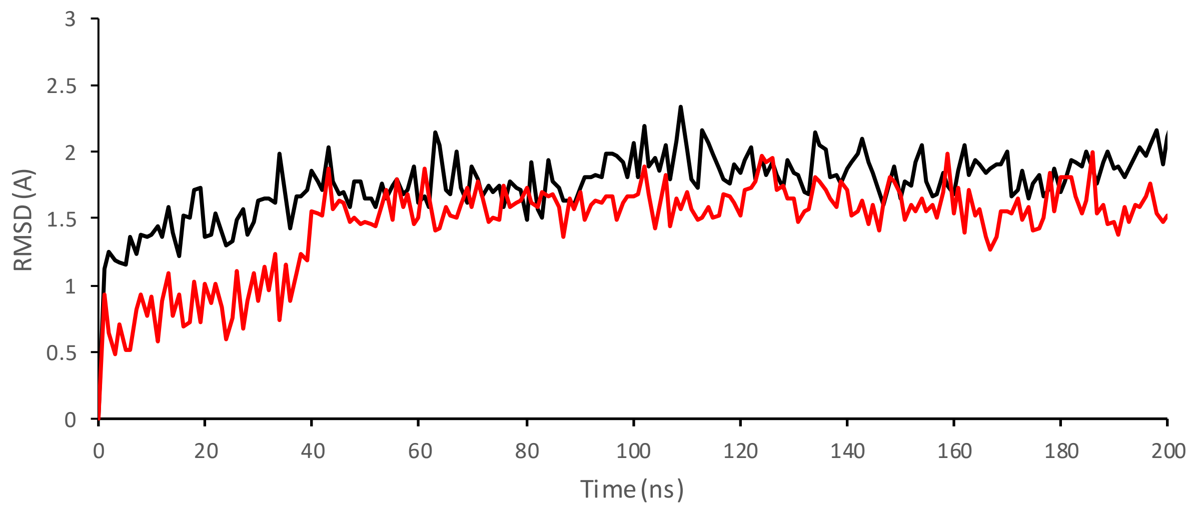

RMSD (A)
Time (ns)
3
2.5
2
1.5
1
0.5
0
0
20
40
60
80
100
120
140
160
180
200

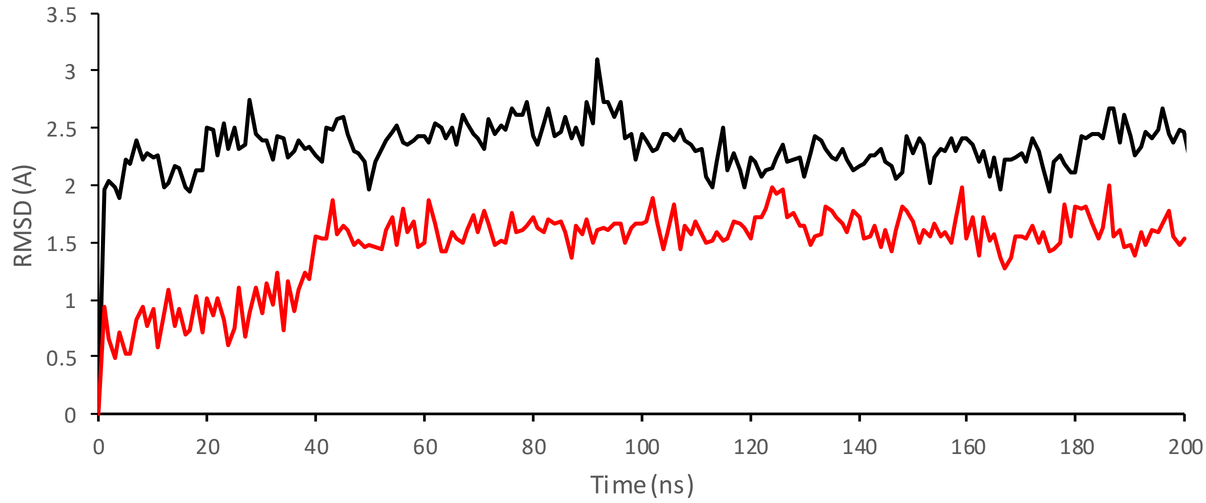

RMSD (A)
Time (ns)
3.5
3
2.5
2
1.5
1
0.5
0
0
20
40
60
80
100
120
140
160
180
200

C
8

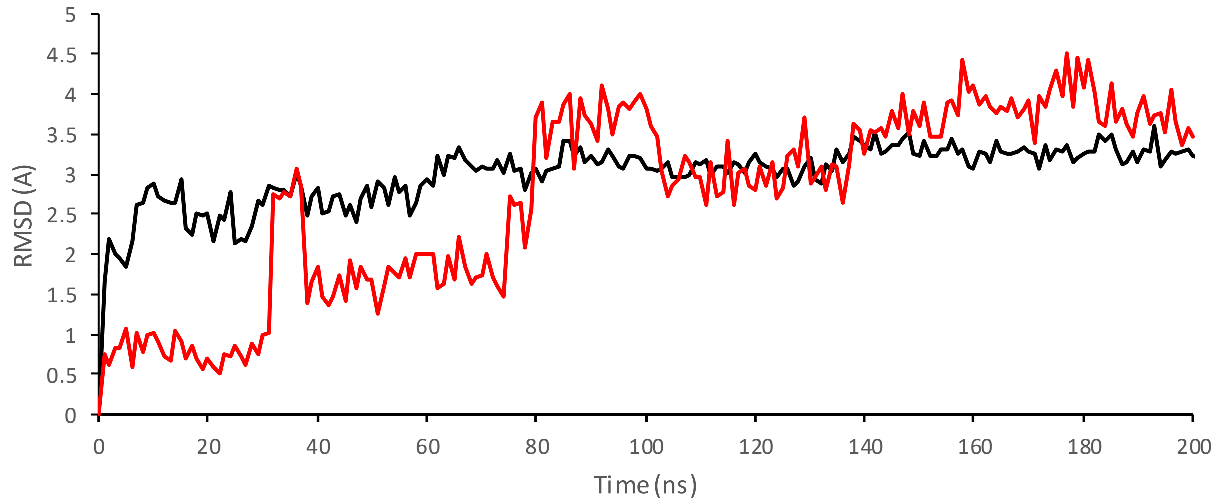

RMSD (A)
0
0.5
1
1.5
2
2.5
3
3.5
4
4.5
5
0
20
40
60
80
100
120
140
160
180
200
Time (ns)

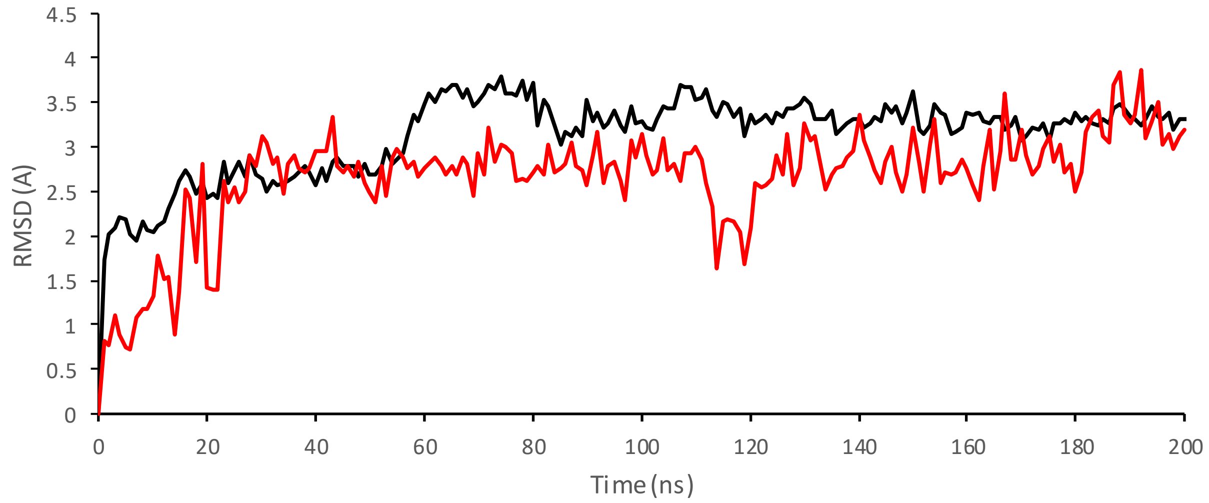

RMSD (A)
0
0.5
1
1.5
2
2.5
3
3.5
4
4.5
0
20
40
60
80
100
120
140
160
180
200
Time (ns)

C 9

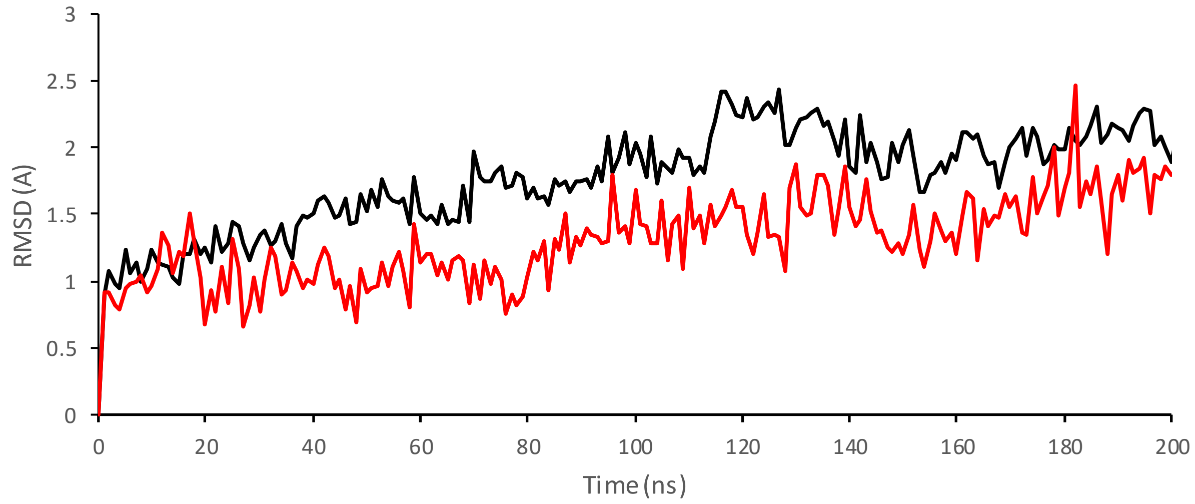

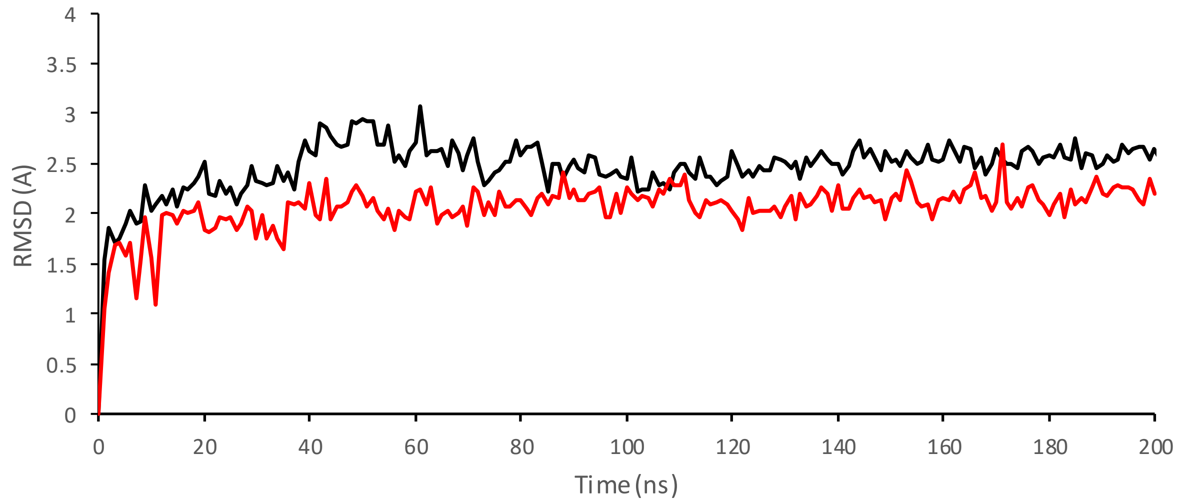

Figure S10. Protein-ligand RMSD of the top nine compounds of two ~200 ns MD simulations.

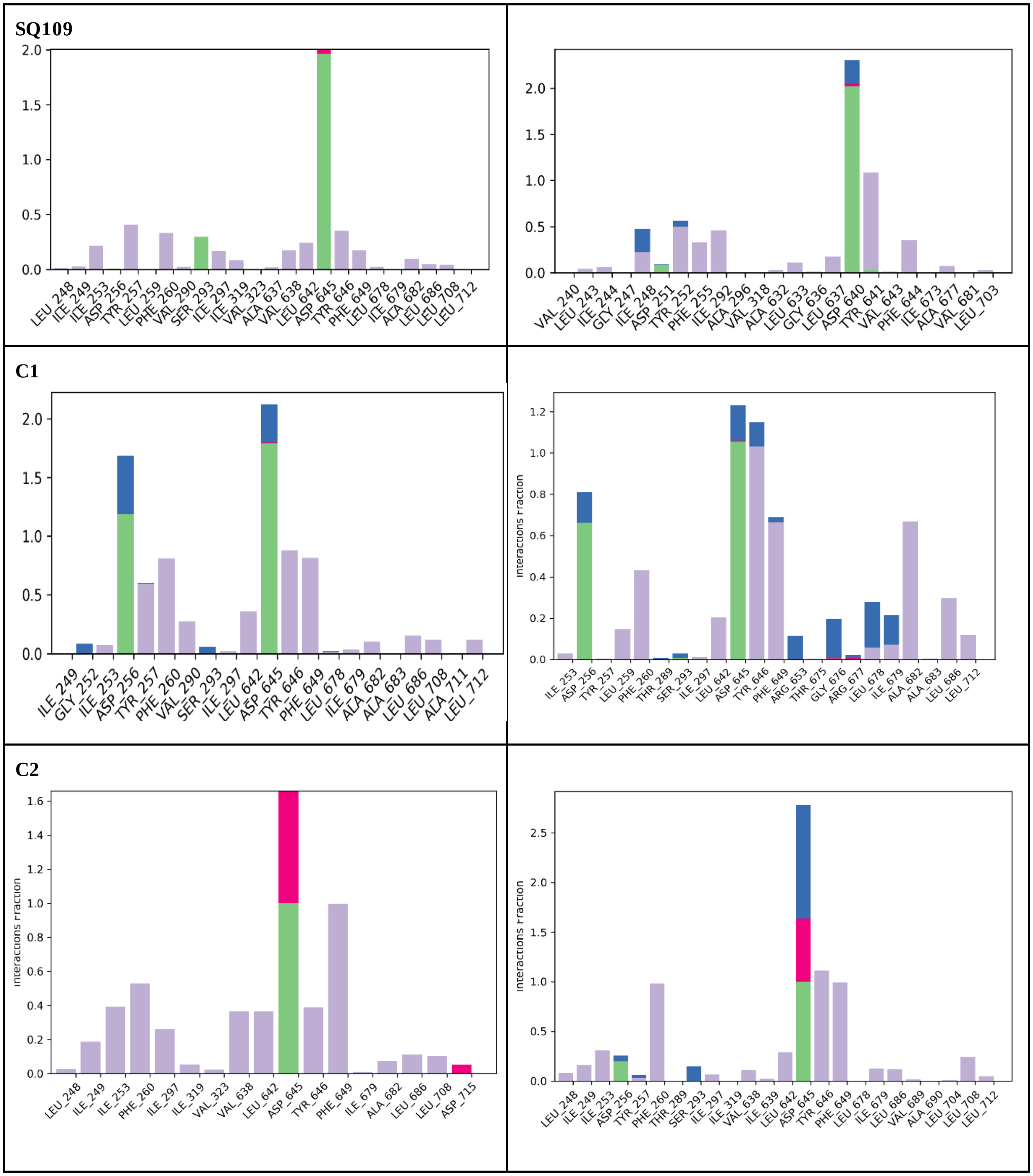
SQ109
C1
C2
Interactions Fraction

C3

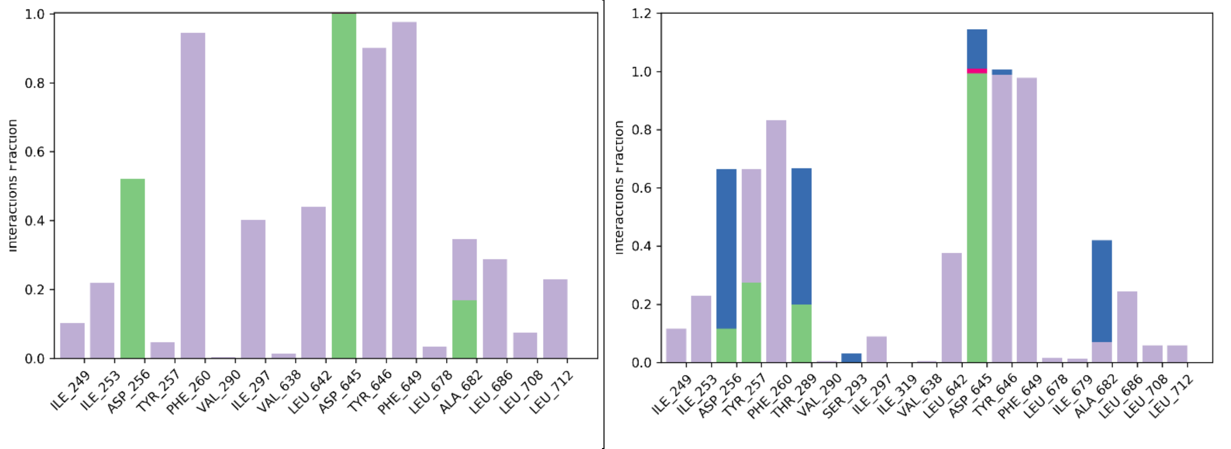
Interactions Fraction
ILE_249
ILE_253
ASP_256
TYR_257
PHE_260
VAL_290
ILE_297
VAL_638
LEU_642
ASP_645
TYR_646
PHE_649
LEU_678
ALA_682
LEU_686
LEU_708
LEU_712
Interactions Fraction
ILE_249
ILE_253
ASP_256
TYR_257
PHE_260
THR_289
VAL_290
SER_293
ILE_297
ILE_319
VAL_638
LEU_642
ASP_645
TYR_646
PHE_649
LEU_678
ILE_679
ALA_682
LEU_686
LEU_708
LEU_712

C4

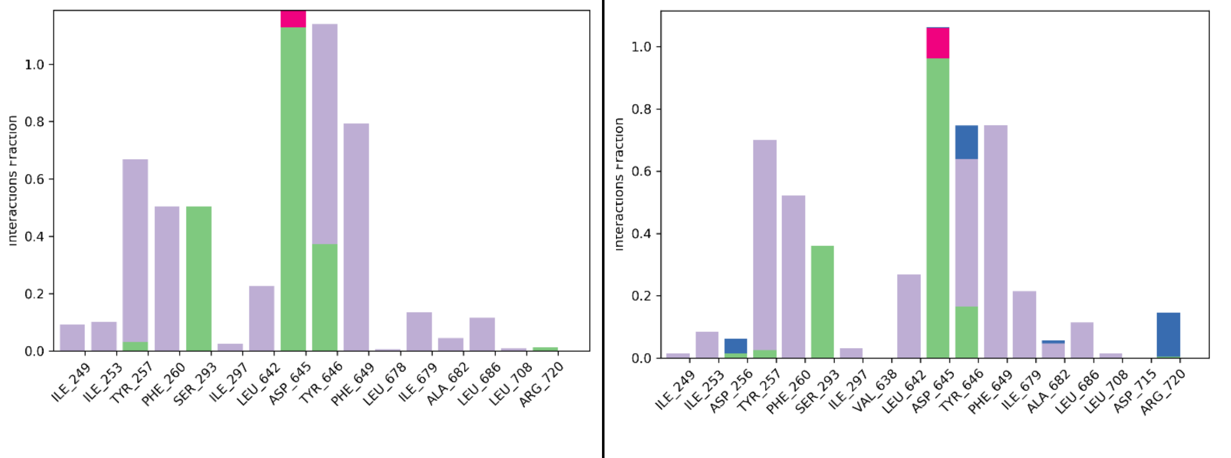
Interactions Fraction
ILE_249
ILE_253
TYR_257
PHE_260
SER_293
ILE_297
LEU_642
ASP_645
TYR_646
PHE_649
LEU_678
ILE_679
ALA_682
LEU_686
LEU_708
ARG_720
Interactions Fraction
ILE_249
ILE_253
ASP_256
TYR_257
PHE_260
SER_293
ILE_297
VAL_638
LEU_642
ASP_645
TYR_646
PHE_649
ILE_679
ALA_682
LEU_686
LEU_708
ASP_715
ARG_720

C5

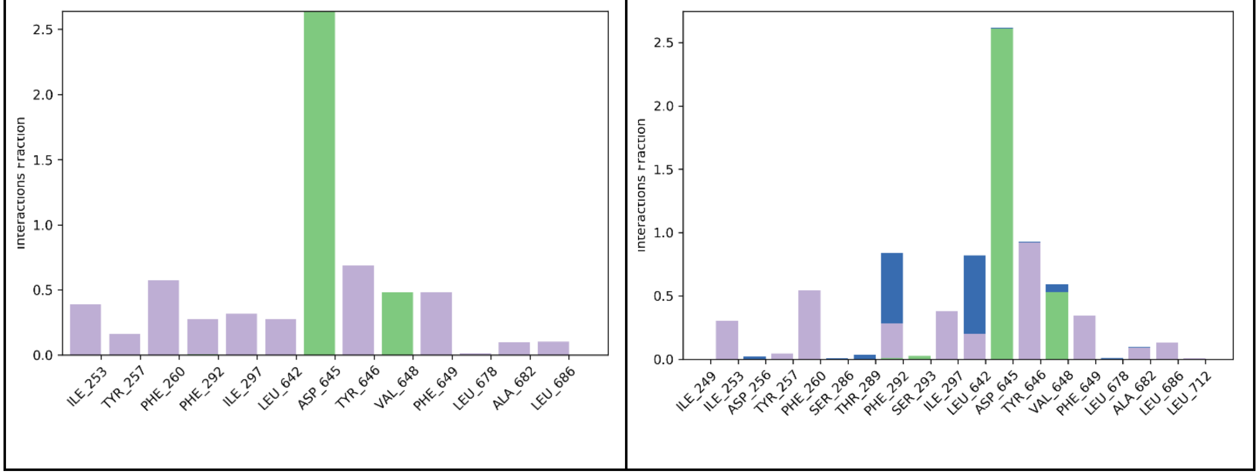
Interactions Fraction
ILE_253
TYR_257
PHE_260
PHE_292
ILE_297
LEU_642
ASP_645
TYR_646
VAL_648
PHE_649
LEU_678
ALA_682
LEU_686
Interactions Fraction
ILE_249
ILE_253
ASP_256
TYR_257
PHE_260
SER_286
THR_289
PHE_292
SER_293
ILE_297
LEU_642
ASP_645
TYR_646
VAL_648
PHE_649
LEU_678
ALA_682
LEU_686
LEU_712

C6

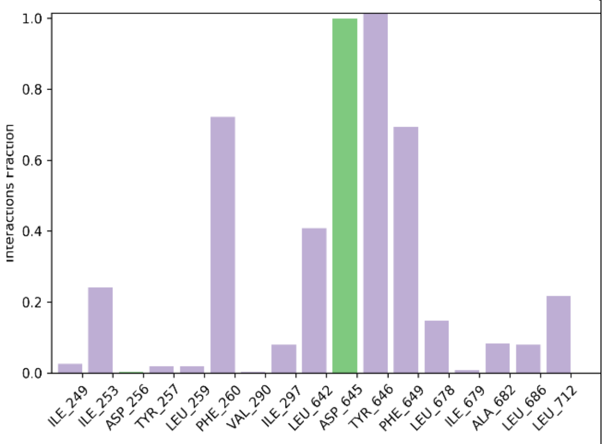
Interactions Fraction
ILE_249 ILE_253 ASP_256 TYR_257 LEU_259 PHE_260 VAL_290 ILE_297 LEU_642 ASP_645 TYR_646 PHE_649 LEU_678 ILE_679 ALA_682 LEU_686 LEU_712

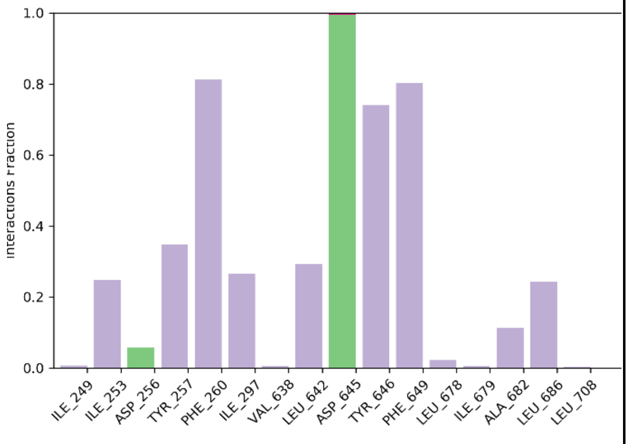
Interactions Fraction
ILE_249 ILE_253 ASP_256 TYR_257 PHE_260 ILE_297 VAL_638 LEU_642 ASP_645 TYR_646 PHE_649 LEU_678 ILE_679 ALA_682 LEU_686 LEU_708

C7

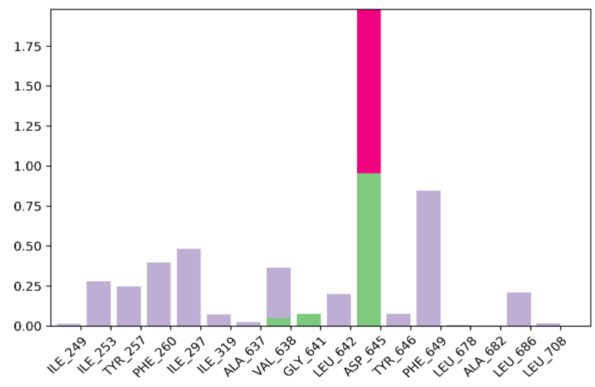
ILE_249 ILE_253 TYR_257 PHE_260 ILE_297 ILE_319 ALA_637 VAL_638 GLY_641 LEU_642 ASP_645 TYR_646 PHE_649 LEU_678 ALA_682 LEU_686 LEU_708

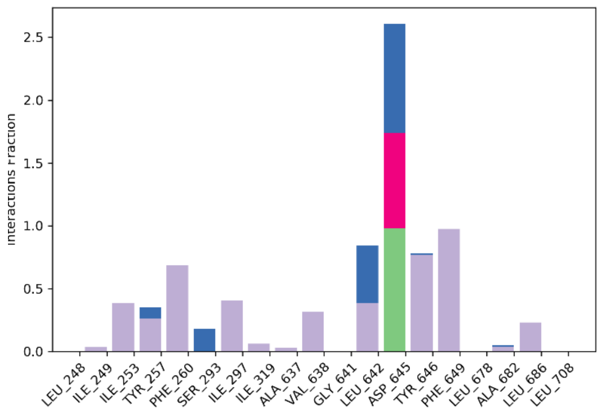
Interactions Fraction
LEU_248 ILE_249 ILE_253 TYR_257 PHE_260 SER_293 ILE_297 ILE_319 ALA_637 VAL_638 GLY_641 LEU_642 ASP_645 TYR_646 PHE_649 LEU_678 ALA_682 LEU_686 LEU_708

C8

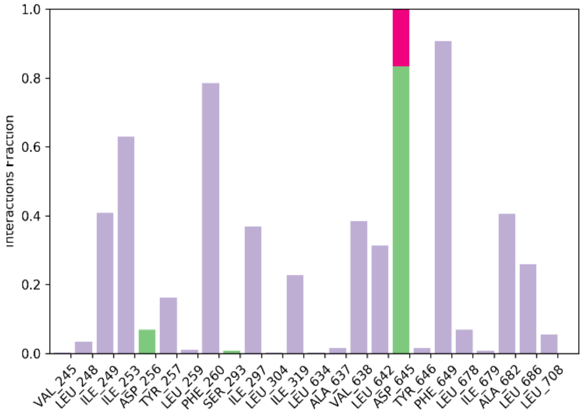
Interactions Fraction
VAL_245 LEU_248 ILE_249 ILE_253 ASP_256 TYR_257 LEU_259 PHE_260 SER_293 ILE_297 LEU_304 ILE_319 LEU_634 ALA_637 VAL_638 LEU_642 ASP_645 TYR_646 PHE_649 LEU_678 ILE_679 ALA_682 LEU_686 LEU_708

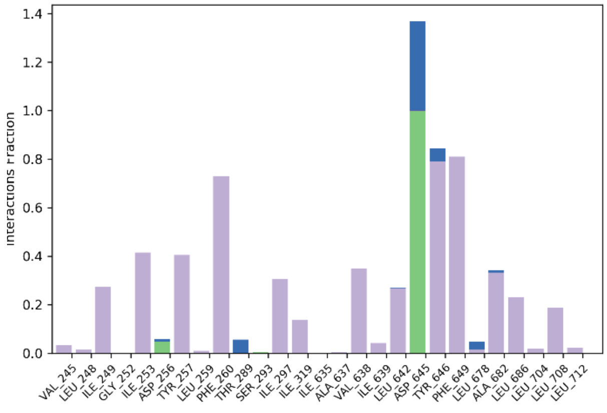
Interactions Fraction
VAL_245 LEU_248 ILE_249 GLY_252 ILE_253 ASP_256 TYR_257 LEU_259 PHE_260 THR_289 SER_293 ILE_297 ILE_319 ILE_635 ALA_637 VAL_638 ILE_639 LEU_642 ASP_645 TYR_646 PHE_649 LEU_678 ALA_682 LEU_686 LEU_704 LEU_708 LEU_712

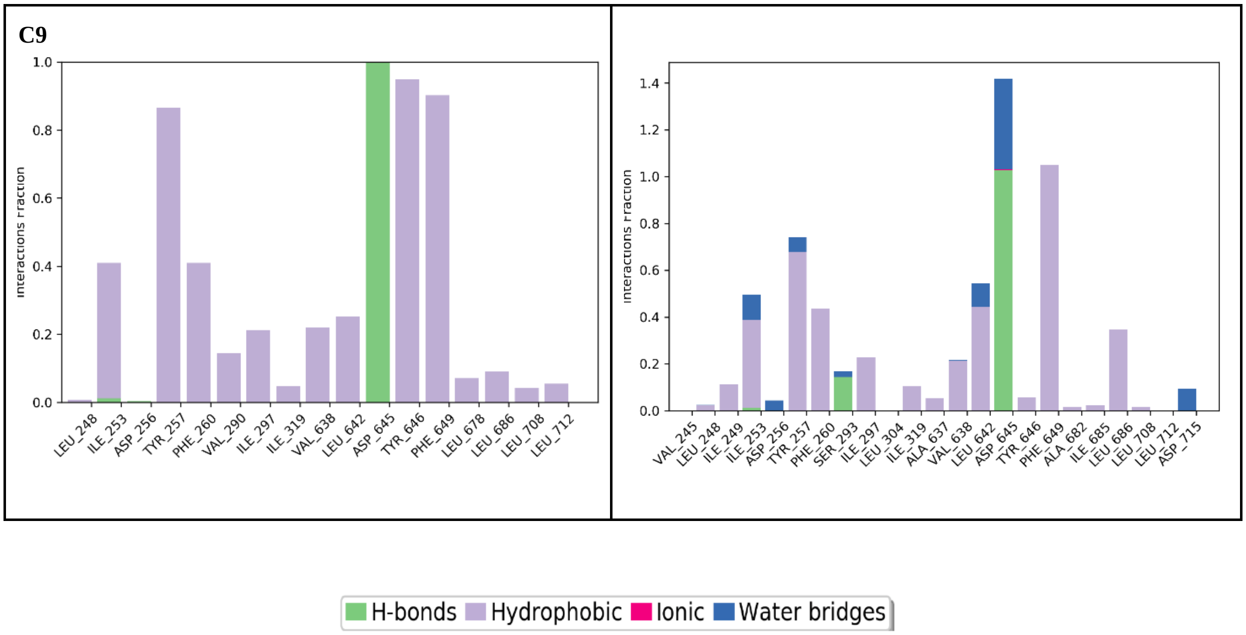

Figure S11. Protein-ligand contacts during MD simulations for the crystal ligand SQ109 and the nine leading compounds. Interaction fraction greater than 1 is possible because of multiple contacts on one residue.

SQ109

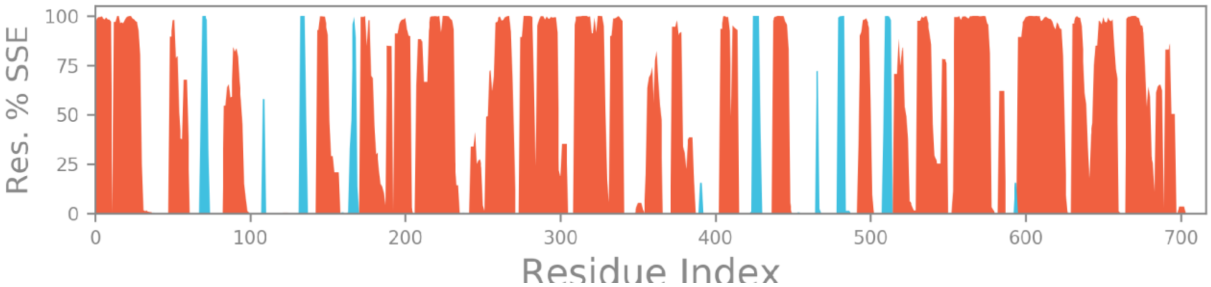
Res. % SSE
0
25
50
75
100
0
100
200
300
400
500
600
700
Residue Index

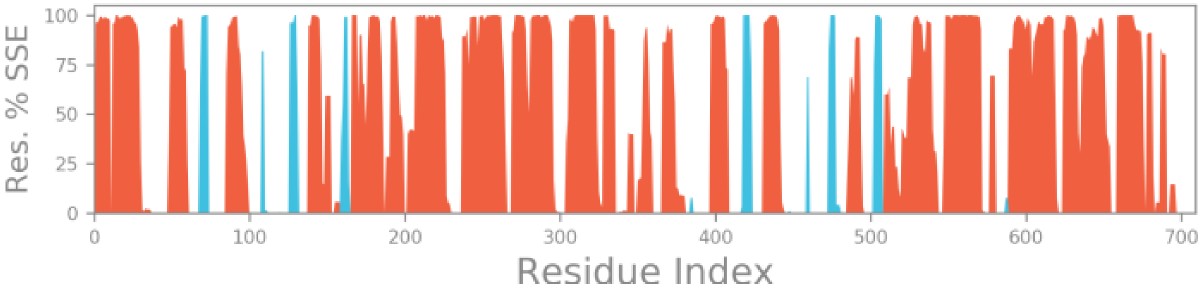
Res. % SSE
0
25
50
75
100
0
100
200
300
400
500
600
700
Residue Index

C1

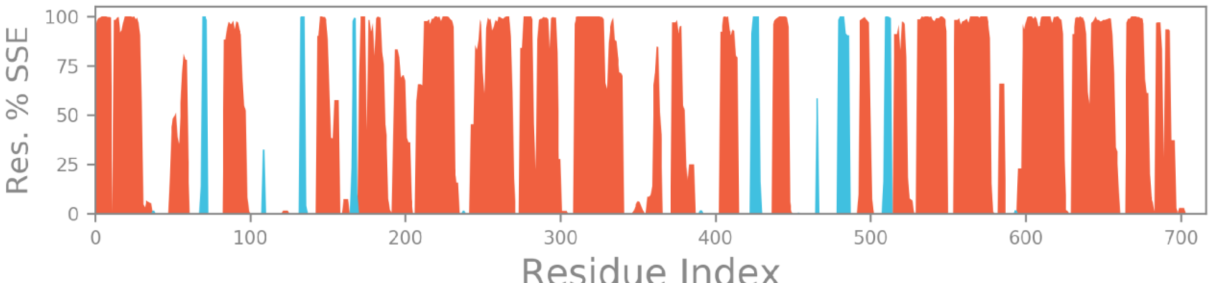
Res. % SSE
0
25
50
75
100
0
100
200
300
400
500
600
700
Residue Index

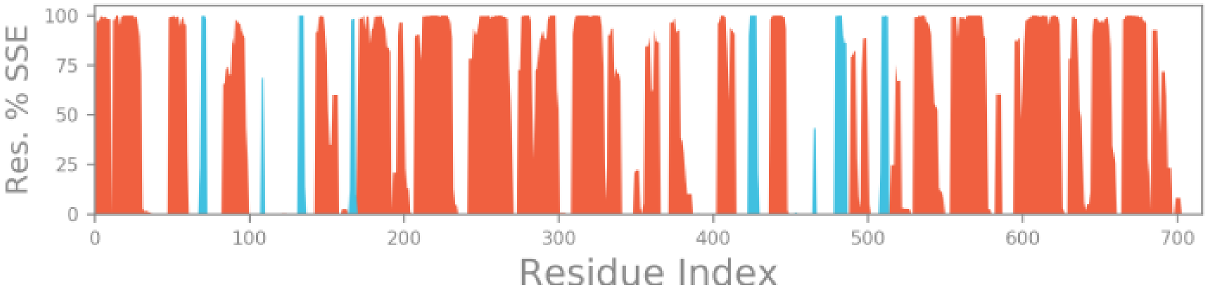
Res. % SSE
0
25
50
75
100
0
100
200
300
400
500
600
700
Residue Index

C2

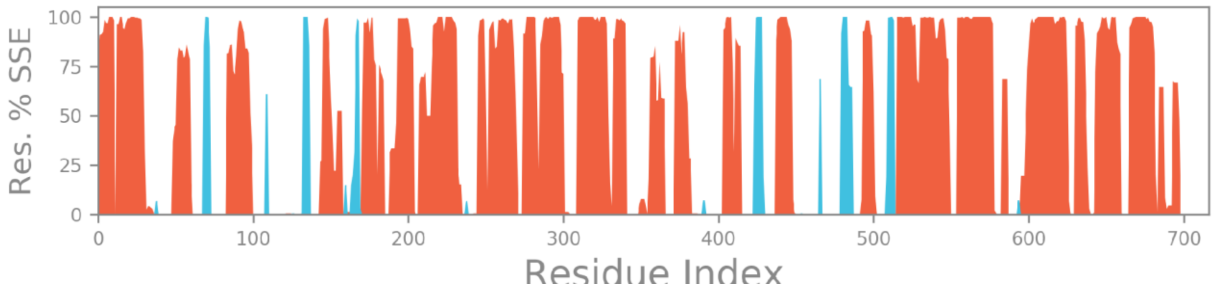
Res. % SSE
100
75
50
25
0
0
100
200
300
400
500
600
700
Residue Index

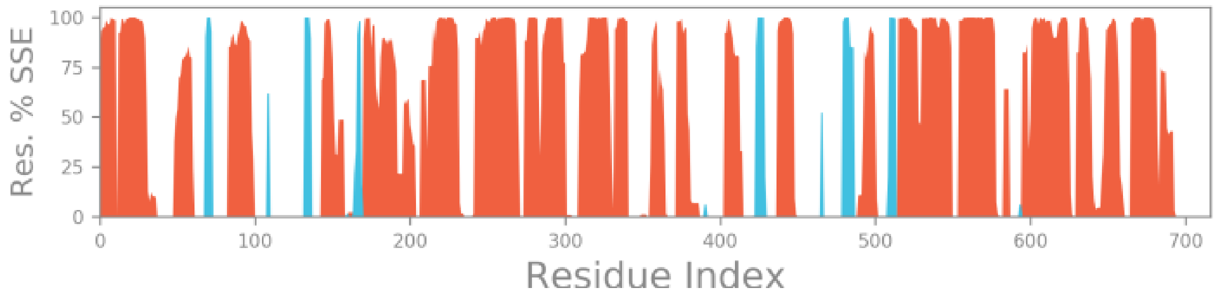
Res. % SSE
100
75
50
25
0
0
100
200
300
400
500
600
700
Residue Index

C3

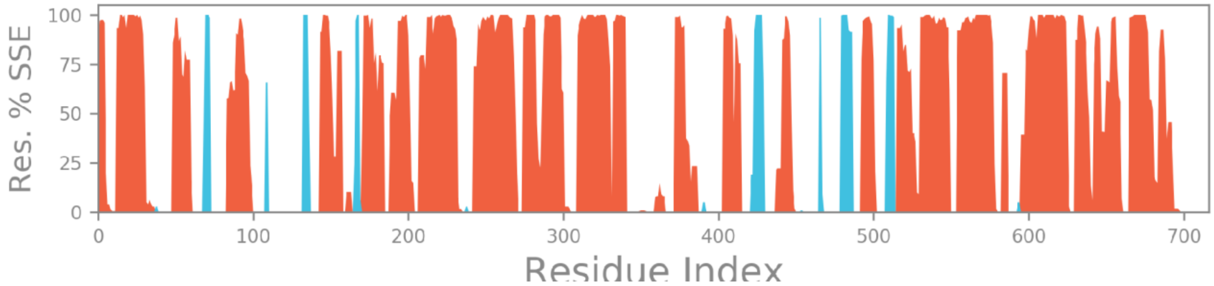
Res. % SSE
100
75
50
25
0
0
100
200
300
400
500
600
700
Residue Index

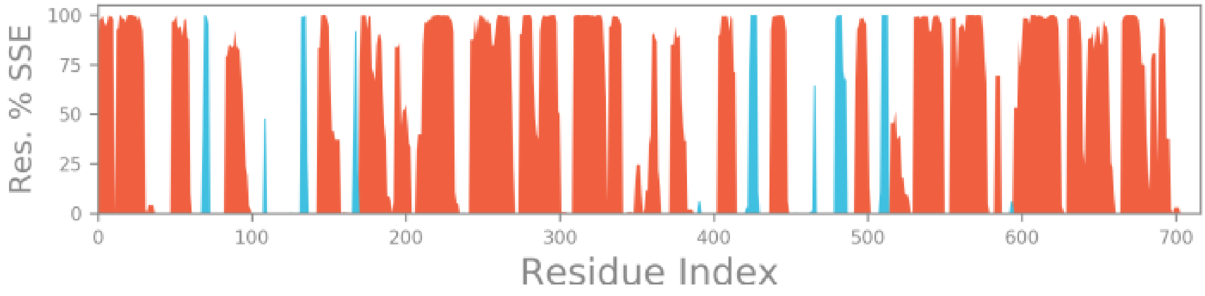
Res. % SSE
100
75
50
25
0
0
100
200
300
400
500
600
700
Residue Index

C4

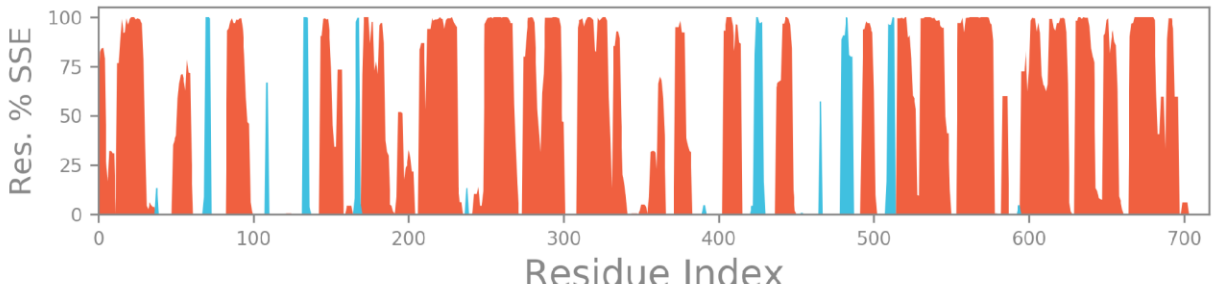
Res. % SSE
100
75
50
25
0
0
100
200
300
400
500
600
700
Residue Index

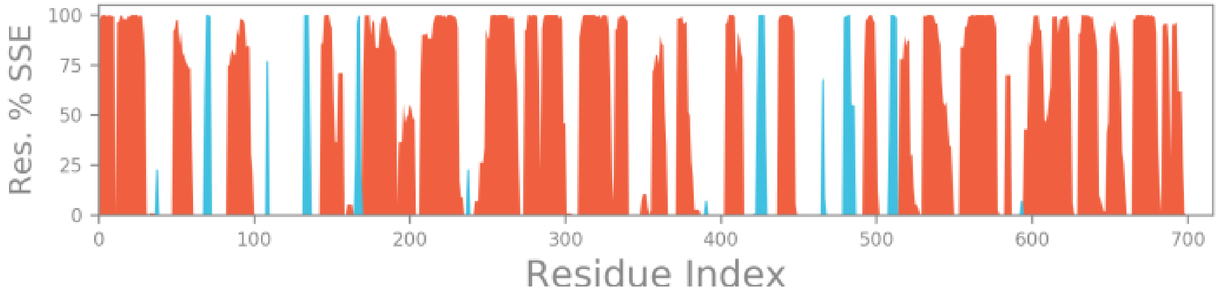
Res. % SSE
100
75
50
25
0
0
100
200
300
400
500
600
700
Residue Index

C5

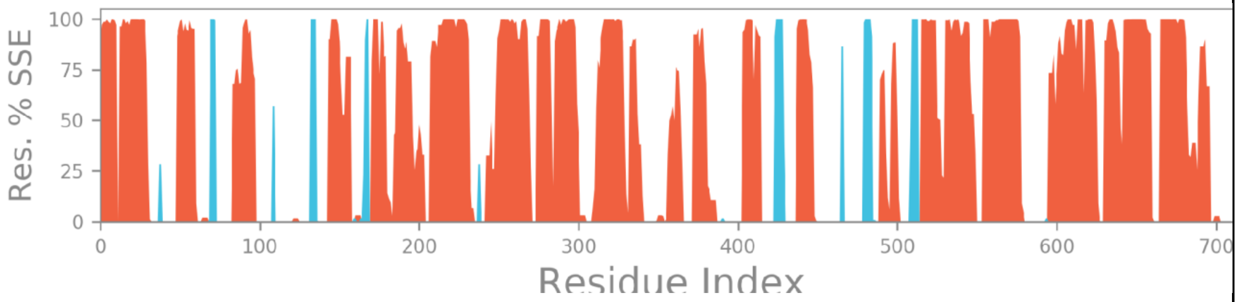
Res. % SSE
100
75
50
25
0
0
100
200
300
400
500
600
700
Residue Index

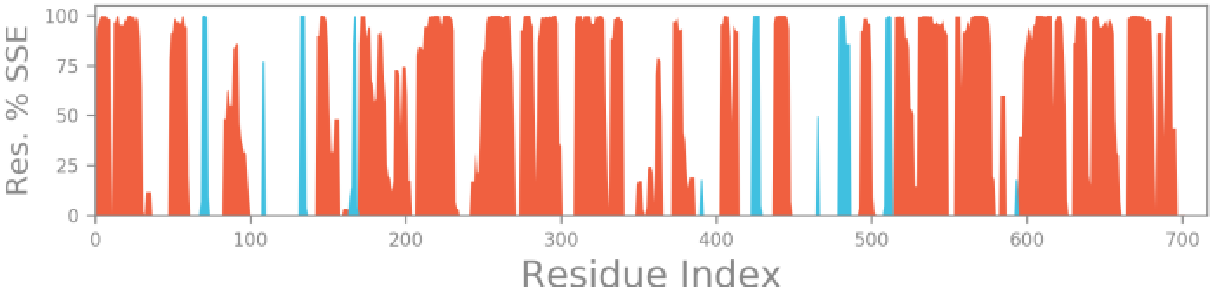
Res. % SSE
100
75
50
25
0
0
100
200
300
400
500
600
700
Residue Index

C6

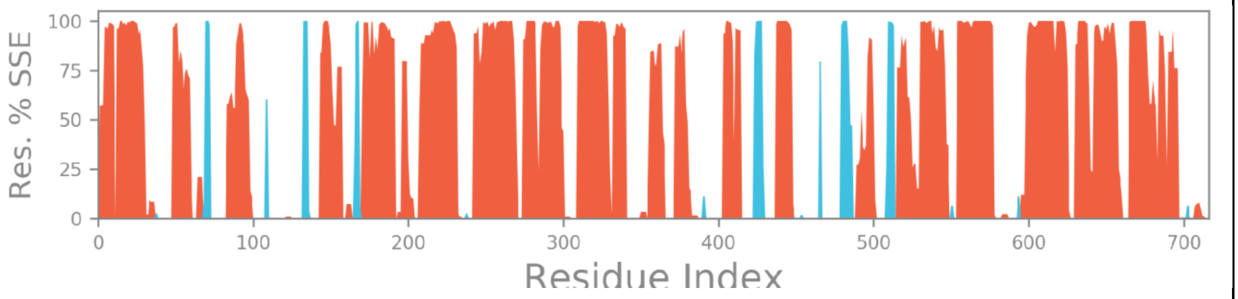
Res. % SSE
100
75
50
25
0
0
100
200
300
400
500
600
700
Residue Index

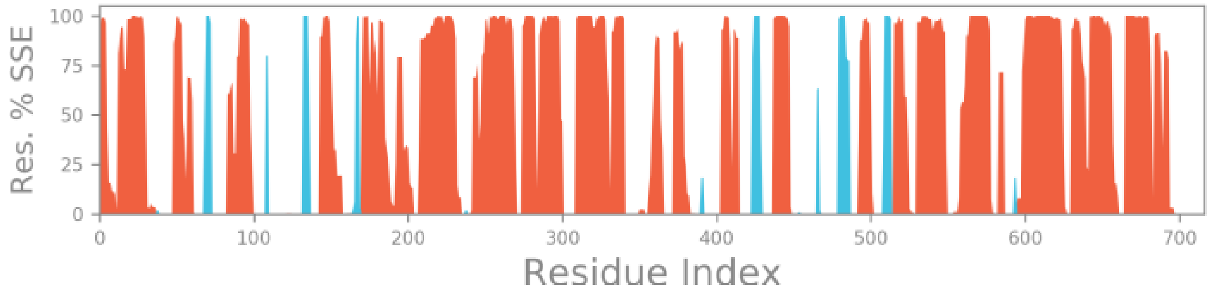
Res. % SSE
100
75
50
25
0
0
100
200
300
400
500
600
700
Residue Index

C7

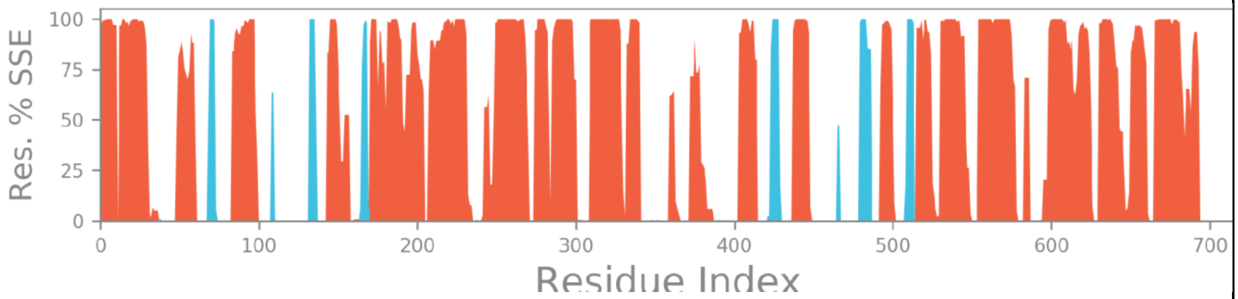
Res. % SSE
100
75
50
25
0
0
100
200
300
400
500
600
700
Residue Index

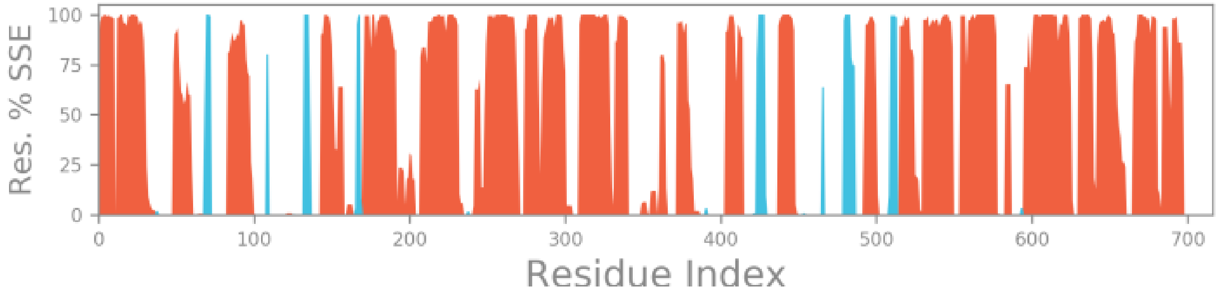
Res. % SSE
100
75
50
25
0
0
100
200
300
400
500
600
700
Residue Index

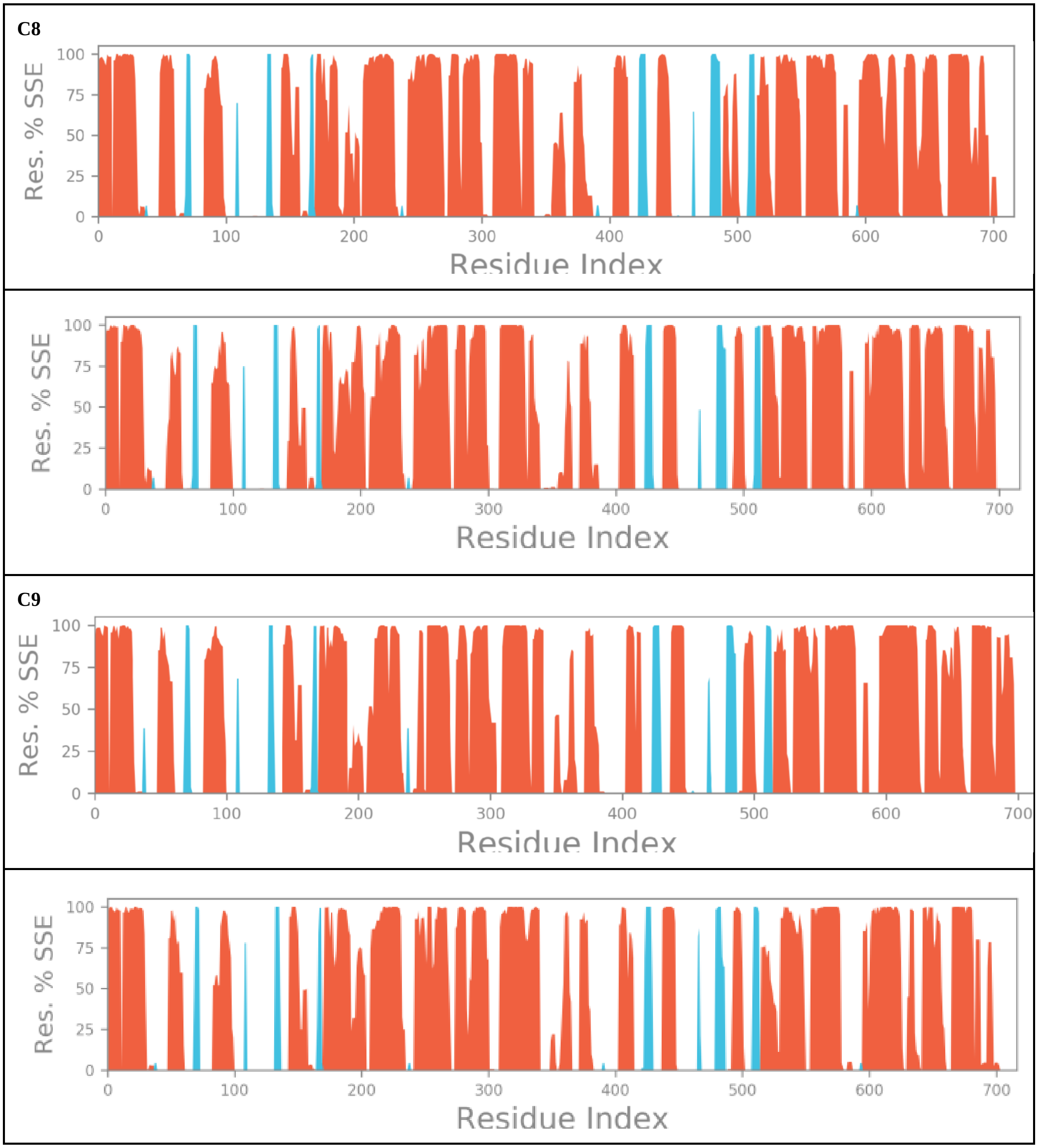

Figure S12. Protein Secondary Structure elements (SSE) for MmpL3 complexed with SQ109 and the top nine compounds. Red represents alpha helices, blue represents beta sheets and white represents random coils.

SQ109

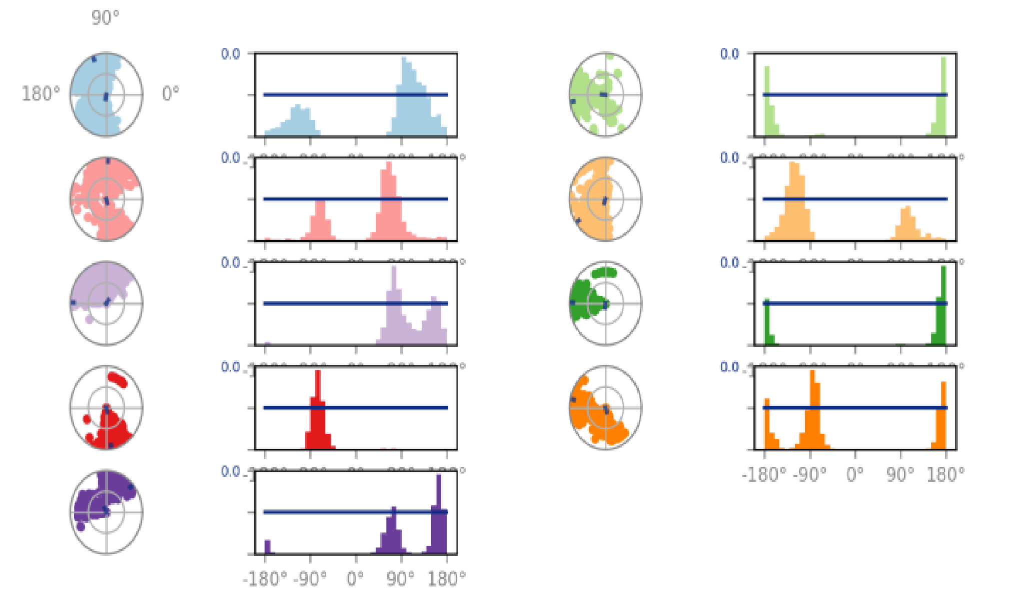

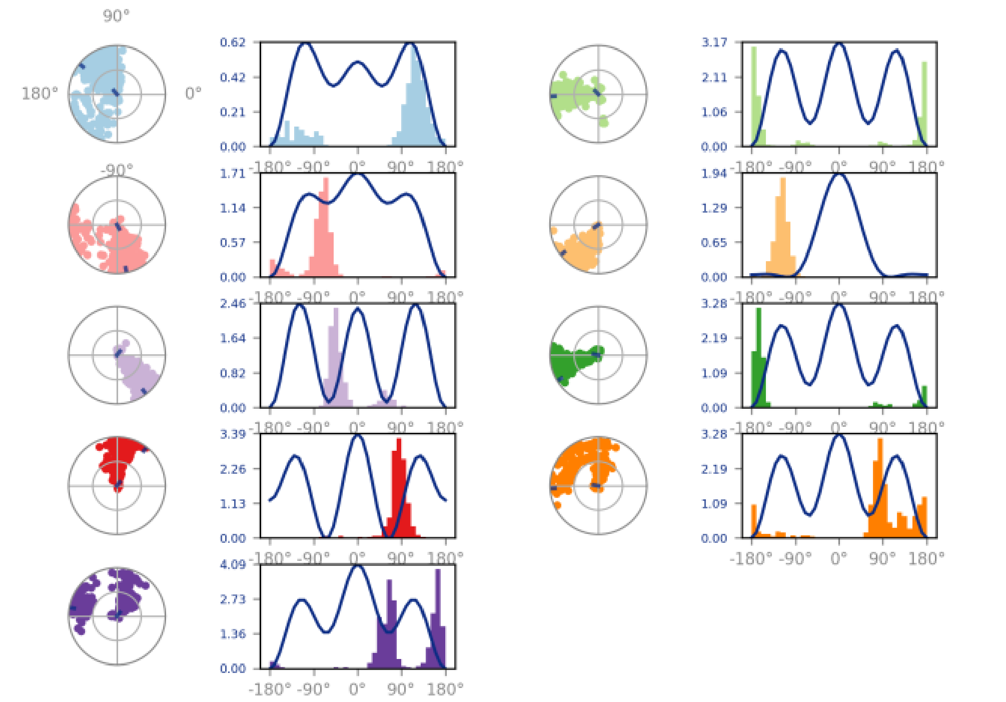

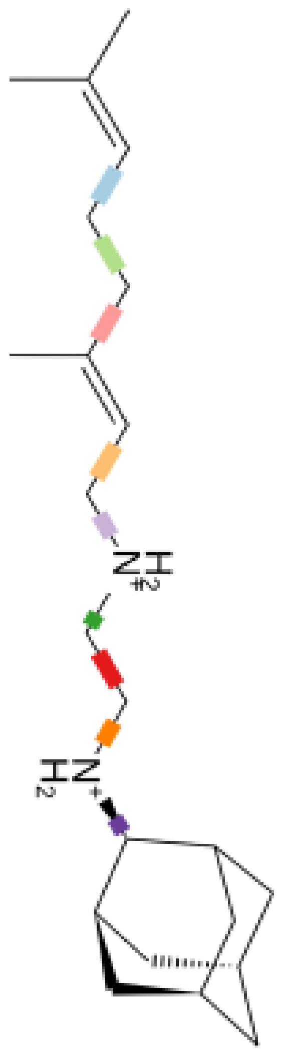

C
1

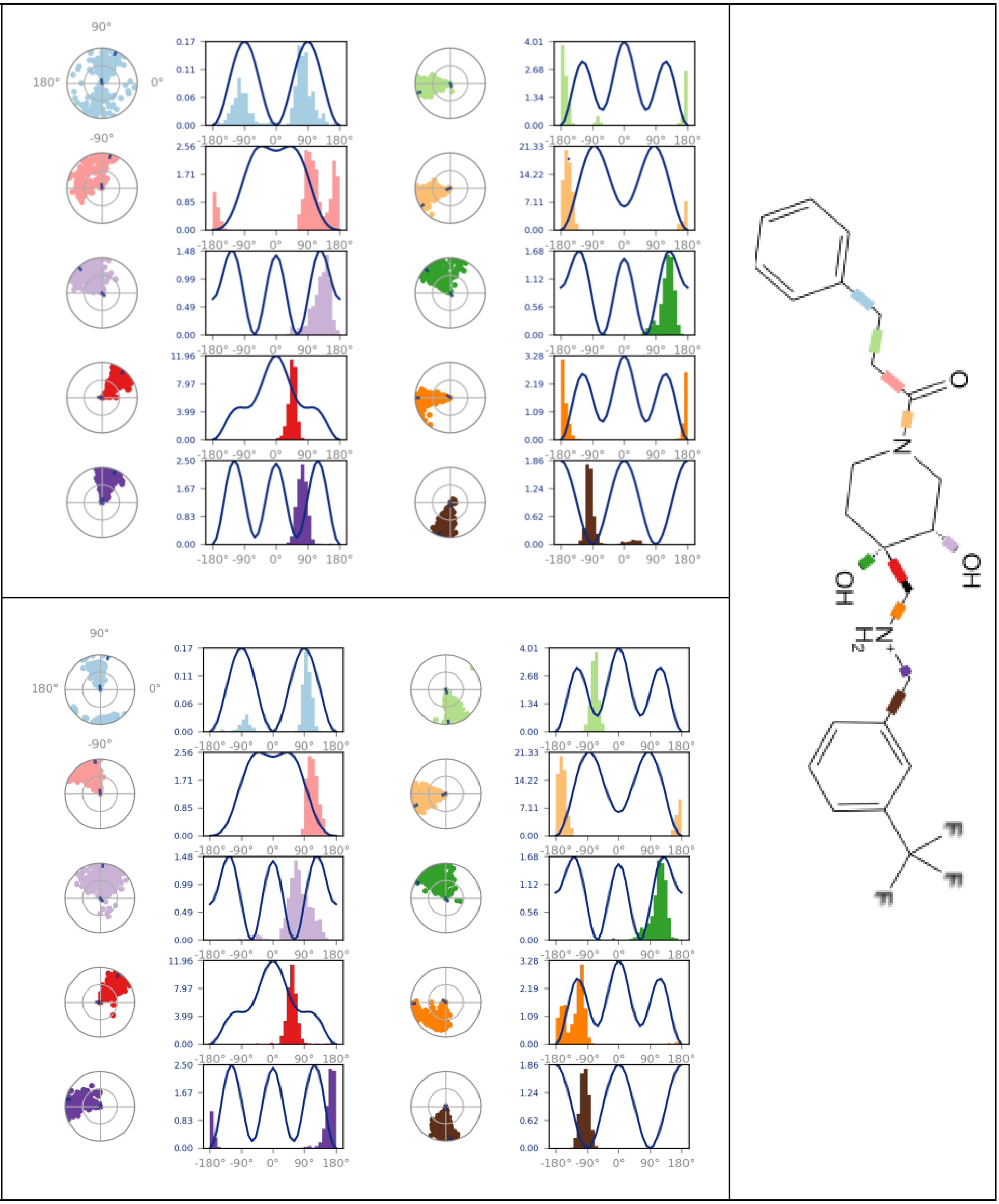

C3

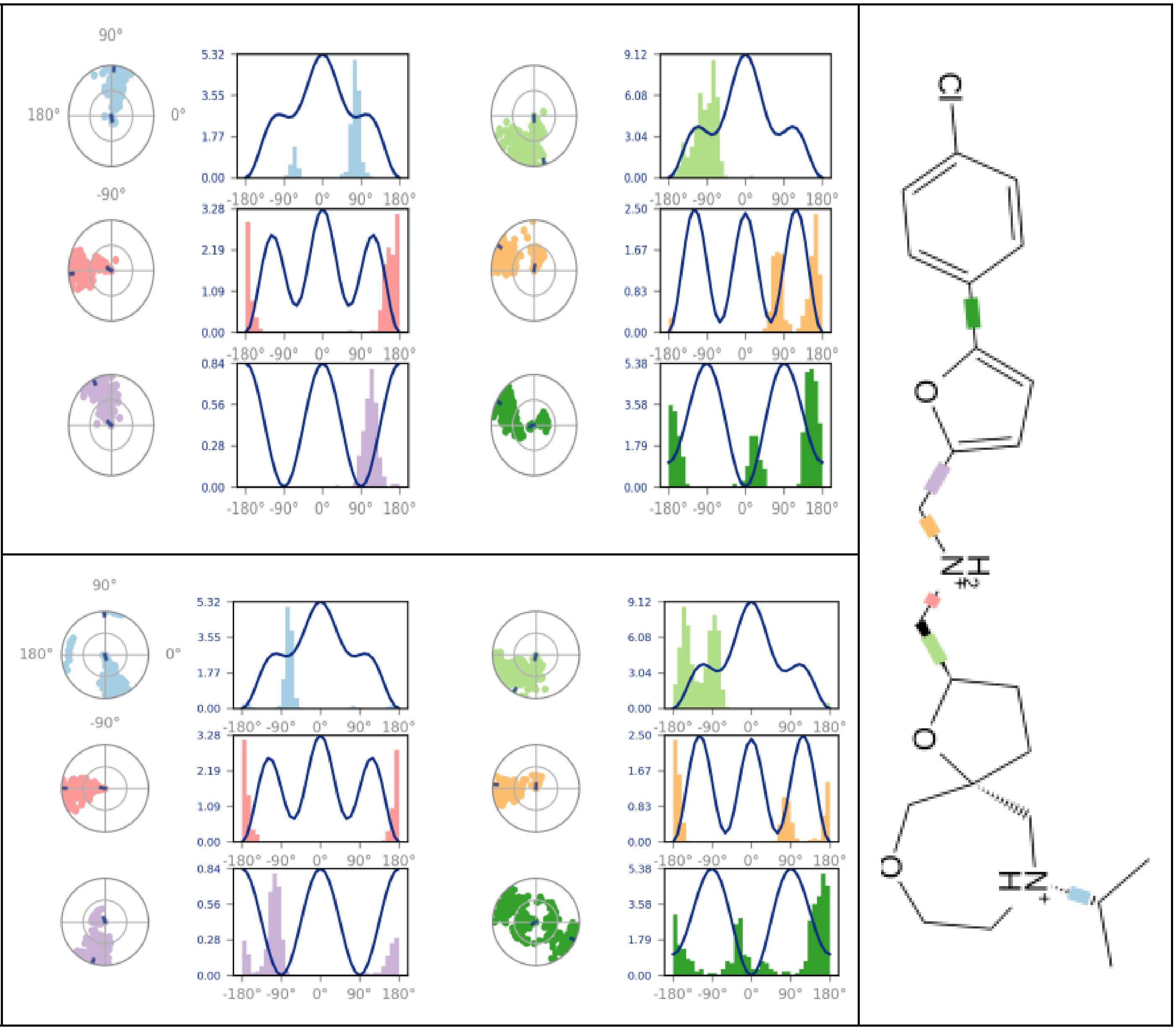
90°
180°
0°
-90°
5.32
3.55
1.77
0.00
9.12
6.08
3.04
3.28
2.19
1.09
2.50
1.67
0.83
0.84
0.56
0.28
5.38
3.58
1.79
-180° -90° 0° 90° 180°
Cl
O
O
O
O

**C 7**	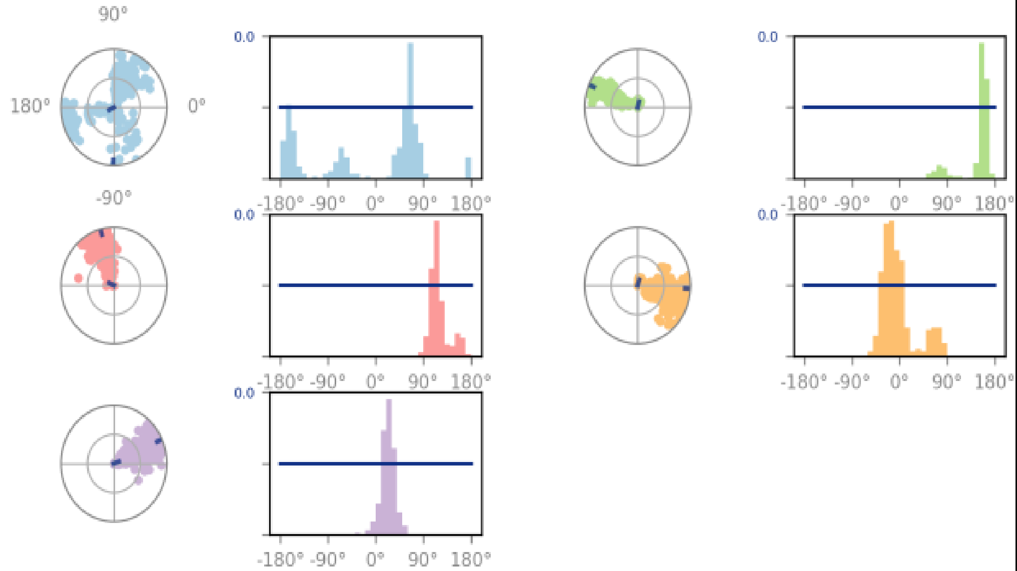90°
180°
0°
-90°
0.0
-180° -90° 0° 90° 180° 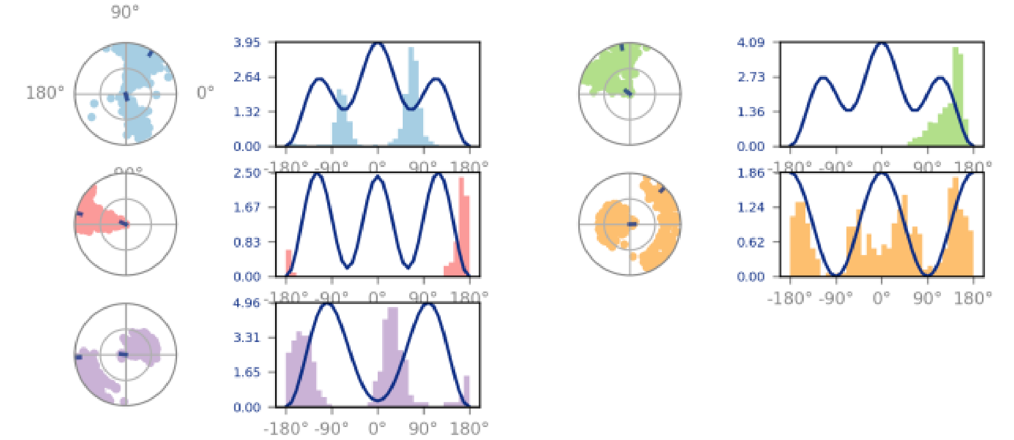90°
180°
0°
3.95
2.64
1.32
0.00
2.50
1.67
0.83
4.96
3.31
1.65
4.09
2.73
1.36
1.86
1.24
0.62
-180° -90° 0° 90° 180°	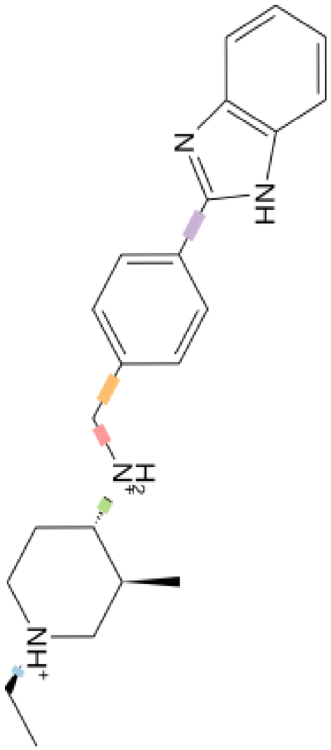N
HN

C
9

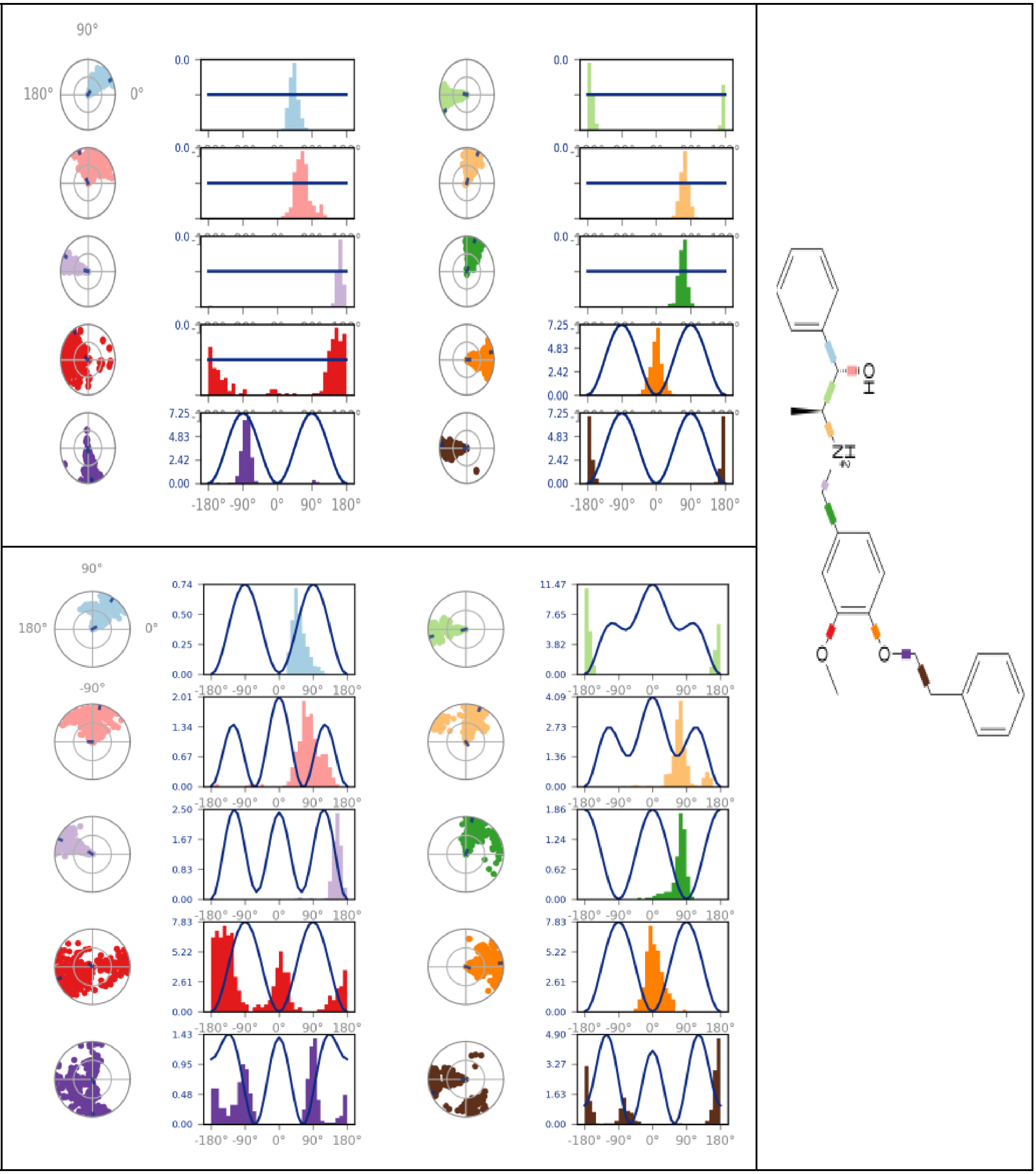

Figure S13. Ligand torsion plot summarizing the conformational progression of the rotatable bonds of SQ109 and the top four ligands throughout the two MmpL3 simulation trajectories (0 to 200 ns). The left panel shows a 2-D schematic which has a color code for each of the rotatable

bonds for each ligand. The associated dial plot and bar plot for each color code summarizes the conformation of the torsion and the probability density of the torsion for each of the rotatable bonds in each ligand. The Y-axis value for the bar plot is expressed in kcal/mol. The right panel is the 2-D structure of SQ109 and the top four ligands, with rotatable bonds highlighted.

SQ109

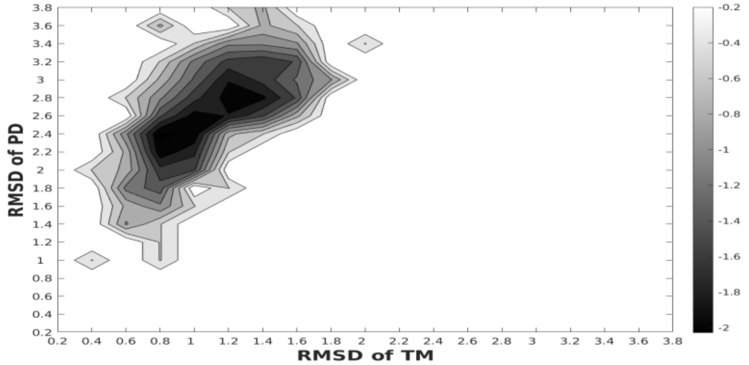

C1

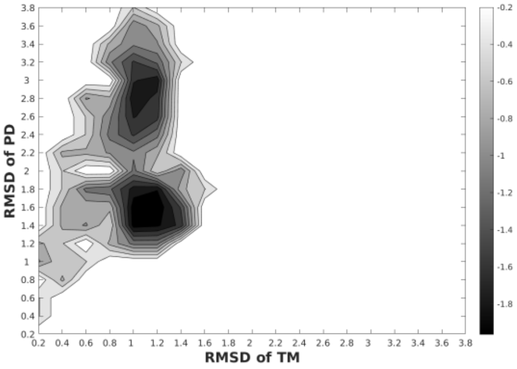

C3

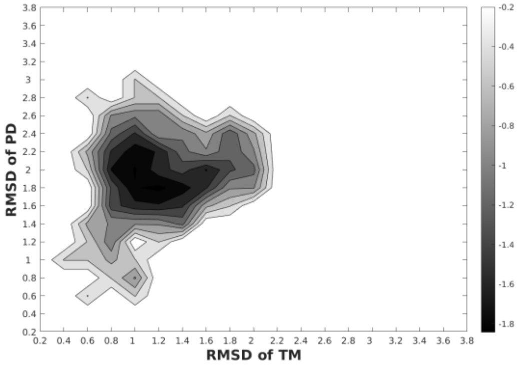

C7

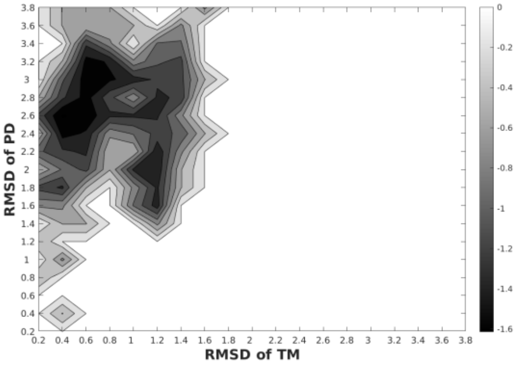

C9

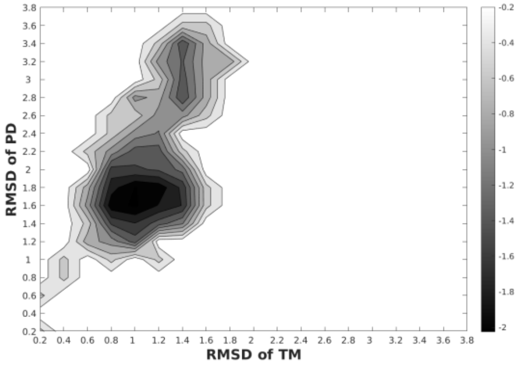

Figure S14 Free energy landscape (unit: kcal/mol) of the 2 trajectories (400ns) of each of SQ109 and our 4 highlighted systems of MMPL3. Lowest energy states are shaded dark while Higher energy states are shaded lighter. RMSD of PD and TMD were calculated through Ca atoms of residues (TMD) residues 1-34, 170-420, 548-748 and Porter Domain (PD) residues 35-169 and 421-547

SQ109

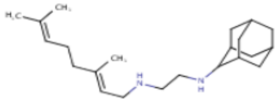

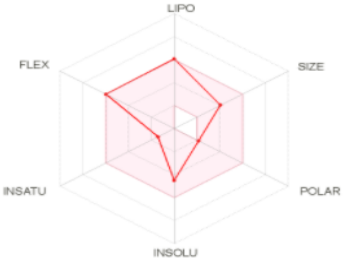

SMILES C/C(=C\CNCCNC1C2CC3CC1CC(C2)C3)/CCC=C(C)C

Physicochemical Properties	
Formula	C22H38N2
Molecular weight	330.55 g/mol
Num. heavy atoms	24
Num. arom. heavy atoms	0
Fraction Csp3	0.82
Num. rotatable bonds	9
Num. H-bond acceptors	2
Num. H-bond donors	2
Molar Refractivity	106.18
TPSA	24.06 Å²

Lipophilicity	
Log $P_{o/w}$ (iLOGP)	4.81
Log $P_{o/w}$ (XLOGP3)	5.11
Log $P_{o/w}$ (WLOGP)	4.68
Log $P_{o/w}$ (MLOGP)	4.26
Log $P_{o/w}$ (SILICOS-IT)	4.61
Consensus Log $P_{o/w}$	4.70

Water Solubility	
Log *S* (ESOL)	-4.51
Solubility	1.01e-02 mg/ml ; 3.06e-05 mol/l
Class	Moderately soluble
Log *S* (Ali)	-5.36
Solubility	1.45e-03 mg/ml ; 4.37e-06 mol/l
Class	Moderately soluble
Log *S* (SILICOS-IT)	-4.94
Solubility	3.81e-03 mg/ml ; 1.15e-05 mol/l
Class	Moderately soluble

Pharmacokinetics	
GI absorption	High
BBB permeant	Yes
P-gp substrate	No
CYP1A2 inhibitor	No
CYP2C19 inhibitor	No
CYP2C9 inhibitor	No
CYP2D6 inhibitor	Yes
CYP3A4 inhibitor	No
Log K_p (skin permeation)	-4.69 cm/s

Druglikeness	
Lipinski	Yes; 1 violation: MLOGP>4.15
Ghose	Yes
Veber	Yes
Egan	Yes
Muegge	No; 1 violation: XLOGP3>5
Bioavailability Score	0.55

Medicinal Chemistry	
PAINS	0 alert
Brenk	1 alert: isolated_alkene
Leadlikeness	No; 2 violations: Rotors>7, XLOGP3>3.5
Synthetic accessibility	5.81

NITD-349

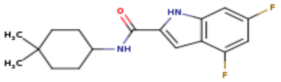

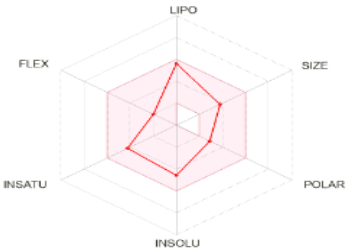

SMILES Fc1cc(F)c2c(c1)[nH]c(c2)C(=O)NC1CCC(CC1)(C)C

Physicochemical Properties	
Formula	C17H20F2N2O
Molecular weight	306.35 g/mol
Num. heavy atoms	22
Num. arom. heavy atoms	9
Fraction Csp3	0.47
Num. rotatable bonds	3
Num. H-bond acceptors	3
Num. H-bond donors	2
Molar Refractivity	82.49
TPSA	44.89 Å²

Lipophilicity	
Log $P_{o/w}$ (iLOGP)	2.79
Log $P_{o/w}$ (XLOGP3)	4.32
Log $P_{o/w}$ (WLOGP)	4.99
Log $P_{o/w}$ (MLOGP)	3.60
Log $P_{o/w}$ (SILICOS-IT)	4.66
Consensus Log $P_{o/w}$	4.07

Water Solubility	
Log *S* (ESOL)	-4.57
Solubility	8.33e-03 mg/ml ; 2.72e-05 mol/l
Class	Moderately soluble
Log *S* (Ali)	-4.98
Solubility	3.23e-03 mg/ml ; 1.05e-05 mol/l
Class	Moderately soluble
Log *S* (SILICOS-IT)	-6.03
Solubility	2.87e-04 mg/ml ; 9.38e-07 mol/l
Class	Poorly soluble

Pharmacokinetics	
GI absorption	High
BBB permeant	Yes
P-gp substrate	Yes
CYP1A2 inhibitor	Yes
CYP2C19 inhibitor	No
CYP2C9 inhibitor	No
CYP2D6 inhibitor	Yes
CYP3A4 inhibitor	No
Log K_p (skin permeation)	-5.10 cm/s

Druglikeness	
Lipinski	Yes; 0 violation
Ghose	Yes
Veber	Yes
Egan	Yes
Muegge	Yes
Bioavailability Score	0.55

Medicinal Chemistry	
PAINS	0 alert
Brenk	0 alert
Leadlikeness	No; 1 violation: XLOGP3>3.5
Synthetic accessibility	2.41

PIPD1

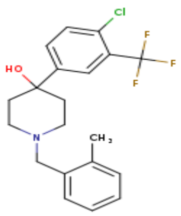

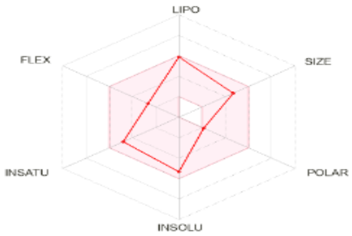

SMILES Cc1ccccc1CN1CCC(CC1)(O)c1ccc(c(c1)C(F)(F)F)Cl

Physicochemical Properties	
Formula	C20H21ClF3NO
Molecular weight	383.84 g/mol
Num. heavy atoms	26
Num. arom. heavy atoms	12
Fraction Csp3	0.40
Num. rotatable bonds	4
Num. H-bond acceptors	5
Num. H-bond donors	1
Molar Refractivity	100.65
TPSA	23.47 Å^2

Lipophilicity	
Log $P_{o/w}$ (iLOGP)	3.55
Log $P_{o/w}$ (XLOGP3)	4.79
Log $P_{o/w}$ (WLOGP)	5.66
Log $P_{o/w}$ (MLOGP)	4.55
Log $P_{o/w}$ (SILICOS-IT)	5.70
Consensus Log $P_{o/w}$	4.85

Water Solubility	
Log *S* (ESOL)	-5.32
Solubility	1.86e-03 mg/ml ; 4.84e-06 mol/l
Class	Moderately soluble
Log *S* (Ali)	-5.01
Solubility	3.71e-03 mg/ml ; 9.67e-06 mol/l
Class	Moderately soluble
Log *S* (SILICOS-IT)	-7.28
Solubility	2.01e-05 mg/ml ; 5.22e-08 mol/l
Class	Poorly soluble

Pharmacokinetics	
GI absorption	High
BBB permeant	Yes
P-gp substrate	Yes
CYP1A2 inhibitor	Yes
CYP2C19 inhibitor	Yes
CYP2C9 inhibitor	No
CYP2D6 inhibitor	Yes
CYP3A4 inhibitor	No
Log K_p (skin permeation)	-5.24 cm/s

Druglikeness	
Lipinski	Yes; 1 violation: MLOGP>4.15
Ghose	No; 1 violation: WLOGP>5.6
Veber	Yes
Egan	Yes
Muegge	Yes
Bioavailability Score	0.55

Medicinal Chemistry	
PAINS	0 alert
Brenk	0 alert
Leadlikeness	No; 2 violations: MW>350, XLOGP3>3.5
Synthetic accessibility	2.49

C215

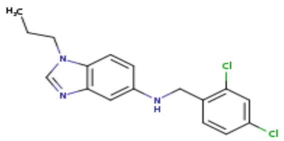

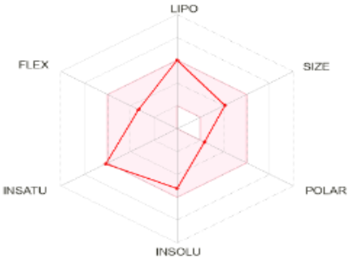

SMILES CCCn1cnc2c1ccc(c2)NCc1ccc(cc1Cl)Cl

Physicochemical Properties	
Formula	C17H17Cl2N3
Molecular weight	334.24 g/mol
Num. heavy atoms	22
Num. arom. heavy atoms	15
Fraction Csp3	0.24
Num. rotatable bonds	5
Num. H-bond acceptors	1
Num. H-bond donors	1
Molar Refractivity	94.42
TPSA	29.85 Å²

Lipophilicity	
Log $P_{o/w}$ (iLOGP)	3.36
Log $P_{o/w}$ (XLOGP3)	5.00
Log $P_{o/w}$ (WLOGP)	5.02
Log $P_{o/w}$ (MLOGP)	3.95
Log $P_{o/w}$ (SILICOS-IT)	4.54
Consensus Log $P_{o/w}$	4.37

Water Solubility	
Log *S* (ESOL)	-5.24
Solubility	1.94e-03 mg/ml ; 5.80e-06 mol/l
Class	Moderately soluble
Log *S* (Ali)	-5.37
Solubility	1.44e-03 mg/ml ; 4.30e-06 mol/l
Class	Moderately soluble
Log *S* (SILICOS-IT)	-7.46
Solubility	1.16e-05 mg/ml ; 3.48e-08 mol/l
Class	Poorly soluble

Pharmacokinetics	
GI absorption	High
BBB permeant	Yes
P-gp substrate	No
CYP1A2 inhibitor	Yes
CYP2C19 inhibitor	Yes
CYP2C9 inhibitor	Yes
CYP2D6 inhibitor	Yes
CYP3A4 inhibitor	Yes
Log K_p (skin permeation)	-4.79 cm/s

Druglikeness	
Lipinski	Yes; 0 violation
Ghose	Yes
Veber	Yes
Egan	Yes
Muegge	Yes
Bioavailability Score	0.55

Medicinal Chemistry	
PAINS	1 alert: anil_alk_bim
Brenk	0 alert
Leadlikeness	No; 1 violation: XLOGP3>3.5
Synthetic accessibility	2.23

C1

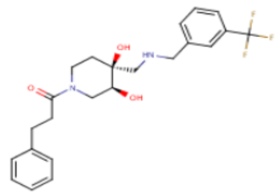

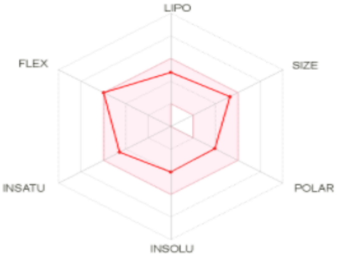

SMILES	O=C(N1CC[C@]([C@H](C1)O)(O)CNCc1cccc(c1)C(F)(F)F)CCc1ccccc1

Physicochemical Properties	
Formula	C23H27F3N2O3
Molecular weight	436.47 g/mol
Num. heavy atoms	31
Num. arom. heavy atoms	12
Fraction Csp3	0.43
Num. rotatable bonds	9
Num. H-bond acceptors	7
Num. H-bond donors	3
Molar Refractivity	114.22
TPSA	72.80 Å²

Lipophilicity	
Log $P_{o/w}$ (iLOGP)	3.29
Log $P_{o/w}$ (XLOGP3)	2.83
Log $P_{o/w}$ (WLOGP)	3.37
Log $P_{o/w}$ (MLOGP)	2.47
Log $P_{o/w}$ (SILICOS-IT)	3.99
Consensus Log $P_{o/w}$	3.19

Water Solubility	
Log *S* (ESOL)	-4.02
Solubility	4.15e-02 mg/ml ; 9.52e-05 mol/l
Class	Moderately soluble
Log *S* (Ali)	-4.02
Solubility	4.20e-02 mg/ml ; 9.62e-05 mol/l
Class	Moderately soluble
Log *S* (SILICOS-IT)	-6.64
Solubility	9.98e-05 mg/ml ; 2.29e-07 mol/l
Class	Poorly soluble

Pharmacokinetics	
GI absorption	High
BBB permeant	Yes
P-gp substrate	Yes
CYP1A2 inhibitor	No
CYP2C19 inhibitor	No
CYP2C9 inhibitor	No
CYP2D6 inhibitor	Yes
CYP3A4 inhibitor	No
Log K_p (skin permeation)	-6.95 cm/s

Druglikeness	
Lipinski	Yes; 0 violation
Ghose	Yes
Veber	Yes
Egan	Yes
Muegge	Yes
Bioavailability Score	0.55

Medicinal Chemistry	
PAINS	0 alert
Brenk	0 alert
Leadlikeness	No; 2 violations: MW>350, Rotors>7
Synthetic accessibility	3.58

C2

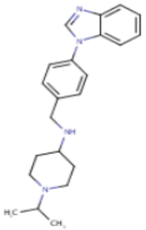

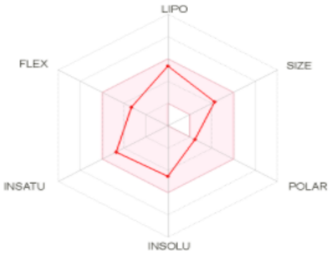

SMILES CC(N1CCC(CC1)NCc1ccc(cc1)n1cnc2c1cccc2)C

Physicochemical Properties	
Formula	C22H28N4
Molecular weight	348.48 g/mol
Num. heavy atoms	26
Num. arom. heavy atoms	15
Fraction Csp3	0.41
Num. rotatable bonds	5
Num. H-bond acceptors	3
Num. H-bond donors	1
Molar Refractivity	111.99
TPSA	33.09 Å²

Lipophilicity	
Log $P_{o/w}$ (iLOGP)	3.93
Log $P_{o/w}$ (XLOGP3)	3.86
Log $P_{o/w}$ (WLOGP)	3.46
Log $P_{o/w}$ (MLOGP)	3.23
Log $P_{o/w}$ (SILICOS-IT)	3.27
Consensus Log $P_{o/w}$	3.55

Water Solubility	
Log *S* (ESOL)	-4.53
Solubility	1.03e-02 mg/ml ; 2.96e-05 mol/l
Class	Moderately soluble
Log *S* (Ali)	-4.25
Solubility	1.95e-02 mg/ml ; 5.60e-05 mol/l
Class	Moderately soluble
Log *S* (SILICOS-IT)	-6.34
Solubility	1.60e-04 mg/ml ; 4.60e-07 mol/l
Class	Poorly soluble

Pharmacokinetics	
GI absorption	High
BBB permeant	Yes
P-gp substrate	Yes
CYP1A2 inhibitor	Yes
CYP2C19 inhibitor	No
CYP2C9 inhibitor	No
CYP2D6 inhibitor	Yes
CYP3A4 inhibitor	No
Log K_p (skin permeation)	-5.69 cm/s

Druglikeness	
Lipinski	Yes; 0 violation
Ghose	Yes
Veber	Yes
Egan	Yes
Muegge	Yes
Bioavailability Score	0.55

Medicinal Chemistry	
PAINS	0 alert
Brenk	0 alert
Leadlikeness	No; 1 violation: XLOGP3>3.5
Synthetic accessibility	2.54

C3

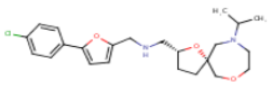

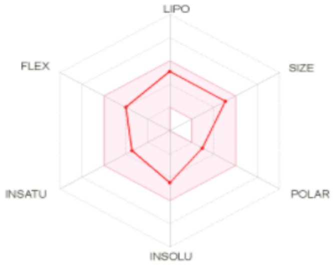

SMILES	Clc1ccc(cc1)c1ccc(o1)CNC[C@H]1CC[C@]2(O1)COCCN(C2)C(C)C

Physicochemical Properties	
Formula	C23H31ClN2O3
Molecular weight	418.96 g/mol
Num. heavy atoms	29
Num. arom. heavy atoms	11
Fraction Csp3	0.57
Num. rotatable bonds	6
Num. H-bond acceptors	5
Num. H-bond donors	1
Molar Refractivity	119.40
TPSA	46.87 Å²

Lipophilicity	
Log $P_{o/w}$ (iLOGP)	4.52
Log $P_{o/w}$ (XLOGP3)	3.44
Log $P_{o/w}$ (WLOGP)	3.82
Log $P_{o/w}$ (MLOGP)	2.08
Log $P_{o/w}$ (SILICOS-IT)	4.63
Consensus Log $P_{o/w}$	3.70

Water Solubility	
Log *S* (ESOL)	-4.49
Solubility	1.36e-02 mg/ml ; 3.24e-05 mol/l
Class	Moderately soluble
Log *S* (Ali)	-4.11
Solubility	3.29e-02 mg/ml ; 7.85e-05 mol/l
Class	Moderately soluble
Log *S* (SILICOS-IT)	-7.13
Solubility	3.13e-05 mg/ml ; 7.46e-08 mol/l
Class	Poorly soluble

Pharmacokinetics	
GI absorption	High
BBB permeant	Yes
P-gp substrate	Yes
CYP1A2 inhibitor	No
CYP2C19 inhibitor	No
CYP2C9 inhibitor	No
CYP2D6 inhibitor	Yes
CYP3A4 inhibitor	Yes
Log K_p (skin permeation)	-6.41 cm/s

Druglikeness	
Lipinski	Yes; 0 violation
Ghose	Yes
Veber	Yes
Egan	Yes
Muegge	Yes
Bioavailability Score	0.55

Medicinal Chemistry	
PAINS	0 alert
Brenk	0 alert
Leadlikeness	No; 1 violation: MW>350
Synthetic accessibility	4.94

C4

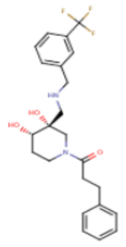

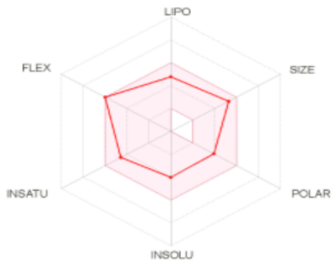

SMILES O=C(N1CC[C@@H]([C@@](C1)(O)CNCc1cccc(c1)C(F)(F)F)O)CCc1ccccc1

Physicochemical Properties	
Formula	C23H27F3N2O3
Molecular weight	436.47 g/mol
Num. heavy atoms	31
Num. arom. heavy atoms	12
Fraction Csp3	0.43
Num. rotatable bonds	9
Num. H-bond acceptors	7
Num. H-bond donors	3
Molar Refractivity	114.22
TPSA	72.80 Å²

Lipophilicity	
Log $P_{o/w}$ (iLOGP)	2.86
Log $P_{o/w}$ (XLOGP3)	2.83
Log $P_{o/w}$ (WLOGP)	3.37
Log $P_{o/w}$ (MLOGP)	2.47
Log $P_{o/w}$ (SILICOS-IT)	3.99
Consensus Log $P_{o/w}$	3.11

Water Solubility	
Log *S* (ESOL)	-4.02
Solubility	4.15e-02 mg/ml ; 9.52e-05 mol/l
Class	Moderately soluble
Log *S* (Ali)	-4.02
Solubility	4.20e-02 mg/ml ; 9.62e-05 mol/l
Class	Moderately soluble
Log *S* (SILICOS-IT)	-6.64
Solubility	9.98e-05 mg/ml ; 2.29e-07 mol/l
Class	Poorly soluble

Pharmacokinetics	
GI absorption	High
BBB permeant	Yes
P-gp substrate	Yes
CYP1A2 inhibitor	No
CYP2C19 inhibitor	No
CYP2C9 inhibitor	No
CYP2D6 inhibitor	Yes
CYP3A4 inhibitor	Yes
Log K_p (skin permeation)	-6.95 cm/s

Druglikeness	
Lipinski	Yes; 0 violation
Ghose	Yes
Veber	Yes
Egan	Yes
Muegge	Yes
Bioavailability Score	0.55

Medicinal Chemistry	
PAINS	0 alert
Brenk	0 alert
Leadlikeness	No; 2 violations: MW>350, Rotors>7
Synthetic accessibility	3.61

C5

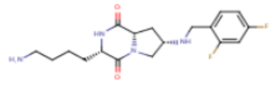

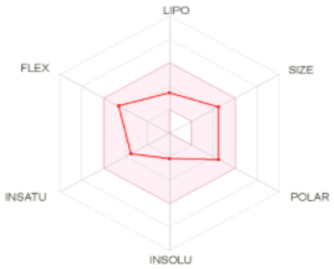

SMILES	NCCCC[C@@H]1NC(=O)[C@H]2N(C1=O)C[C@H](C2)NCc1ccc(cc1F)F

Physicochemical Properties	
Formula	C18H24F2N4O2
Molecular weight	366.41 g/mol
Num. heavy atoms	26
Num. arom. heavy atoms	6
Fraction Csp3	0.56
Num. rotatable bonds	7
Num. H-bond acceptors	6
Num. H-bond donors	3
Molar Refractivity	99.41
TPSA	87.46 Å²

Lipophilicity	
Log $P_{o/w}$ (iLOGP)	2.88
Log $P_{o/w}$ (XLOGP3)	0.54
Log $P_{o/w}$ (WLOGP)	0.58
Log $P_{o/w}$ (MLOGP)	1.30
Log $P_{o/w}$ (SILICOS-IT)	2.03
Consensus Log $P_{o/w}$	1.46

Water Solubility	
Log *S* (ESOL)	-2.16
Solubility	2.53e+00 mg/ml ; 6.91e-03 mol/l
Class	Soluble
Log *S* (Ali)	-1.95
Solubility	4.13e+00 mg/ml ; 1.13e-02 mol/l
Class	Very soluble
Log *S* (SILICOS-IT)	-5.04
Solubility	3.38e-03 mg/ml ; 9.23e-06 mol/l
Class	Moderately soluble

Pharmacokinetics	
GI absorption	High
BBB permeant	No
P-gp substrate	Yes
CYP1A2 inhibitor	No
CYP2C19 inhibitor	No
CYP2C9 inhibitor	No
CYP2D6 inhibitor	No
CYP3A4 inhibitor	No
Log K_p (skin permeation)	-8.15 cm/s

Druglikeness	
Lipinski	Yes; 0 violation
Ghose	Yes
Veber	Yes
Egan	Yes
Muegge	Yes
Bioavailability Score	0.55

Medicinal Chemistry	
PAINS	0 alert
Brenk	0 alert
Leadlikeness	No; 1 violation: MW>350
Synthetic accessibility	3.53

C6

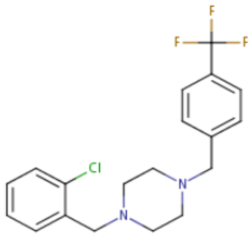

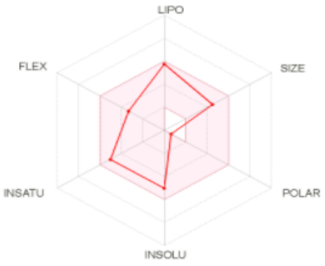

SMILES Clc1ccccc1CN1CCN(CC1)Cc1ccc(cc1)C(F)(F)F

Physicochemical Properties	
Formula	C19H20ClF3N2
Molecular weight	368.82 g/mol
Num. heavy atoms	25
Num. arom. heavy atoms	12
Fraction Csp3	0.37
Num. rotatable bonds	5
Num. H-bond acceptors	5
Num. H-bond donors	0
Molar Refractivity	101.45
TPSA	6.48 Å²

Lipophilicity	
Log $P_{o/w}$ (iLOGP)	3.90
Log $P_{o/w}$ (XLOGP3)	4.58
Log $P_{o/w}$ (WLOGP)	4.76
Log $P_{o/w}$ (MLOGP)	4.34
Log $P_{o/w}$ (SILICOS-IT)	4.95
Consensus Log $P_{o/w}$	4.51

Water Solubility	
Log *S* (ESOL)	-5.04
Solubility	3.38e-03 mg/ml ; 9.18e-06 mol/l
Class	Moderately soluble
Log *S* (Ali)	-4.44
Solubility	1.34e-02 mg/ml ; 3.63e-05 mol/l
Class	Moderately soluble
Log *S* (SILICOS-IT)	-6.96
Solubility	4.09e-05 mg/ml ; 1.11e-07 mol/l
Class	Poorly soluble

Pharmacokinetics	
GI absorption	High
BBB permeant	Yes
P-gp substrate	No
CYP1A2 inhibitor	Yes
CYP2C19 inhibitor	Yes
CYP2C9 inhibitor	No
CYP2D6 inhibitor	Yes
CYP3A4 inhibitor	No
Log K_p (skin permeation)	-5.30 cm/s

Druglikeness	
Lipinski	Yes; 1 violation: MLOGP>4.15
Ghose	Yes
Veber	Yes
Egan	Yes
Muegge	Yes
Bioavailability Score	0.55

Medicinal Chemistry	
PAINS	0 alert
Brenk	0 alert
Leadlikeness	No; 2 violations: MW>350, XLOGP3>3.5
Synthetic accessibility	2.32

C7

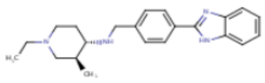

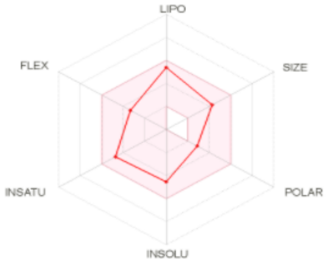

SMILES CCN1CC[C@@H]([C@H](C1)C)NCc1ccc(cc1)c1nc2c([nH]1)cccc2

Physicochemical Properties	
Formula	C22H28N4
Molecular weight	348.48 g/mol
Num. heavy atoms	26
Num. arom. heavy atoms	15
Fraction Csp3	0.41
Num. rotatable bonds	5
Num. H-bond acceptors	3
Num. H-bond donors	2
Molar Refractivity	112.45
TPSA	43.95 Å²
**Lipophilicity**	
Log $P_{o/w}$ (iLOGP)	3.34
Log $P_{o/w}$ (XLOGP3)	3.91
Log $P_{o/w}$ (WLOGP)	3.52
Log $P_{o/w}$ (MLOGP)	3.23
Log $P_{o/w}$ (SILICOS-IT)	4.20
Consensus Log $P_{o/w}$	3.64

Water Solubility	
Log *S* (ESOL)	-4.56
Solubility	9.58e-03 mg/ml ; 2.75e-05 mol/l
Class	Moderately soluble
Log *S* (Ali)	-4.53
Solubility	1.02e-02 mg/ml ; 2.94e-05 mol/l
Class	Moderately soluble
Log *S* (SILICOS-IT)	-7.29
Solubility	1.80e-05 mg/ml ; 5.15e-08 mol/l
Class	Poorly soluble
**Pharmacokinetics**	
GI absorption	High
BBB permeant	Yes
P-gp substrate	Yes
CYP1A2 inhibitor	Yes
CYP2C19 inhibitor	Yes
CYP2C9 inhibitor	No
CYP2D6 inhibitor	Yes
CYP3A4 inhibitor	Yes
Log K_p (skin permeation)	-5.65 cm/s
**Druglikeness**	
Lipinski	Yes; 0 violation
Ghose	Yes
Veber	Yes
Egan	Yes
Muegge	Yes
Bioavailability Score	0.55
**Medicinal Chemistry**	
PAINS	0 alert
Brenk	0 alert
Leadlikeness	No; 1 violation: XLOGP3>3.5
Synthetic accessibility	3.19

C8

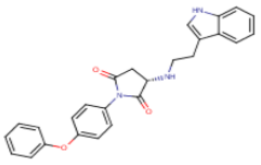

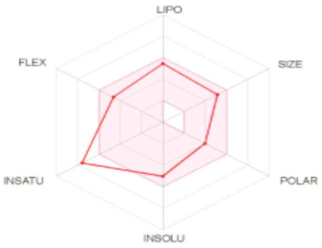

SMILES	O=C1C[C@@H](C(=O)N1c1ccc(cc1)Oc1ccccc1)NCCc1c[nH]c2c1cccc2
**Physicochemical Properties**	
Formula	C26H23N3O3
Molecular weight	425.48 g/mol
Num. heavy atoms	32
Num. arom. heavy atoms	21
Fraction Csp3	0.15
Num. rotatable bonds	7
Num. H-bond acceptors	4
Num. H-bond donors	2
Molar Refractivity	126.76
TPSA	74.43 Å²
**Lipophilicity**	
Log $P_{o/w}$ (iLOGP)	3.38
Log $P_{o/w}$ (XLOGP3)	4.02
Log $P_{o/w}$ (WLOGP)	4.04
Log $P_{o/w}$ (MLOGP)	3.14
Log $P_{o/w}$ (SILICOS-IT)	4.38
Consensus Log $P_{o/w}$	3.79

**Water Solubility**	
Log *S* (ESOL)	-5.03
Solubility	3.93e-03 mg/ml ; 9.24e-06 mol/l
Class	Moderately soluble
Log *S* (Ali)	-5.29
Solubility	2.20e-03 mg/ml ; 5.18e-06 mol/l
Class	Moderately soluble
Log *S* (SILICOS-IT)	-8.81
Solubility	6.59e-07 mg/ml ; 1.55e-09 mol/l
Class	Poorly soluble
**Pharmacokinetics**	
GI absorption	High
BBB permeant	Yes
P-gp substrate	Yes
CYP1A2 inhibitor	Yes
CYP2C19 inhibitor	Yes
CYP2C9 inhibitor	Yes
CYP2D6 inhibitor	Yes
CYP3A4 inhibitor	Yes
Log K_p (skin permeation)	-6.04 cm/s
**Druglikeness**	
Lipinski	Yes; 0 violation
Ghose	Yes
Veber	Yes
Egan	Yes
Muegge	Yes
Bioavailability Score	0.55
**Medicinal Chemistry**	
PAINS	0 alert
Brenk	1 alert: phthalimide
Leadlikeness	No; 2 violations: MW>350, XLOGP3>3.5
Synthetic accessibility	3.31

C9

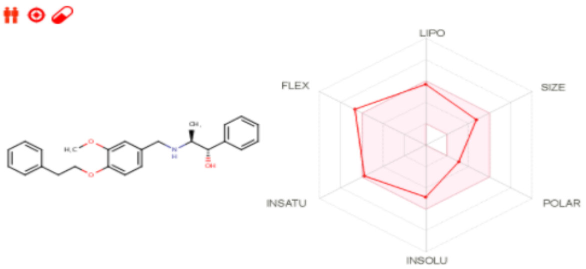

SMILES COc1cc(CN[C@H]([C@H](c2ccccc2)O)C)ccc1OCCc1ccccc1

Physicochemical Properties	
Formula	C25H29NO3
Molecular weight	391.50 g/mol
Num. heavy atoms	29
Num. arom. heavy atoms	18
Fraction Csp3	0.28
Num. rotatable bonds	10
Num. H-bond acceptors	4
Num. H-bond donors	2
Molar Refractivity	116.56
TPSA	50.72 Å²
**Lipophilicity**	
Log $P_{o/w}$ (iLOGP)	3.81
Log $P_{o/w}$ (XLOGP3)	4.47
Log $P_{o/w}$ (WLOGP)	4.05
Log $P_{o/w}$ (MLOGP)	3.51
Log $P_{o/w}$ (SILICOS-IT)	5.24
Consensus Log $P_{o/w}$	4.22

Water Solubility	
Log *S* (ESOL)	-4.88
Solubility	5.13e-03 mg/ml ; 1.31e-05 mol/l
Class	Moderately soluble
Log *S* (Ali)	-5.25
Solubility	2.18e-03 mg/ml ; 5.56e-06 mol/l
Class	Moderately soluble
Log *S* (SILICOS-IT)	-8.40
Solubility	1.54e-06 mg/ml ; 3.94e-09 mol/l
Class	Poorly soluble
**Pharmacokinetics**	
GI absorption	High
BBB permeant	Yes
P-gp substrate	Yes
CYP1A2 inhibitor	No
CYP2C19 inhibitor	Yes
CYP2C9 inhibitor	No
CYP2D6 inhibitor	Yes
CYP3A4 inhibitor	Yes
Log K_p (skin permeation)	-5.51 cm/s
**Druglikeness**	
Lipinski	Yes; 0 violation
Ghose	Yes
Veber	Yes
Egan	Yes
Muegge	Yes
Bioavailability Score	0.55
**Medicinal Chemistry**	
PAINS	0 alert
Brenk	0 alert
Leadlikeness	No; 3 violations: MW>350, Rotors>7, XLOGP3>3.5
Synthetic accessibility	3.56

Figure S15. Predicted ADMET properties for the top nine best compounds including the reference compound SQ109 and other known inhibitors of MmpL3 obtained from the SwissADME server.
